# Gene dysregulation by histone variant H2A.Z in bladder cancer

**DOI:** 10.1186/1756-8935-6-34

**Published:** 2013-10-16

**Authors:** Kyunghwan Kim, Vasu Punj, Jongkyu Choi, Kyu Heo, Jin-Man Kim, Peter W Laird, Woojin An

**Affiliations:** 1 Department of Biochemistry and Molecular Biology, Norris Comprehensive Cancer Center, 1450 Biggy Street, Los Angeles, CA 90033, USA; 2 Bioinformatics Core and Division of Hematology, Norris Comprehensive Cancer Center, 1450 Biggy Street, Los Angeles, CA 90033, USA; 3 USC Epigenome Center, University of Southern California Keck School of Medicine, 1450 Biggy Street, Los Angeles, CA 90033, USA; 4 Research Center, Dongnam Institute of Radiological and Medical Sciences, 40 Jwadong-gil, Gijang-gun, Busan 619-953, South Korea

**Keywords:** Histone, H2A.Z, Chromatin, Bladder cancer, ChIP-seq, Microarray, Gene expression

## Abstract

**Background:**

The incorporation of histone variants into nucleosomes is one of the main strategies that the cell uses to regulate the structure and function of chromatin. Histone H2A.Z is an evolutionarily conserved histone H2A variant that is preferentially localized within nucleosomes at the transcriptional start site (TSS). H2A.Z reorganizes the local chromatin structure and recruits the transcriptional machinery for gene activation. High expression of H2A.Z has been reported in several types of cancers and is causally linked to genomic instability and tumorigenesis. However, it is not entirely clear how H2A.Z overexpression in cancer cells establishes aberrant chromatin states and promotes gene expression.

**Results:**

Through integration of genome-wide H2A.Z ChIP-seq data with microarray data, we demonstrate that H2A.Z is enriched around the TSS of cell cycle regulatory genes in bladder cancer cells, and this enrichment is correlated with the elevated expression of cancer-promoting genes. RNAi-mediated knockdown of H2A.Z in the cancer cells causes transcriptional suppression of multiple cell cycle regulatory genes with a distinct decrease in cell proliferation. H2A.Z nucleosomes around the TSS have higher levels of H3K4me2/me3, which coincides with the recruitment of two chromatin factors, WDR5 and BPTF. The observed recruitment is functional, as the active states of H2A.Z target genes are largely erased by suppressing the expression of WDR5 or BPTF, effects resembling H2A.Z knockdown.

**Conclusions:**

We conclude that H2A.Z is overexpressed in bladder cancer cells and contributes to cancer-related transcription pathways. We also provide evidence in support of the engagement of H3K4me2/me3 and WDR5/BPTF in H2A.Z-induced cancer pathogenesis. Further studies are warranted to understand how H2A.Z overexpression contributes to the recruitment of the full repertoire of transcription machinery to target genes in bladder cancer cells.

## Background

Gene expression in eukaryotes takes place in the context of chromatin, which essentially acts as a physical barrier to the binding of transcription factors to certain genomic regions. The basic unit of chromatin is the nucleosome, which consists of approximately 147 bp of DNA wrapped around an octamer of four core histones (H2A, H2B, H3, and H4) [1]. Despite its high degree of compaction, chromatin structure is dynamically modulated to allow access of genomic DNA during vital cellular processes. Several remodeling processes that alter chromatin structure have been identified and categorized based on their modes of action. The three most prominent processes are histone modification, histone variant exchange and adenosine triphosphate (ATP)-dependent chromatin remodeling [2-5]. Histone variants are nonallelic isoforms of canonical core histones that have been identified primarily from the H2A and H3 families in numerous organisms. Although expressed in smaller amounts than canonical histones, histone variants are involved in a variety of cellular activities with specific genomic localization patterns.

H2A.Z is an essential variant of the histone H2A and has distinct functions in regulating chromatin dynamics. At the amino acid sequence level, H2A.Z shares about 60% identity with canonical H2A in mammalian cells [6]. As for other histone variants, the replacement of histone H2A with H2A.Z is replication-independent and is catalyzed by ATP-dependent exchange factors. H2A.Z preferentially localizes at the transcription start site (TSS) where it frequently flanks nucleosome-deficient regions [7-11]. The presence of H2A.Z at the −1 and +1 nucleosomes adjacent to the TSS has been linked to dynamic changes in gene expression [12-15], but the underlying mechanism is not fully understood. Since H2A.Z-containing nucleosomes wrap DNA more weakly than canonical H2A nucleosomes, they are more susceptible to nuclease digestion and salt-induced destabilization [16,17]. This structural instability is most likely driven by amino acid substitutions at the interface between H2A.Z and H3/H4 [18]. Consistent with its effects on gene transcription, the presence of H2A.Z correlates with active histone modifications and RNA Pol II occupancy at gene promoters, which in turn influence transcriptional output [7,13,19]. These observations are in agreement with the concept that H2A.Z creates an active transcription environment by altering DNA accessibility in chromatin and facilitating the recruitment of transcription regulators. Of special relevance to the present study, overexpression and redistribution of H2A.Z have been noted as epigenetic processes to stimulate the development and progression of certain types of cancer [20-23]. The oncogenic function of H2A.Z is mediated through increased cell proliferation caused by cell cycle deregulation. Therefore, the important questions to address are how H2A.Z maintains chromatin in a certain state, and whether H2A.Z overexpression observed in cancer cells drives the inappropriate activation of cancer-related genes.

In this work, we exploited microarray and chromatin immunoprecipitation sequencing (ChIP-seq) to examine the impact of H2A.Z overexpression on transcriptional states of bladder cancer cells, and identified groups of genes, the dysregulation of which is linked to H2A.Z enrichment around the TSS. We also found that H2A.Z nucleosomes are enriched for the active histone modification H3K4me2/me3 that is likely responsible for recruiting WDR5 and BPTF to H2A.Z target genes.

## Results

### H2A.Z is overexpressed in bladder cancer cells and stimulates cell proliferation

**Figure 1:**
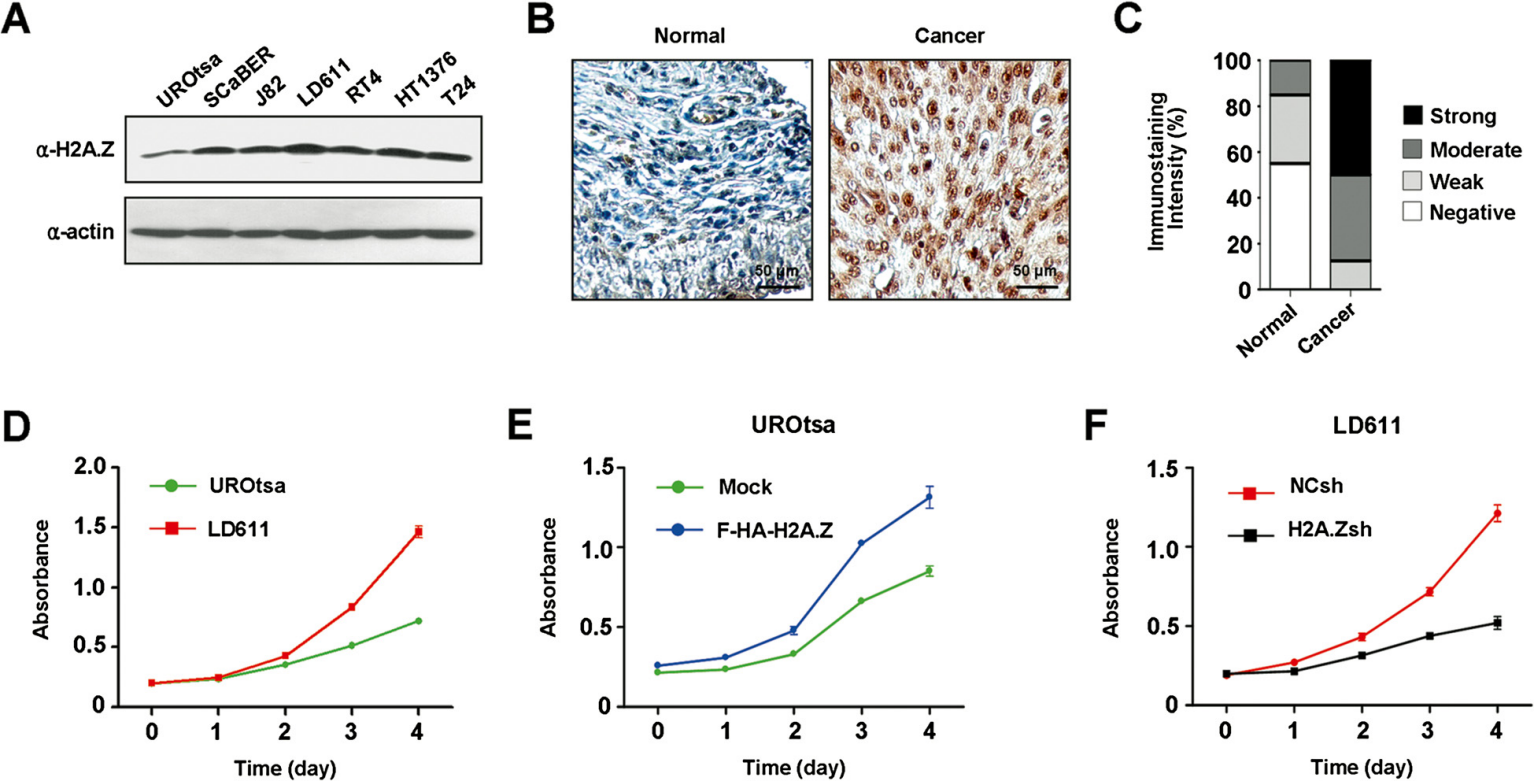
**H2A.Z overexpression and bladder cancer cell growth. (A)** Normal (UROtsa) and bladder cancer (SCaBER, J82, LD611, RT4, HT1376 and T24) cells were lysed in RIPA buffer, and equal amounts of lysates were analyzed by Western blot using anti-H2A.Z antibody. Actin served as a control for equal protein loading. **(B)** Tissue microarrays containing 16 bladder tumor and 8 normal tissue samples were subjected to immunohistochemistry with H2A.Z antibody. Representative high-powered images are shown. **(C)** Immunostaining scores of H2A.Z in normal and bladder cancer tissues. The graph indicates the percentage of sections with different scores (negative, weak, moderate and strong). **(D)** Proliferation of UROtsa and LD611 cells was determined by MTT colorimetric assays at the indicated time points. Data shown represent mean ± SD from three replicates. **(E)** UROtsa cells were transfected with H2A.Z as in Additional file 1: Figure S1, and cell proliferation was measured by MTT assay over a period of four days. All reactions were performed in triplicate. **(F)** LD611 cells were mock-depleted or depleted of H2A.Z as in Additional file 1: Figure S2, and cell proliferation was monitored by MTT assays over a period of four days. MTT assay, 3–4, 5-(dimethyl-thyazol-2-yl)-2, 5-diphenyltetrazolium assay.

As H2A.Z expression is often misregulated in cancer cells, we first examined H2A.Z levels in human bladder cell lines by Western blot. H2A.Z was expressed at much higher levels in the six bladder cancer cell lines (SCaBER, J82, LD611, RT4, HT1376 and T24) compared to the normal bladder cell line UROtsa (Figure 1A). Another demonstration of the relationship between H2A.Z overexpression and tumorigenesis came from the results obtained from immunohistochemical analysis of tissue microarrays containing 16 bladder cancer samples and 8 normal control samples. As illustrated in Figures 1B and 1C, the staining for H2A.Z was strong or moderate in the majority of bladder tumor tissues (approximately 90%), while the staining was weak or negative in most normal tissues (approximately 85%).

We next checked whether the high-level expression of H2A.Z in cancer cells contributes to cell growth over a period of four days. The 3–4, 5-(dimethyl-thyazol-2-yl)-2, 5-diphenyltetrazolium (MTT) assays reproducibly showed that LD611 cancer cells expressing high levels of H2A.Z grow much faster compared to normal UROtsa cells (Figure 1D). These findings were further corroborated by the results showing that the ectopic expression of H2A.Z led to a significant increase in the growth of UROtsa cells (Figure 1E and Additional file 1: Figure S1). To evaluate the effects of H2A.Z overexpression more directly, endogenous H2A.Z was depleted in LD611 cells by using a lentiviral shRNA infection system, and changes in cell growth were analyzed. Western blotting confirmed that the stable transfection of H2A.Z shRNA efficiently silenced H2A.Z expression in the cell (Additional file 1: Figure S2). The MTT assays clearly showed that suppressing H2A.Z expression decreased the growth of LD611 cells (Figure 1F). These observations are consistent with the hypothesis that H2A.Z is one of the key players governing proliferation of bladder cancer cells.

### H2A.Z shows a unique localization pattern in bladder cancer cells

**Figure 2:**
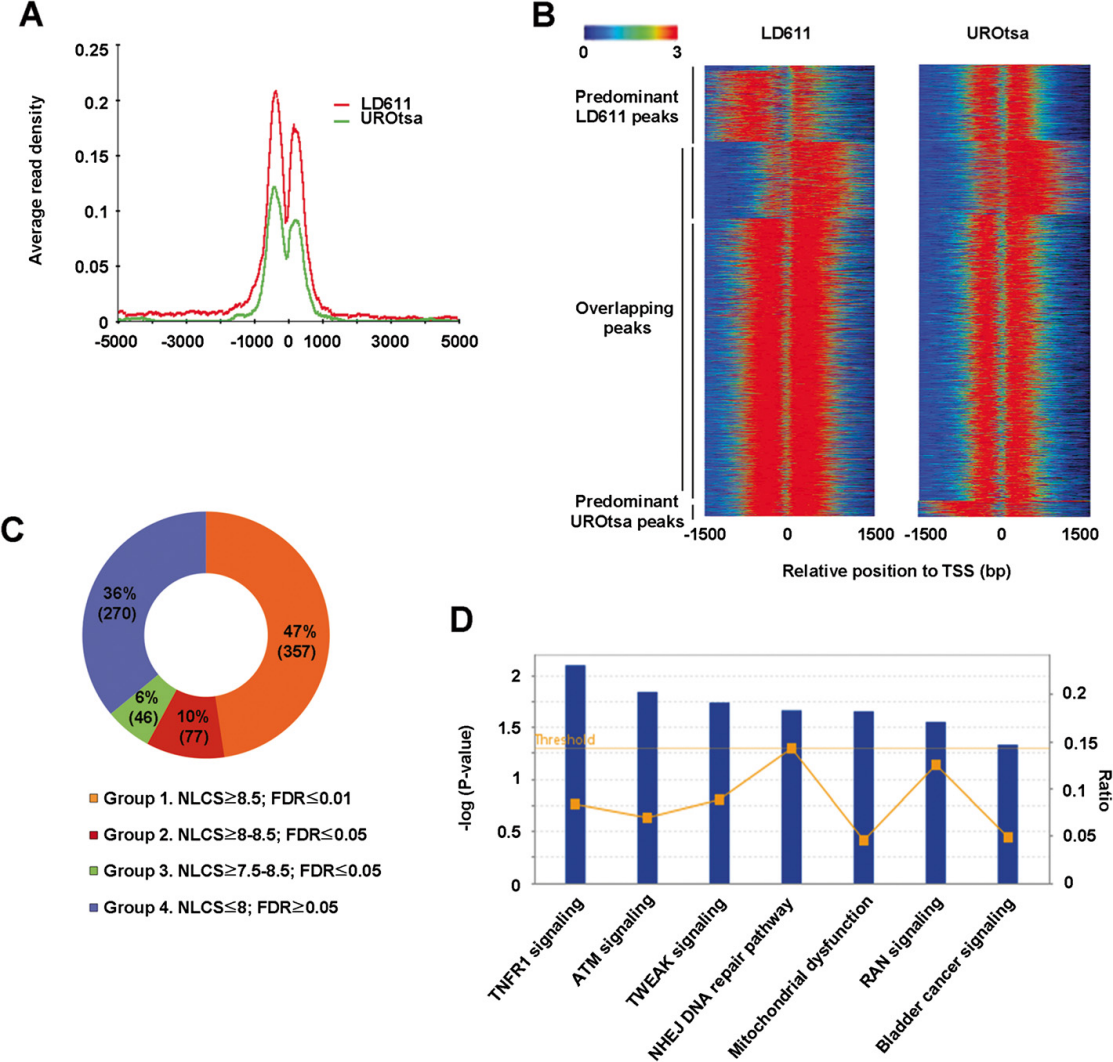
**H2A.Z localization patterns in bladder cancer cells. (A)** Average chromatin immunoprecipitation (ChIP) enrichment signals are shown over regions spanning 10 kb around the transcription start sites (TSSs) of all the human genes from UCSC Refseq database. Green and red lines indicate the level of H2A.Z enrichment in normal UROtsa and cancer LD611 cells, respectively. **(B)** Tag density plots display H2A.Z distribution relative to the TSS. Several representative peaks specific for LD611 and UROtsa cells were also shown. Each horizontal line represents an individual gene, and the enrichment values for H2A.Z are illustrated by color density. **(C)** H2A.Z-enriched genes in LD611 cells were classified into four distinct groups based on false discovery rate (FDR) and the level of locus-specific chromatin state (NLCS) density. Groups 1 and 2 show NLCS ratio ≥8.5 and 8 to 8.5 with FDR ≤0.01 and FDR ≤0.05, respectively. Group 3 has FDR ≤0.05 with NLCS level from 7.5 to 8.5. Group 4 has FDR ≥0.05 with NLCS ≤8. **(D)** H2A.Z-enriched genes in LD611 cells were subjected to ingenuity pathway analysis. Seven top biological functions including bladder cancer signaling were identified with a threshold set at *P* = 0.05*.*

To gain mechanistic insights into the observed effects of H2A.Z, we conducted ChIP-seq of H2A.Z in the LD611 cancer cell line. The UROtsa cell line was used as a normal control to identify the genomic redistribution of H2A.Z in the cancer cell line. We compared the average ChIP enrichment signals over the regions spanning 10 kb around the TSS of all human genes as detailed in the Methods. As expected, H2A.Z occupied nucleosomes surrounding the TSS in both normal and cancer cells (Figure 2A). However, the enrichment of H2A.Z was more pronounced in the bladder cancer cells than in the normal cells. Heatmap analysis with k-means clustering of ChIP-seq datasets also indicated that H2A.Z nucleosomes were more highly enriched in the immediate upstream and downstream of the TSS in the cancer cells (Figure 2B). To distinguish the H2A.Z enrichment from the genomic background, the expectation maximization-based curve fitting method that uses the normalized locus-specific chromatin state (NLCS) was employed as detailed previously [24]. Based on the log_2_ transformed NLCS values and false discovery rate (FDR), we found that 750 and 200 genes were enriched for H2A.Z in the cancer and normal cells, respectively. To identify the genes involved in bladder cancer development, we further classified the cancer-specific genes into the four distinct groups based on their FDR values and NLCS. A total of 480 out of 750 genes were classified into the groups 1, 2 and 3 with *P* <0.05, suggesting that these genes are specifically enriched for H2A.Z in the bladder cancer cells (Figure 2C and Additional file 2: Table S1). To validate ChIP-seq results, we chose three H2A.Z-enriched genes in the normal and cancer cells and carried out conventional ChIP assays. Expectedly, H2A.Z was detected around the TSS of these genes in both cell lines (Additional file 1: Figure S4). Ingenuity pathway analysis also identified a set of genes related to bladder cancer signaling as well as DNA repair (Figure 2D).

### H2A.Z redistribution correlates with increased expression of proliferation-related genes

**Figure 3:**
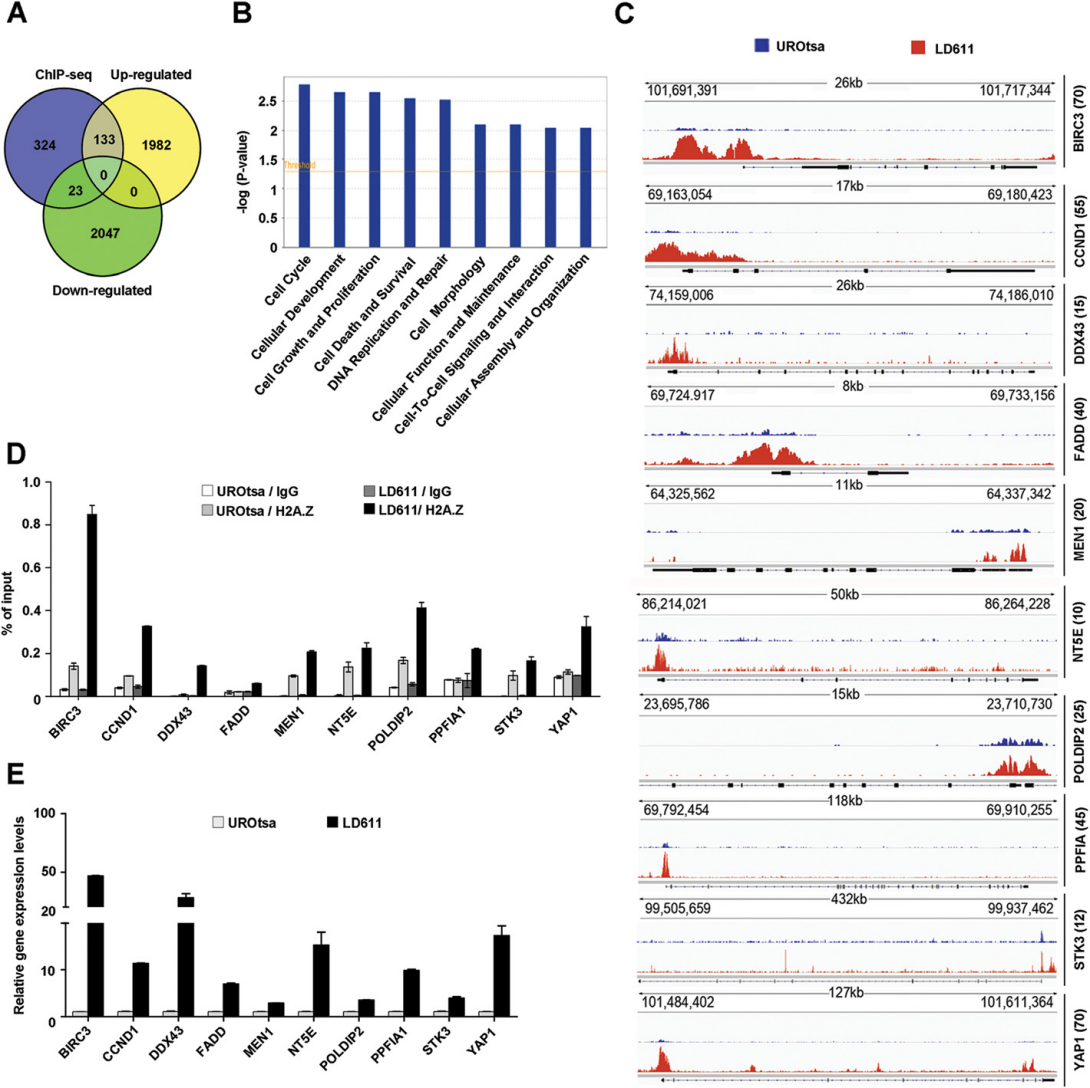
**Effects of H2A.Z enrichment on gene expression. (A)** Venn diagram shows H2A.Z-enriched genes that are upregulated (133 genes) and downregulated (23 genes) more than 1.5-fold with a *P* value of <0.05 in LD611 bladder cells. **(B)** Functional groups associated with the 133 genes that are enriched by H2A.Z and are upregulated in LD611 cancer cells. Biological processes are graphically depicted by *P* value. **(C)** Representative Integrative Genomics Viewer (IGV) genome browser tracks show the levels of H2A.Z near the TSS in LD611 (red) and UROtsa (blue) cells. Maximum sequencing depth over the displayed area is indicated on the right side in parentheses along with name of the genes. Different genomic coordinates and genome window size for *BIRC3* (chr11:101,691,391-101,717,344; 26 kb); *CCND1* (chr11:69,163,054-69,180,423; 17 kb); *DDX43* (chr6:74,159,006-74,186,010; 26 kb); *FADD* (chr11:69,724,917-69,733,156; 8 kb); *MEN1* (chr11:64,325,562-64,337,342; 11 kb); *NT5E* (chr6:86,214,021-86,264,228; 50 kb); *POLDIP2* (chr17:23,695,786-23,710,730; 15 kb); *PPFIA1* (chr11:69,792,454-69,910,255; 118 kb); *STK3* (chr8:99,505,659-99,937,462; 432 kb) and *YAP1* (chr11:101,484,402-101,611,364; 127 kb) are shown along with Reference Sequence (RefSeq) data. **(D)** LD611 and UROtsa cells were analyzed by quantitative chromatin immunoprecipitation (ChIP) using H2A.Z antibody to validate ChIP-sequencing results. Immunoprecipitated DNA was amplified by quantitative PCR (qPCR) with primers specific for the 10 genes. The primers are listed in Additional file 6: Table S5. Percent input was determined as the amount of immunoprecipitated DNA relative to input DNA. Error bars represent standard deviation of three independent experiments. **(E)** The RNA samples from LD611 and UROtsa cells were analyzed by quantitative reverse transcription PCR (qRT-PCR) using primers listed in Additional file 6: Table S5. Expression level was normalized to that of actin, and average and standard deviation of three independent experiments are shown.

The aberrant enrichment of H2A.Z within nucleosomes adjacent to the TSS in the bladder cancer cells suggests that H2A.Z may establish distinct transcription states. To interrogate this possibility, we conducted gene expression profiling with total RNA isolated from the normal and cancer cell lines. Our detailed comparison of the transcriptional profiles identified a large number of differentially expressed genes in the bladder cancer cells, with 2,115 genes being upregulated and 2,070 genes being downregulated (Figure 3A and Additional file 3: Table S2). Quantitative reverse transcription polymerase chain reaction (qRT-PCR) analyses of six upregulated, six downregulated and six unaffected genes validated our microarray results (Additional file 1: Figure S5). To infer which of the genes showing altered expression in the cancer cells were potentially regulated by H2A.Z, we combined the ChIP-seq and gene expression data. Among the 2,115 genes upregulated in the cancer cells, 133 genes exhibited higher levels of H2A.Z. Conversely, only 23 genes out of the 2,069 downregulated genes showed H2A.Z enrichment (Figure 3A and Additional file 4: Table S3). In light of the fact that the acquisition of H2A.Z around the TSS is mainly correlated with gene activation, we decided to focus on the upregulated genes. Functional annotation of differentially expressed genes using ingenuity pathway analysis indicated that genes involved in cell growth and proliferation are significantly enriched in this category (Figure 3B).

When we analyzed a group of the positively regulated proto-oncogenes (*BIRC3, CCND, DDX43, FADD, MEN1, NT5E, POLDIP2, PPFIA1, STK3,* and *YAP1*), we detected a robust signal for H2A.Z around the TSS (Figure 3C, LD611). In the normal bladder cells, these genes displayed much lower levels of H2A.Z (UROtsa). To further validate the ChIP-seq and gene expression data, we selected the 10 active genes from the H2A.Z regulon and performed conventional ChIP assays. Expectedly, H2A.Z enrichment was evident in these genes in the bladder cancer cells, but similar assays in the normal cells showed minimal accumulation of H2A.Z (Figure 3D). Moreover, there was a significant correlation between H2A.Z enrichment and gene activation, as evidenced by qRT-PCR analysis (Figure 3E). To the best of our knowledge, this is the first report linking H2A.Z enrichment to active transcription states in bladder cancer cells.

### WDR5 and BPTF preferentially interact with H2A.Z nucleosomes

**Figure 4:**
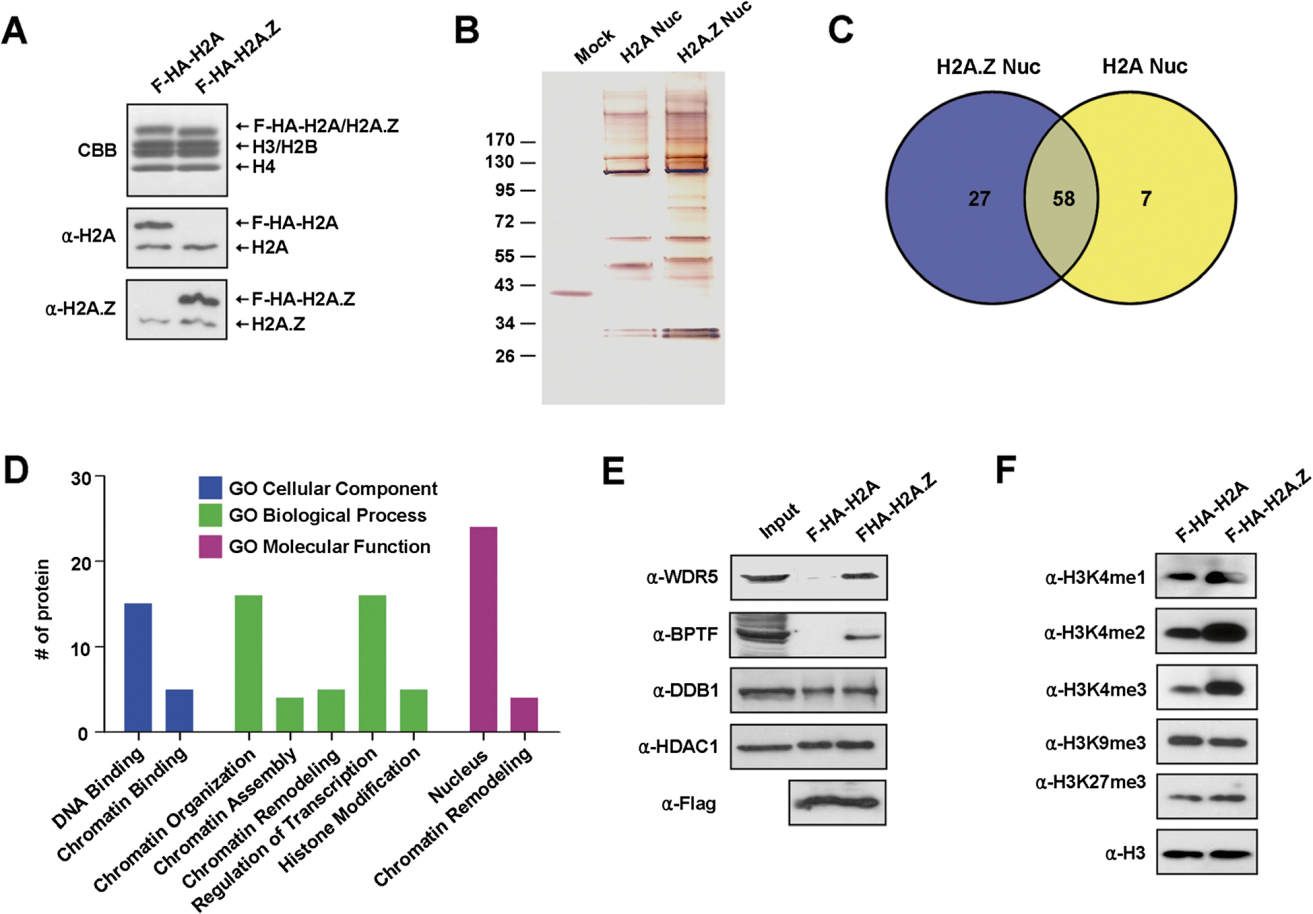
**Preferential binding of WDR5 and BPTF to H2A.Z nucleosomes. (A)** LD611 cells were transfected with H2A and H2A.Z expression vectors, and mononucleosomes were prepared by MNase digestion as recently described [25]. Mononucleosomes containing ectopic H2A (lane 1) and H2A.Z (lane 2) were sequentially immunoprecipitated from total mononucleosomes with anti-Flag and anti-HA antibodies. Histone compositions of the purified nucleosomes were analyzed by 15% SDS-PAGE followed by Coomassie brilliant blue (CBB) staining. The relative levels of endogenous/ectopic H2A and H2A.Z in the purified nucleosomes were determined by Western blotting using H2A and H2A.Z antibodies. **(B)** The proteins co-purified with H2A and H2A.Z nucleosomes were resolved in 4 to 20% SDS-polyacrylamide gels, and then visualized by silver staining. **(C)** Venn diagram depicts the number of proteins associated with H2A nucleosomes (65), H2A.Z nucleosomes (85) and those in common (58). **(D)** The proteins specifically co-purified with H2A.Z nucleosomes were functionally classified by gene ontology analysis. The number of proteins in each functional group is shown. **(E)** The preferential binding of WDR5 and BPTF to H2A.Z nucleosomes were analyzed by Western blotting. **(F)** Mononucleosomes containing ectopic H2A or H2A.Z were purified as in **(A)**, and subjected to Western blotting with antibodies specific for the indicated histone modifications.

To study how H2A.Z directs oncogenic transcription in the bladder cancer cells, we decided to identify proteins that selectively interact with H2A.Z nucleosomes. Accordingly, the LD611 cells were transfected with plasmids expressing Flag/HA-tagged versions of H2A and H2A.Z. After confirming the comparable expression of ectopic H2A.Z and H2A (data not shown), soluble chromatin was prepared from the transfected cells and digested with micrococcal nuclease to yield mainly mononucleosomes. Mononucleosomes containing ectopic histones and their bound proteins were purified by sequential immunoprecipitations with anti-Flag and anti-HA antibodies under stringent conditions (300 mM NaCl and 0.1% NP40). Coomassie brilliant blue (CBB) staining confirmed the stoichiometry of H2B, H3, H4 and ectopic H2A/H2A.Z in the purified nucleosomes (Figure 4A, CBB). Western blotting with H2A and H2A.Z antibodies also indicated that ectopic H2A and H2A.Z were incorporated into nucleosomes at levels substantially higher than their endogenous counterparts (α-H2A and α-H2A.Z). Silver staining of an SDS-polyacrylamide gel containing the proteins that were co-purified with H2A or H2A.Z nucleosomes revealed the presence of multiple extra bands specific to H2A.Z nucleosomes. To identify these extra polypeptides, the purified proteins were subjected to liquid chromatography-tandem mass spectrometry (LC-MS/MS). The data were filtered by excluding any proteins observed in control pull downs with Flag-HA-tag. This approach identified 27 proteins specific to H2A.Z nucleosome pull downs (Figure 4C and Additional file 5: Table S4). Gene ontology analysis of the proteins revealed a striking overrepresentation of proteins related to chromatin reorganization and transcriptional control (Figure 4D).

A noteworthy observation emerged from our purification was substantial interactions of H2A.Z nucleosomes with WDR5 and BPTF, which are known to interact with H3K4me tails and play important roles in regulating chromatin function [26-28]. The observed interactions are specific, as H2A nucleosomes failed to interact with these two factors (Figure 4E). Further analyses revealed that the overall levels of neither H3K9me3 nor H3K27me3 were altered in H2A.Z nucleosomes (Figure 4F, α-H3K9me3 and α-H3K27me3). However, in the same nucleosome samples, H2A.Z nucleosomes showed much higher levels of H3K4me2/me3 as compared to H2A nucleosomes (α-H3K4me2 and α-H3K4me3). These results underscore the notion that the observed binding of WDR5 and BPTF depends on their ability to recognize H2A.Z-facilitated H3K4me2/me3 in the nucleosome.

### H2A.Z-mediated gene activation requires WDR5 and BPTF

**Figure 5:**
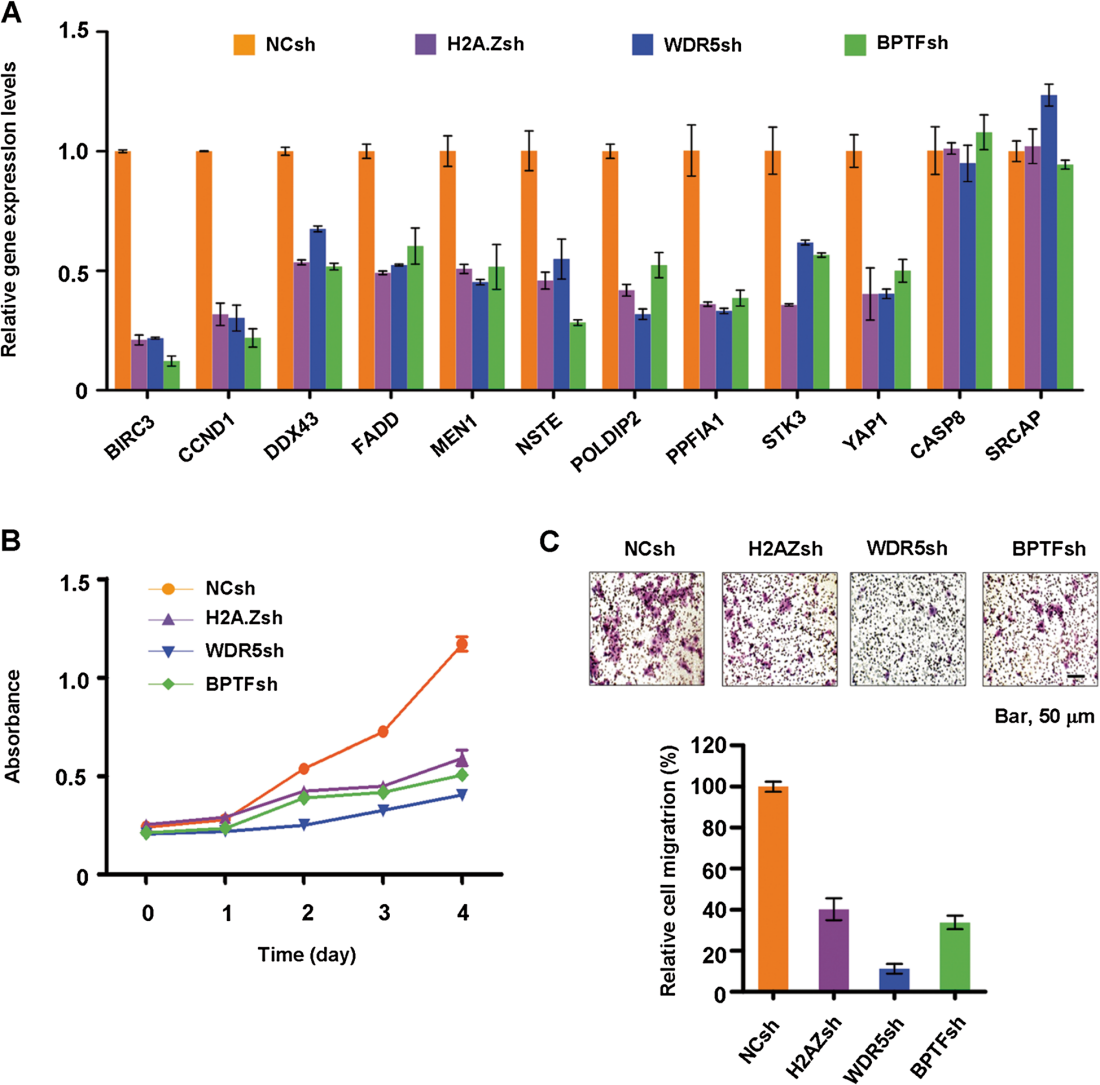
**Cooperative function of H2A.Z, WDR5 and BPTF. (A)** After silencing H2A.Z, WDR5 or BPTF in LD611 cells, the mRNA levels of the 10 genes, which show higher H2A.Z levels around the transcription start site (TSS) and augmented expression, were quantified by qRT-PCR. The values are expressed as fold changes from the levels in undepleted cells. **(B)** MTT proliferation assays were carried out in triplicate using LD611 cells depleted of H2A.Z, WDR5 or BPTF as indicated. **(C)** LD611 cells depleted of H2A.Z, WDR5 or BPTF were seeded onto the upper chamber, and then allowed to migrate toward 10% fetal bovine serum (FBS) in the lower chamber. The migrated cells were photographed and counted. The Y axis indicates the relative percentage of the migrated cell. Three independent experiments in triplicate wells were performed. MTT assay, 3–4, 5-(dimethyl-thyazol-2-yl)-2, 5-diphenyltetrazolium assay.

The fact that WDR5 and BPTF co-purified with H2A.Z nucleosomes raised the question of whether they play a potential role in regulating H2A.Z target genes. To address this question, we individually knocked down H2A.Z, WDR5, and BPTF in LD611 cancer cells and looked at the expression of H2A.Z target genes (Additional file 1: Figure S2). As expected, qRT-PCR analysis showed that knockdown of endogenous H2A.Z inactivated the 10 representative target genes (Figure 5A). These genes were also expressed at much lower levels in cells depleted of either WDR5 or BPTF as compared to control cells, indicating that WDR5 and BPTF are critical for transcriptional activation of the target genes. The requirements for WDR5 and BPTF were highly specific, as the two control genes, *CASP8* and *SRCAP*, were minimally affected by silencing endogenous WDR5 and BPTF.

Because WDR5 and BPTF are necessary for H2A.Z to upregulate gene transcription in the bladder cancer cells, we also check whether they are functionally linked to H2A.Z-induced cell growth. As summarized in Figure 5B, when H2A.Z was depleted in the LD611 cancer cells, the growth of the cancer cells was significantly inhibited over a four-day period. More importantly, knockdown of WDR5 or BPTF had the same inhibitory effect as knockdown of H2A.Z. In accordance with these results, individual knockdown of H2A.Z, WDR5 and BPTF also reduced cell migration severely, displaying about 70% lower migration than control cells (Figure 5C). Considered together, these findings demonstrate that H2A.Z functions cooperatively with WDR5 and BPTF to stimulate the expression of tumorigenic genes.

### H2A.Z is essential for H3K4me and WDR5/BPTF recruitment

**Figure 6:**
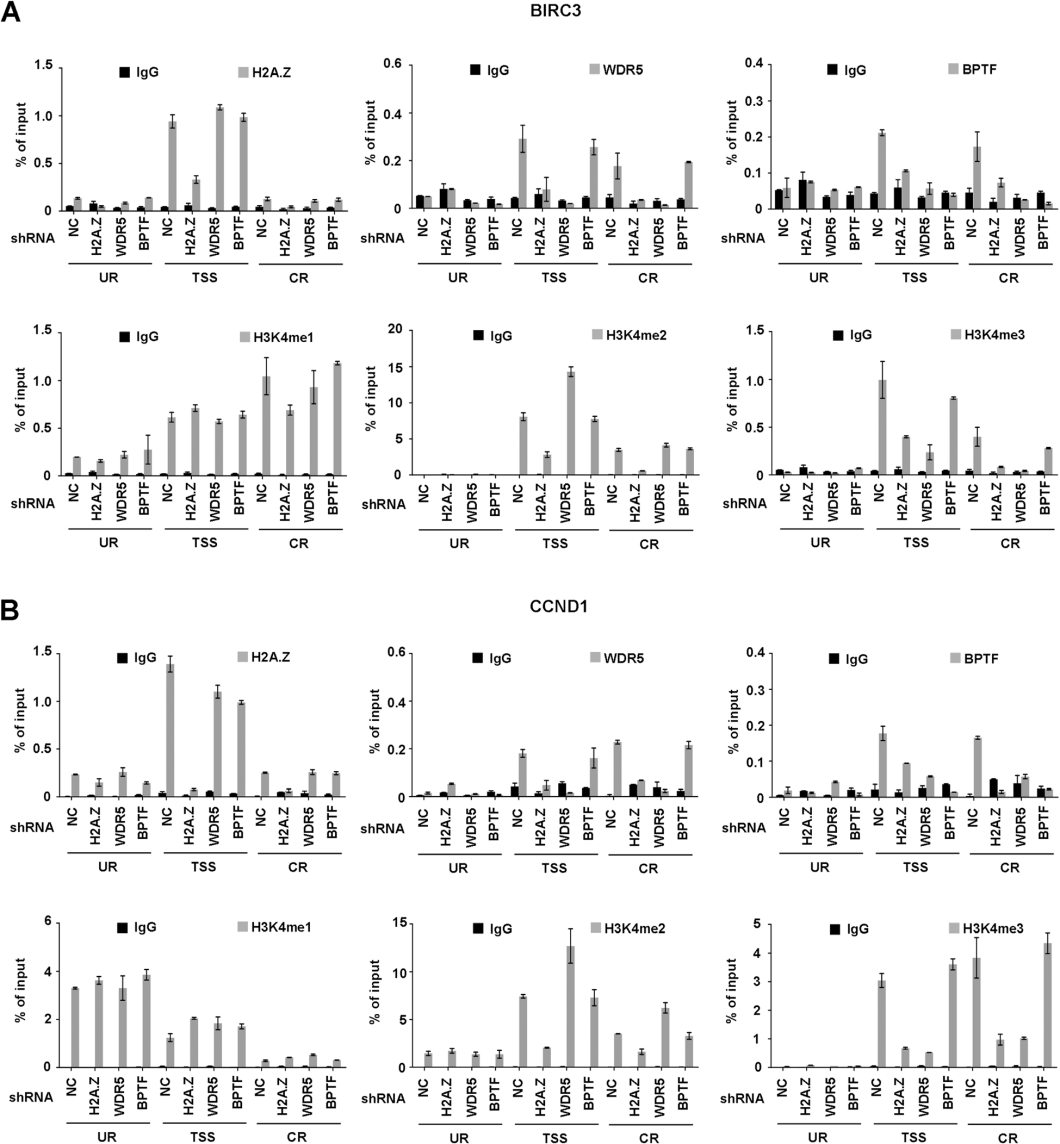
**H2A.Z-dependent recruitment of WDR5 and BPTF.** H2A.Z, BPTF and WDR5 were depleted from LD611 cells, and chromatin immunoprecipitation (ChIP) assays of *BIRC3***(A)** and *CCND1***(B)** genes were performed using antibodies against H2A.Z, WDR5, BPTF and H3K4me1/me2/me3. Precipitation efficiencies relative to nonenriched input samples were determined for upstream region (UR), transcription start site (TSS) and coding region (CR) by qPCR with primers listed in Additional file 6: Table S5. Percentage input is determined as the amount of immunoprecipitated DNA relative to input DNA.

To check whether the cooperative functions of WDR5 and BPTF in H2A.Z-activated genes reflect their targeted recruitment, we next investigated their localization at H2A.Z target genes by ChIP assays. Cross-linked chromatin was isolated from control cells and cells depleted of H2A.Z, WDR5 or BPTF, and the precipitated DNA was amplified by qPCR using primers specific for upstream region (UR), transcription start site (TSS) and coding region (CR). In agreement with the ChIP-seq data, we detected high H2A.Z signals around the TSS, but much reduced signals upstream and downstream of the TSS in *BIRC3* and *CCND1* genes (Figure 6A, B, H2A.Z). In determining regions occupied by WDR5 and BPTF, we found that their binding was near the TSS and coding region (WDR5 and BPTF). Consistent with our nucleosome purification studies and previous reports linking H3K4me2/me3 to the recruitment of WDR5 and BPTF [26-28], the distribution of H3K4me2/me3 at the loci was similar to those of WDR5 and BPTF (H3K4me2/me3).

These data raised the question of whether colocalization of WDR5 and BPTF at the target genes is dependent on H2A.Z-promoted H3K4me2/me3. In fact, the predicted effects of H2A.Z were confirmed by the observation that the levels of H3K4me2/me3, WDR5 and BPTF at the target loci were reduced in response to H2A.Z knockdown. On the contrary, knockdown of WDR5 or BPTF did not have substantial effects on H2A.Z occupancy around the TSS, placing WDR5 and BPTF in later steps of chromatin remodeling. Moreover, H3K4me3 and BPTF localization at the target genes decreased sharply upon WDR5 knockdown, suggesting that BPTF is recruited to the target genes via its physical recognition of WDR5-facilitated H3K4me3. Because the conversion of H3K4me2 to H3K4me3 is attenuated in WDR5-depleted cells, we detected a sharp increase in H3K4me2 over the TSS and CR of the target genes. As expected from the qRT-PCR results (Figure 5A), ChIP analysis showed no enrichments of H2A.Z, WDR5 and BPTF at the *CASP8* gene, which did not alter after individual depletion of H2A.Z, WDR5, and BPTF (Additional file 1: Figure S6). In view of the importance of H3K4me2/me3 for WDR5/BPTF recruitment, we also suspected that H2A.Z target genes may be selectively enriched for H3K4me2/me3, WDR5 and BPTF after ectopic H2A.Z expression in the normal cells. Indeed, when stably expressed in the UROtsa cells, ectopic H2A.Z produced a distinct increase in the levels of H3K4me2/me3 at the target genes and thereby facilitated the recruitments of WDR5 and BPTF (Additional file 1: Figures S7A and S7B). Consistent with these data, ectopic expression of H2A.Z in the normal cells increased target gene expression and cell migration (Additional file 1: Figures S7C and S7D). These results strongly argue that H2A.Z, WDR5 and BPTF function coordinately in regulating growth-controlling genes and their concerted actions are linked to H3K4me2/me3.

## Discussion

Despite significant efforts to understand altered expression and functional deregulation of H2A.Z in human cancer, we are still in the early stages of mechanistic studies for its contribution toward cancer development. Given the close correlation between H2A.Z exchange and gene activation in the context of chromatin, H2A.Z has been implicated in upregulating the network of genes during carcinogenesis. To gain deeper understanding of oncogenic effects of H2A.Z, we analyzed H2A.Z localization and function in bladder cancer cells by ChIP-seq and gene expression array. Our analysis of H2A.Z-enriched genes in bladder cancer cells revealed a significant overrepresentation for tumorigenic genes in general. By comparing transcriptional profiles, we found that approximately 28% of the H2A.Z-enriched genes are activated, whereas only approximately 5% of the genes are repressed, in the cancer cells. This is significant because H2A.Z is known to regulate transcription positively or negatively. However, the changes in the expression and localization of H2A.Z in bladder cancer cells mainly correlate with gene activation, with the most activated genes tending to be related to cell growth regulation. Thus, our data favor the mechanism in which the elevated expression and redistribution of H2A.Z contribute to selective, rather than general, disruption of transcription integrity in bladder carcinoma cells. The fact that H2A.Z knockdown affects the same sets of genes in cancer cells further confirms that H2A.Z is a critical driving force for the tumorigenic transcriptional output. This study represents the first genome-wide analysis of H2A.Z in bladder cancer cells, and establishes H2A.Z as a key component whose enrichment near the TSS drives expression of major cell cycle regulatory genes capable of facilitating cell growth and proliferation. Recent studies in human osteosarcoma (U2OS) cell line showed that H2A.Z is incorporated into the p21 promoter region in p53-dependent manner, and establishes a repressive barrier to p53-mediated p21 induction [29]. However, we did not observe any significant change in p21 transcription after H2A.Z knockdown in LD611 cells (data not shown). Furthermore, our ChIP-seq data indicate that H2A.Z is incorporated into p21 promoter nucleosomes at low levels in both LD611 and UROtsa cells. These results imply that H2A.Z-catalyzed oncogenic pathway in bladder cancer cells is minimally dependent on a negative behavior of H2A.Z toward p21 gene.

Although the mechanism by which H2A.Z promotes chromatin transcription remains unclear, our interaction data demonstrated that the presence of H2A.Z in the nucleosome increases the levels of H3K4me2 and H3K4me3, marks that are associated with gene activation. These results are in good agreement with those of recent studies showing that H2A.Z colocalizes with active histone modifications in transcription regulatory regions [7,10,30-32]. It is also conceivable that H2A.Z-induced H3K4me2/me3 at the TSS coincides with the recruitment of WDR5 and BPTF, which are essential components of the MLL H3K4-methyltransferase complex and the NURD ATP-dependent remodeling complex, respectively [33-35]. This suggests that H2A.Z is an integral part of histone modification-mediated factor recruitment processes and has a direct role in resetting transcription competency of growth-regulatory genes in bladder cancer. A recent study reported copurification of WDR5 with H2A.Z nucleosomes, but BPTF was absent in the purified H2A.Z nucleosomes [30]. Clearly, these differences could be due to different purification approaches (for example, single-step purification using the Flag-tag moiety in the previous study versus two-step purification using Flag and HA tags in the current study) and different wash conditions (150 mM NaCl versus 300 mM NaCl). We speculate that the weak binding of BPTF to H2A nucleosomes is not preserved under our stringent purification conditions. It is also noteworthy that H2A.Z nucleosomes isolated from bladder cancer cells display the levels of the two repressive histone marks, H3K9me3 and H3K27me3, similar to those of H2A nucleosomes. Thus, although further work will be required to gain a complete understanding of how H2A.Z functionally interacts with various histone modifications, these findings nevertheless illustrate that H2A.Z overexpression in bladder cancer cells is likely to act in concert with active histone modifications which establish constitutive expression of the target genes.

## Conclusions

We show that H2A.Z is overexpressed in bladder cancer cells and establishes the active states of oncogenic transcription. H2A.Z enrichments at target genes increase the levels of H3K4me2 and H3K4me3, which in turn facilitate the recruitments of WDR5 and BPTF. These results unveil a specific contribution of H2A.Z to chromatin remodeling in bladder cancer cells and emphasize the importance of accurate levels of H2A.Z expression to prevent the onset of tumorigenesis.

## Methods

### Cell culture, constructs and antibodies

LD611 cells were maintained in Dulbecco’s modified Eagle’s medium (DMEM) supplemented with 10% fetal bovine serum (FBS). UROtsa cells were cultured in DMEM containing 2 mg/ml glucose and 5% FBS. For mammalian expression of H2A and H2A.Z, their cDNAs were amplified by PCR and ligated into the NheI and BamHI sites of pIRES expression vector containing 5′ Flag and HA coding sequences. Antibodies used in this study are as follows: H2A, H2A.Z, H3K4me1 and WDR5 antibodies from Abcam (Cambridge, UK), Actin, HA and Flag antibodies from Sigma-Aldrich (St Louis, MO, USA), BPTF antibody from Bethyl Laboratories (Montgomery, TX, USA), H3K4me3 and H3K9me3 antibodies from Active Motif (Carlsbad, CA, USA), and H3K27me2 and H3K27me3 antibodies from Merck Millipore (Darmstadt, Germany).

### Tissue microarrays and immunohistochemistry

The expression patterns of H2A.Z were determined in human bladder tissue microarrays, which include 16 cases of bladder malignant tissues and eight cases of normal bladder tissues (US Biomax, Rockville, MA, USA). The tissues were deparaffinized, treated with 3% hydrogen peroxide to block endogenous peroxide, and subjected to antigen retrieval with citrate buffer (10 mM, pH 6.0). The tissue sections were blocked in 5% normal goat serum in Tris-buffered saline-Triton X-100 (TBST, 50 mM Tris–HCl, pH 7.5, 150 mM NaCl, and 0.3% Triton X-100) for 30 min at room temperature and incubated with H2A.Z antibody at 4°C overnight. Immunodetection was performed using Vectastain ABC kit (Vector Laboratories, Burlingame, CA, USA) according to manufacturer’s recommendations. DAB (Vector Laboratories) and hematoxylin (Sigma-Aldrich) were used for color development and counterstaining. The staining intensity was evaluated by semiquantitative immunohistochemical assessment, and divided into four categories: negative staining (less than 20% of cells), weak staining (21 to 50% of cells), moderate staining (51 to 80% of cells) and strong staining (more than 80% of cells).

### ChIP-seq

H2A.Z ChIP for UROtsa normal and LD611 bladder cancer cell lines (1 × 10^8^ cells) was carried out following a standard protocol as described previously [36]. Briefly, cells were fixed for 10 min in 1% formaldehyde, and chromatin was sheared with a bioruptor (Diagenode, Denville, NJ, USA) to an average size of 200 to 500 bp. Lysates were clarified by centrifugation at 20,000 × g for 15 min at 4°C, and incubated with anti-H2A.Z antibody overnight. Protein A/G agarose beads were added to protein complexes for 60 min at 4°C. Immunoprecipitates were washed three times in wash buffer (20 mM Tris–HCl, pH 8.0, 300 mM NaCl, 2 mM EDTA, 0.1% SDS, and 1% Triton X-100), once in LiCl buffer (10 mM Tris–HCl, pH 8.0, 1% deoxycholate, 1 mM EDTA, 0.25 M LiCl, and 1% NP-40), twice in TE buffer (10 mM Tris–HCl, pH 8.0 and 1 mM EDTA) and then eluted in elution buffer (1% SDS and 0.1 M NaHCO_3_). Crosslinking was reversed by overnight incubation at 65°C. After RNase A (200 μg/ml) and proteinase K (200 μg/ml) digestions, ChIP DNA was purified by phenol-chloroform extraction and ethanol precipitation. The high-throughput DNA sequencing was performed using Illumina HiSeq 2000 (Illumina, San Diego, CA, USA) at the University of Southern California (USC) Genomic Core.

### ChIP-seq data analysis

Approximately 17 and 13 million sequences were generated for the normal and cancer cells, respectively. MAQ was used to generate 14 and 11 million aligned reads to hg18 reference genome (mapping quality score ≥20) [37]. One of the duplicated sequences was kept to minimize the artifacts of PCR amplification. Since we used single-end sequencing, each read was extended in the sequencing orientation to a total of 250 bases to infer the coverage at each genomic position. The genome was divided into non-overlapping windows, and aligned read was considered to be within a window of the midpoint of its estimated fragment. Mid-points in each window were counted, and empirical distributions of windows counts were created. The Java-based EpiChIP software was used to identify optimal sequence window with respect to gene coordinates for analysis of H2A.Z enrichment at all Reference Sequence (RefSeq) genes. In this analysis, normalized ChIP-seq signal for each gene (area below the peak and within window) was quantified to yield NLCS (normalized locus-specific chromatin state) value by using expectation and maximization algorithm and a curve fitting model of Baysen information criteria as previously described [24,38]. The mapping output files were also converted to browser-extensible data (BED) files. For visualization, wiggle tracks were generated by computing mean read density over 25 bp bins of human genome with aligned and filtered reads from UROtsa and LD611 ChIP-seq data. Wiggle tracks were visualized in the IGV (Integrated Genomic Viewer) [39]. K-means clustering was performed on ChIP-seq tag density around 3 kb regions across the TSS in R statistical software (http://www.r-project.org).

### Gene expression microarray

Total RNA was isolated from LD611 and UROtsa cells using the TRIzol reagent (Invitrogen, Carlsbad, CA, USA). Gene expression microarray experiments were conducted using a whole-genome expression array (Human HT-12 v4 Expression BeadChip; Illumina). This high-density oligonucleotide array chip targets more than 47,000 probe sequences derived from National Center for Biotechnology Information Reference Sequence (NCBI) RefSeq Release 38 (November 7, 2009) and other sources. Data were background-corrected using Genome Studio (Illumina) and were quantile-normalized. Genes showing detection *P* <0.05 with fold change >1.5 were selected as suggested by the manufacturer (Illumina). Genes which are differentially associated with H2A.Z in LD611 cancer cells were functionally analyzed in the context of gene ontology and molecular networks by using Ingenuity pathway software (IPA; http://www.ingenuity.com). Differentially regulated genes were categorized into various functional groups (threshold *P* <0.05) and mapped to genetic networks.

### RNA interference, qRT-PCR, and ChIP

For shRNA-based knockdown, DNA oligonucleotides encoding shRNAs specific for H2A.Z mRNA (5′-GCTTCAAAGAAGCTATTGATT-3′), WDR5 (5′-GAGAGTGGCTGGCAAGTTCAT-3′) and BPTF mRNA (5′-CGCCACTAACAGAGAAGGATT-3′) were annealed and ligated into the lentiviral expression vector pLKO.1 (Addgene, Cambridge, MA, USA). Lentivirus particles were generated in 293T cells by co-transfecting plasmids encoding VSV-G, NL-BH and the shRNAs. For H2A.Z, WDR5 and BPTF knockdown, LD611 cells were infected with these viruses and selected with puromycin (2 μg/ml) for two weeks. For qRT-PCR, total RNA (2 μg) was reverse transcribed using the iScript cDNA Synthesis Kit (Bio-Rad Laboratories, Hercules, CA, USA) and the PerfeCta™ SYBR Green FastMix (Quanta BioSciences Gaithersburg, MD, USA) as described [36]. All samples were run in triplicate, and results were averaged. Sequences of the primers used for qRT-PCR are listed in Additional file 6: Table S5. For conventional ChIP assays, LD611 cells were harvested, fixed with 1% formaldehyde and processed for immunoprecipitation using antibodies against H2A.Z, WDR5, BPTF, and H3K4me1/me2/me3 as recently described [36]. qPCR was performed in triplicate using the primers listed in Additional file 6: Table S5.

### Nucleosome purification and protein identification

Nuclei were prepared from LD611 cells expressing Flag and HA-tagged H2A or H2A.Z and were digested with micrococcal nuclease to produce predominantly mononucleosomes. Mononucleosomes containing ectopic H2A or H2A.Z were subjected to anti-Flag M2 agarose. After extensive washing with wash buffer (20 mM HEPES-KOH, pH 7.9, 0.5 mM EDTA, 300 mM NaCl, 1 mM dithiothreitol, 10% glycerol, and 0.1% Nonidet P-40), the eluates were further purified using anti-HA agarose affinity chromatography. The imunoprecipitated nucleosome samples were analyzed by liquid chromatography-tandem mass spectrometry (LC-MS/MS) as described [40].

### Cell proliferation and migration assays

Cell proliferation was assessed by the 3–4, 5-(dimethyl-thyazol-2-yl)-2, 5-diphenyltetrazolium (MTT) assay as previously described [36]. In brief, cells were seeded in 24-well tissue culture plates at a density of 2 × 10^4^ and treated with the MTT labeling reagent (0.5 mg/ml) at 37°C for 1 h. The blue MTT formazan precipitate was dissolved with the MTT solvent (0.2 ml) and measured at a wavelength of 570 nm using a microplate reader (Bio-Rad). To measure the cell migration activity, Transwell migration assays were performed using 5 × 10^4^ cells. Cells suspended in serum-free medium were plated on the Transwell filters (8-μm pore size; Corning, Corning, NY, USA), and DMEM supplemented with 10% FBS was used as chemoattractant. Cells were stained with 0.5% crystal violet and quantified after migration for 18 h.

## Competing interests

The authors declare that they have no competing interests.

## Authors’ contributions

KK and WA conceived and designed the experiments. VP performed computational analyses. KK, JC, KH and JK carried out the experiments. PL provided reagents and technical assistance. KK, VP and WA wrote the manuscript. All authors read and approved the final manuscript.

## Supplementary Material

Additional file 1 Gene dysregulation by histone variant H2A.Z in bladder cancer. Click here for file

Additional file 2: Table S1 List of H2A.Z enriched genes by ChIP-seq. Click here for file

Additional file 3: Table S2 Differential gene expression in cancer vs. normal cells. Click here for file

Additional file 4: Table S3 Genes overlapping with ChIP-seq and gene expression array. Click here for file

Additional file 5: Table S4 H2A and H2A.Z nucleosomes interacting proteins. Click here for file

Additional file 6: Table S5 Primer sequences for RT-PCR and ChIP. Click here for file
