# Supplementary material for: Gene dysregulation by histone variant H2A.Z in bladder cancer
Source: Epigenetics Chromatin. 2013 Oct 16;6:34. doi: 10.1186/1756-8935-6-34 (PMC3853418; doi:10.1186/1756-8935-6-34)
Supplement: Additional file 1 — Gene dysregulation by histone variant H2A.Z in bladder cancer. [file 1756-8935-6-34-S1.pdf]

Gene dysregulation by histone variant H2A.Z in bladder cancer

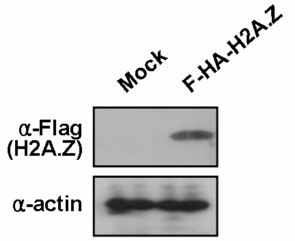

Figure S1. Ectopic expression of H2A.Z in normal bladder cells.

UROtsa cells were transfected with mock and Flag-HA-H2A.Z expression vectors, and the levels of H2A.Z were analyzed by Western blotting using anti-Flag antibody.

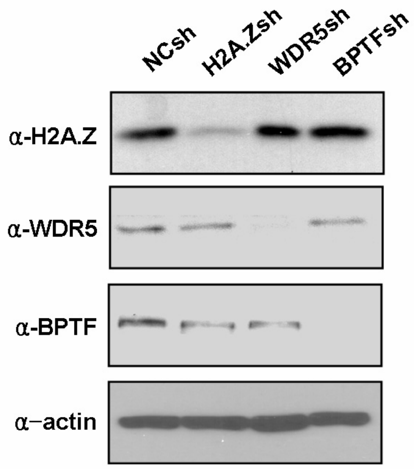

Figure S2. Stable depletions of H2A.Z, WDR5 and BPTF in bladder cancer cells.

LD611 cells were stably infected with lentiviruses expressing either control shRNA (NC) or a shRNA specific to H2A.Z, WDR5 or BPTF. The efficiency and selectivity of depletion were determined by Western blotting using the indicated antibodies.

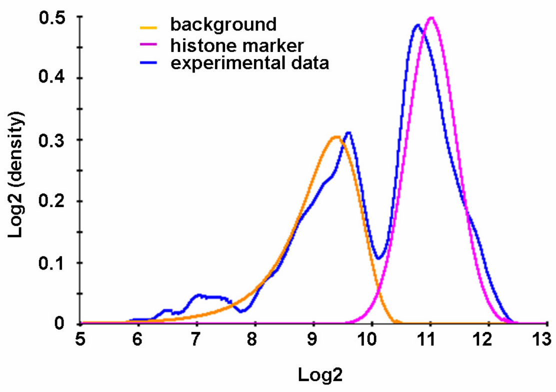

Figure S3. Curve fitting of cancer specific ChIP-seq data.

The experimental data is shown in blue, background (BG) curve in orange and histone marker (HM) curve in purple.

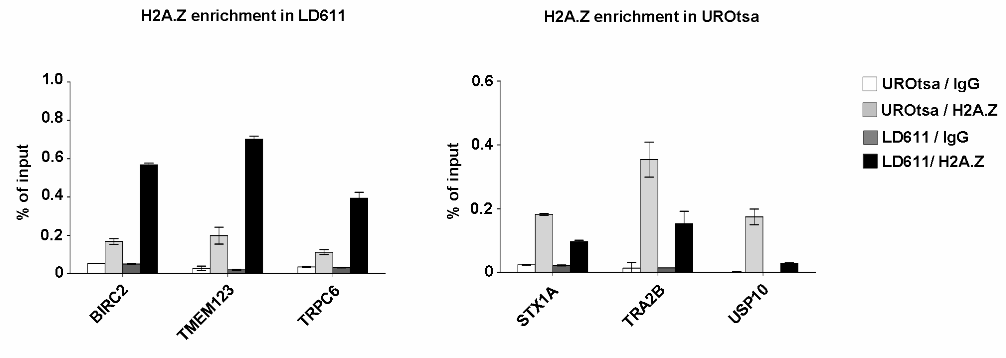

Figure S4. Validation of ChIP-seq analysis.

ChIP experiments were performed using anti-H2A.Z antibody to validate ChIP-seq data. Precipitation efficiencies relative to non-enriched input samples were determined by qPCR with primers listed in Additional file 6: Table S5.

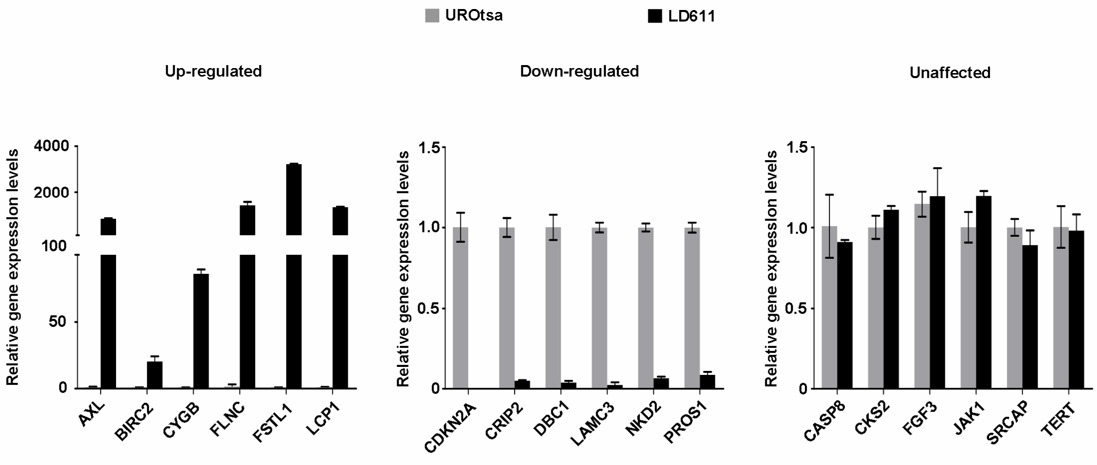

Figure S5. Validaton of gene expression array analysis.

The mRNA levels of three groups of genes whose expressions were upregulated, down-regulated and unaffected in the LD611 cancer cells were validated by qRT-PCR, and the values are expressed as fold changes from the levels in UROtsa normal cells.

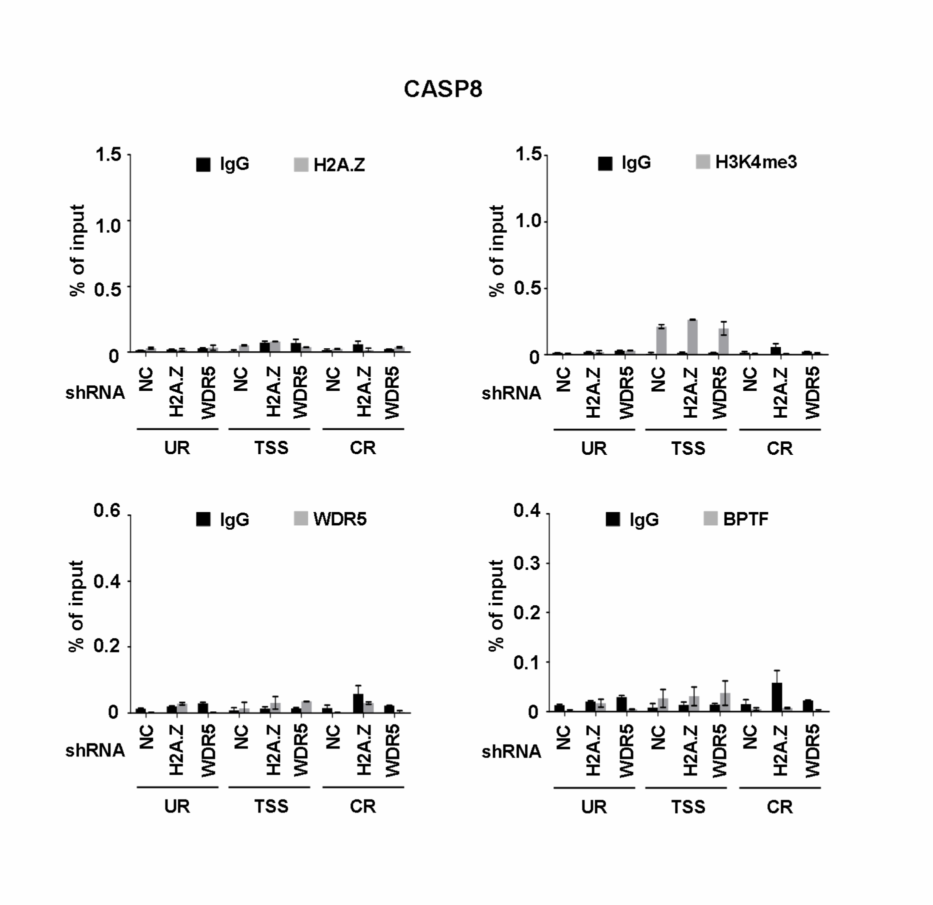

Figure S6. ChIP analysis of control gene in bladder cancer cells.

ChIP assays of control gene, *CASP8*, were performed as in Figure 5B using the indicated antibodies. The precipitated DNA was quantified by qPCR with the primers listed in Additional file 6: Table S5.

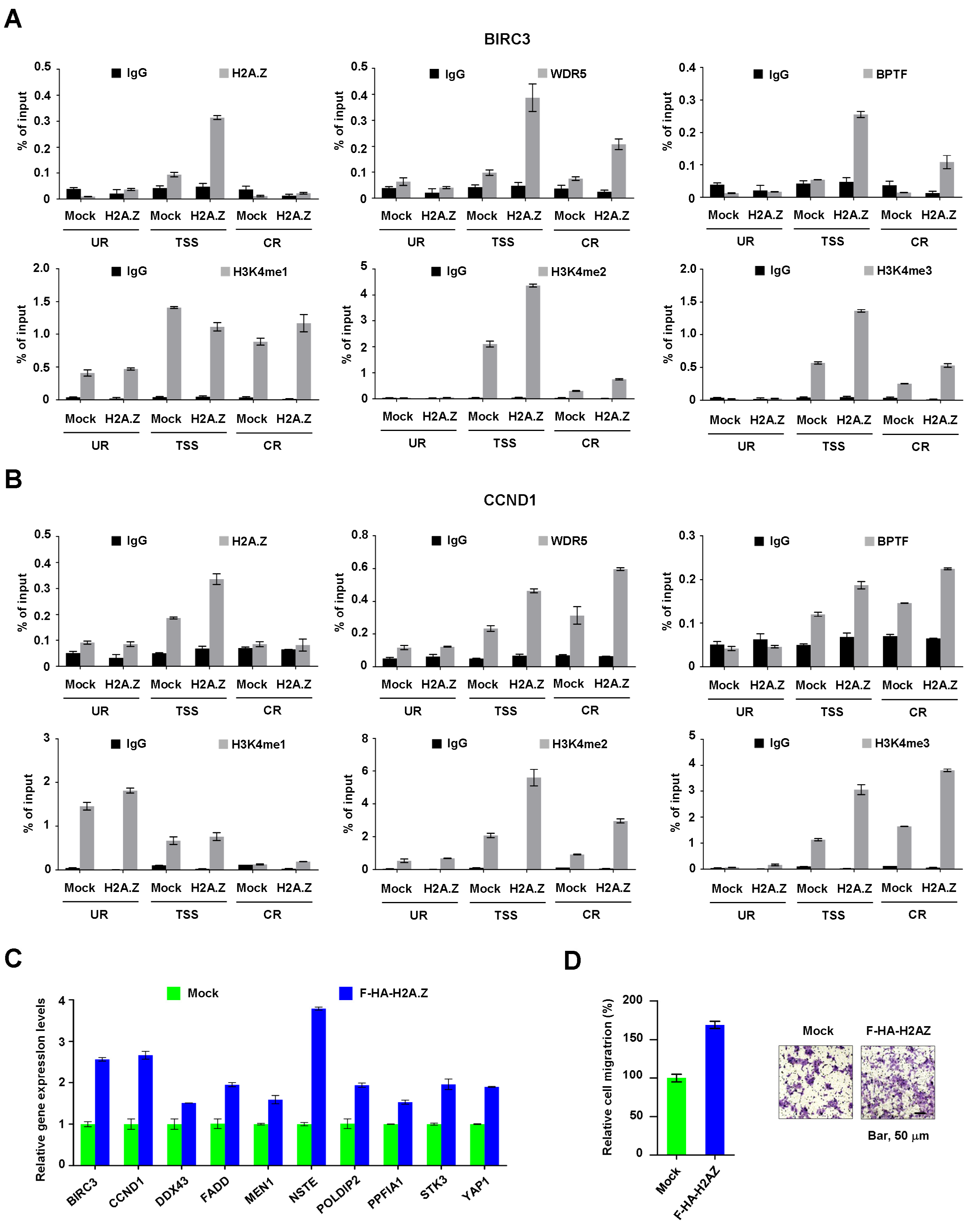
A
BIRC3
IgG
H2A.Z
WDR5
BPTF
H3K4me1
H3K4me2
H3K4me3
% of input
Mock
UR
TSS
CR
B
CCND1
C
Mock
F-HA-H2A.Z
Relative gene expression levels
BIRC3
CCND1
DDX43
FADD
MEN1
NSTE
POLDIP2
PPFIA1
STK3
YAP1
D
Relative cell migratrion (%)
Mock
F-HA-H2AZ
Bar, 50 μm

Figure S7. Effects of ectopic H2A.Z on H3Kme and WDR5/BPTF recruitment in normal bladder cells.

(**A** and **B**) Normal bladder UROtsa cells were stably transfected with mock or Flag-HA-H2A.Z expression construct as in Figure S1, and ChIP assays were performed on BIRC3 and CCND1 genes (**C**) RNA was isolated from normal bladder UROtsa cells stably expressing mock or Flag-HA-H2A.Z and subjected to qRT-PCR using primers specific for the indicated genes (**D**) Migratin assays were carried out using UROtsa cells expressing mock or Flag-HA-H2A.Z as in Figure 5C.
