# Supplementary material for: Gene dysregulation by histone variant H2A.Z in bladder cancer
Source: Epigenetics Chromatin. 2013 Oct 16;6:34. doi: 10.1186/1756-8935-6-34 (PMC3853418; doi:10.1186/1756-8935-6-34)
Supplement: Additional file 2: Table S1 — List of H2A.Z enriched genes by ChIP-seq. [file 1756-8935-6-34-S2.pdf]

Supplementary Table S1. List of H2A.Z enriched genes by ChIP-seq.

Group 1: High confidence ChIP-seq identfied H2.AZ regulated genes with NLCS cutoff ≥8.5; FDR for noise (BG) and histone mark separation ≤0.01			
Gene	Chromome	Cancer_NLCS (Normalized locus-specific chromatin state)	Probability to be signal
FAM69A	chr1	10.15390538	1
CCND1	chr11	10.15390538	1
TRPC6	chr11	10.15390538	1
YAP1	chr11	10.15390538	1
BIRC3	chr11	10.15390538	1
BIRC2	chr11	10.15390538	1
TMEM123	chr11	10.15390538	1
FADD	chr11	10.12475978	1
PAG1	chr8	10.08293938	1
MMP7	chr11	10.07076498	1
FAM82B	chr8	10.05601716	1
ZNF704	chr8	10.0523072	1
ORAOV1	chr11	10.02606414	1
PPFIA1	chr11	9.985134644	1
TMEM67	chr8	9.968168531	1
SNX16	chr8	9.952329198	1
ANO1	chr11	9.914679621	1
DHCR7	chr11	9.881608034	1
TMEM97	chr17	9.874621781	1
C17orf79	chr17	9.863373239	1
LRRIQ4	chr3	9.856297906	1
NEK11	chr3	9.840608	1
SSPN	chr12	9.837736722	1
GPR137	chr11	9.804302069	1
NEK5	chr13	9.801357543	1
SH3BGRL2	chr6	9.798408616	1
ADAP2	chr17	9.764038899	1
MEN1	chr11	9.748836886	1
C20orf177	chr20	9.738099017	1
SETDB2	chr13	9.730380125	1
EXOSC8	chr13	9.722618005	1
ESD	chr13	9.702243693	1
SPG20	chr13	9.684774915	1
ASCC3	chr6	9.66060922	1
TAF6L	chr11	9.655727482	1
ZNF615	chr19	9.645912368	1
ZFAND1	chr8	9.640980559	1
ASTE1	chr3	9.619409342	1
MUS81	chr11	9.589001272	1
FNTB	chr14	9.589001272	1
MACROD2	chr20	9.585582276	1

POLDIP2	chr17	9.580439482	1
RRM2B	chr8	9.578719878	1
ATF2	chr2	9.57009667	1
CBLL1	chr7	9.559681086	1
C20orf30	chr20	9.557936545	1
TPD52	chr8	9.556191808	1
ELMO2	chr20	9.550941055	1
PEX2	chr8	9.538617699	1
MRPL11	chr11	9.535078986	1
DHRS13	chr17	9.531529622	1
SSRP1	chr11	9.520828833	1
SLC35B4	chr7	9.515449513	1
TUBBP5	chr9	9.513652598	1
C1orf25	chr1	9.506438504	1
UTP6	chr17	9.504628337	1
C10orf25	chr10	9.499188155	1
LIN7C	chr11	9.495549271	1
ATXN3	chr14	9.48824586	1
RAE1	chr20	9.48824586	1
BBS1	chr11	9.471673182	1
CCDC34	chr11	9.46051733	1
LOC645332	chr11	9.451155464	1
KCNS1	chr20	9.449276604	1
ARID4A	chr14	9.443621155	1
OTUB1	chr11	9.436049374	1
GPR85	chr7	9.434148639	1
LGMN	chr14	9.430341732	1
CAB39L	chr13	9.426526848	1
C19orf2	chr19	9.426526848	1
TMEM209	chr7	9.424613518	1
WWP1	chr8	9.422699749	1
MTRF1	chr13	9.41886247	1
BDNFOS	chr11	9.415014957	1
ZNF85	chr19	9.415014957	1
C7orf55	chr7	9.409225441	1
DNAJC28	chr21	9.403412598	1
PLEKHH1	chr14	9.401468345	1
ERLIN2	chr8	9.397574101	1
TMEM199	chr17	9.391714027	1
GEM	chr8	9.389756084	1
CDC73	chr1	9.385830053	1
FOXN3	chr14	9.385830053	1
ARL11	chr13	9.379919818	1
DCAF11	chr14	9.352023557	1
WDR19	chr4	9.352023557	1
TRPT1	chr11	9.350008752	1
HNRNPA1L2	chr13	9.34395184	1

AHCYL2	chr7	9.34395184	1
PM20D1	chr1	9.341925723	1
TLCD1	chr17	9.341925723	1
C6orf136	chr6	9.341925723	1
RNGTT	chr6	9.341925723	1
MUDENG	chr14	9.337869391	1
ZC3H13	chr13	9.335834708	1
CRY2	chr11	9.323574983	1
TTC9C	chr11	9.317403569	1
C6orf57	chr6	9.313275333	1
C8orf38	chr8	9.309137525	1
KIAA0100	chr17	9.290359891	1
RBM12B	chr8	9.288257038	1
ENTPD5	chr14	9.281934662	1
TMEM86A	chr11	9.267072241	1
RECQL	chr12	9.264937489	1
FGF19	chr11	9.262797225	1
C6orf211	chr6	9.262797225	1
MIR632	chr17	9.260656133	1
CNO	chr4	9.258509503	1
PTK2B	chr8	9.256362033	1
IFT20	chr17	9.254211362	1
PTGS2	chr1	9.249898009	1
FLJ10661	chr8	9.247735299	1
KIAA0146	chr8	9.241232419	1
NDUFA5	chr7	9.236880028	1
IFRD1	chr7	9.232514465	1
NUDT22	chr11	9.230327922	1
RAD9A	chr11	9.228138059	1
BSCL2	chr11	9.221543622	1
KBTBD6	chr13	9.212707941	1
KIAA1429	chr8	9.212707941	1
SLC25A19	chr17	9.201582405	1
ZNF207	chr17	9.197111006	1
SYVN1	chr11	9.194867637	1
THAP8	chr19	9.190372876	1
KDM1B	chr6	9.185866545	1
WDHD1	chr14	9.176806449	1
RIC3	chr11	9.174531259	1
ILK	chr11	9.172254975	1
FAM185A	chr7	9.172254975	1
ASRGL1	chr11	9.158513758	1
HCP5	chr6	9.144642949	1
CPNE3	chr8	9.144642949	1
SPTY2D1	chr11	9.135319385	1
GPD2	chr2	9.135319385	1
RAB1B	chr11	9.128288245	1

ZSCAN12	chr6	9.128288245	1
ZNRD1	chr6	9.123580846	1
UNC119	chr17	9.116492122	1
KIF24	chr9	9.116492122	1
GJC1	chr17	9.111746012	0.9999
PMPCB	chr7	9.109368396	0.9999
SPAG8	chr9	9.109368396	0.9999
PNPLA2	chr11	9.104598754	0.9999
BRMS1	chr11	9.09981329	0.9999
SLC46A3	chr13	9.09981329	0.9999
STX18	chr4	9.085360917	0.9999
PROCA1	chr17	9.080511106	0.9999
C11orf17	chr11	9.078081407	0.9999
NDUFS8	chr11	9.073207022	0.9999
SLC3A2	chr11	9.070764981	0.9999
NUPL1	chr13	9.065865771	0.9999
TUBD1	chr17	9.065865771	0.9999
TASP1	chr20	9.065865771	0.9999
QTRTD1	chr3	9.065865771	0.9999
FBXO24	chr7	9.065865771	0.9999
COX16	chr14	9.063408564	0.9999
ANKRD5	chr20	9.063408564	0.9999
DCLRE1C	chr10	9.058486972	0.9998
ZNF649	chr19	9.058486972	0.9998
TPK1	chr7	9.056017155	0.9998
USP8	chr15	9.05106752	0.9998
TMEM143	chr19	9.05106752	0.9998
HM13	chr20	9.048587682	0.9998
KPNA3	chr13	9.046103574	0.9998
RSAD1	chr17	9.041119751	0.9998
CHCHD5	chr2	9.041119751	0.9998
MYO5A	chr15	9.038619997	0.9998
SSR1	chr6	9.038619997	0.9998
C6orf203	chr6	9.038619997	0.9998
HSF2	chr6	9.036118652	0.9998
GOPC	chr6	9.033610209	0.9998
MPHOSPH6	chr16	9.031100156	0.9997
ANKRD13B	chr17	9.028585727	0.9997
RNF32	chr7	9.026064141	0.9997
TRIM21	chr11	9.018478396	0.9997
IMPAD1	chr8	9.018478396	0.9997
ALG5	chr13	9.015939067	0.9997
HOXB13	chr17	9.015939067	0.9997
TFB1M	chr6	9.015939067	0.9997
MIR933	chr2	9.013398052	0.9996
NBN	chr8	9.010852553	0.9996
GTF2H1	chr11	9.003183376	0.9996

ZNF596	chr8	9.003183376	0.9996
CTR9	chr11	9.000619775	0.9996
C8orf39	chr8	8.998048789	0.9995
FAM86C	chr11	8.995476039	0.9995
ACTR10	chr14	8.992898693	0.9995
ELOVL4	chr6	8.990313898	0.9995
PPP2R5C	chr14	8.982537314	0.9994
MED28	chr4	8.982537314	0.9994
RGS2	chr1	8.974718585	0.9993
ZNF22	chr10	8.974718585	0.9993
LOC730101	chr6	8.969481499	0.9993
MLST8	chr16	8.964225332	0.9992
TRMT6	chr20	8.964225332	0.9992
MADD	chr11	8.958949946	0.9991
YIF1A	chr11	8.958949946	0.9991
PIP4K2A	chr10	8.953655198	0.999
C14orf159	chr14	8.953655198	0.999
TRIM3	chr11	8.945677926	0.9989
ZDHHC24	chr11	8.945677926	0.9989
TRUB1	chr10	8.934970516	0.9987
DNAL1	chr14	8.932282677	0.9986
SFRS18	chr6	8.932282677	0.9986
C20orf134	chr20	8.929586862	0.9986
BCL2L10	chr15	8.926888964	0.9985
VPS37A	chr8	8.921475011	0.9984
TMX2	chr11	8.913317292	0.9982
COX8A	chr11	8.913317292	0.9982
PDS5B	chr13	8.907852072	0.998
WDR74	chr11	8.905113183	0.9979
ATP5C1	chr10	8.899616736	0.9977
AURKA	chr20	8.899616736	0.9977
NFX1	chr9	8.899616736	0.9977
C20orf94	chr20	8.896859126	0.9976
GSTK1	chr7	8.896859126	0.9976
RSU1	chr10	8.894099268	0.9975
NAA20	chr20	8.888560618	0.9973
TIMM10	chr11	8.880214099	0.997
TSPYL1	chr6	8.877419116	0.9968
ZNF682	chr19	8.866193994	0.9963
CLUL1	chr18	8.860546958	0.996
SERINC1	chr6	8.857713574	0.9958
KIN	chr10	8.854877731	0.9956
ATOH7	chr10	8.854877731	0.9956
ACPP	chr3	8.854877731	0.9956
DIDO1	chr20	8.852036303	0.9955
PTER	chr10	8.849186139	0.9953
ATXN7L1	chr7	8.849186139	0.9953

SF3B2	chr11	8.834859719	0.9943
C14orf37	chr14	8.829088444	0.9938
PIGU	chr20	8.826195715	0.9935
NT5DC1	chr6	8.823293989	0.9933
ATP5E	chr20	8.820389606	0.993
DCTN6	chr8	8.820389606	0.993
C9orf46	chr9	8.817479365	0.9927
DDB1	chr11	8.814560036	0.9924
MTCH2	chr11	8.808706815	0.9918
PCDHB5	chr5	8.808706815	0.9918
HLA-F	chr6	8.808706815	0.9918
MRPL49	chr11	8.805772887	0.9915
RCE1	chr11	8.805772887	0.9915
C14orf142	chr14	8.805772887	0.9915
RNMT	chr18	8.80282975	0.9912
CBWD2	chr2	8.80282975	0.9912
RALGAPB	chr20	8.799883833	0.9908
ISCA2	chr14	8.796931889	0.9905
NPHP1	chr2	8.796931889	0.9905
KY	chr3	8.793970642	0.9901
ZNF429	chr19	8.791006561	0.9897
SMC1A	chrx	8.791006561	0.9897
BEND6	chr6	8.788033114	0.9893
MATN2	chr8	8.788033114	0.9893
SMPD1	chr11	8.785056797	0.9889
CDCA5	chr11	8.785056797	0.9889
TRIM13	chr13	8.785056797	0.9889
VPS36	chr13	8.785056797	0.9889
NDUFB9	chr8	8.782071049	0.9885
ANTXR1	chr2	8.779082394	0.9881
ORC5L	chr7	8.776087534	0.9876
PRPF18	chr10	8.773083147	0.9871
LOC729234	chr2	8.773083147	0.9871
KIAA1797	chr9	8.773083147	0.9871
PGAP2	chr11	8.767058848	0.9862
ZSWIM1	chr20	8.764038899	0.9856
PCDHB10	chr5	8.757976636	0.9845
C10orf67	chr10	8.754937595	0.984
KLRG2	chr7	8.751888792	0.9834
MRPS31	chr13	8.745775149	0.9821
DEAF1	chr11	8.742710269	0.9814
CHURC1	chr14	8.742710269	0.9814
TOMM40L	chr1	8.739638864	0.9808
SRD5A3	chr4	8.739638864	0.9808
PCDHB9	chr5	8.739638864	0.9808
CLP1	chr11	8.736557524	0.9801
CSE1L	chr20	8.736557524	0.9801

C3orf38	chr3	8.733472978	0.9793
FAM66D	chr8	8.733472978	0.9793
ZNF233	chr19	8.730378427	0.9786
LRGUK	chr7	8.730378427	0.9786
COG3	chr13	8.727280627	0.9778
SNX11	chr17	8.727280627	0.9778
SDHAF2	chr11	8.72417275	0.977
ABCA11P	chr4	8.72417275	0.977
MED19	chr11	8.721061582	0.9762
RINT1	chr7	8.717943691	0.9753
HOXA13	chr7	8.711684179	0.9735
RPS6KB2	chr11	8.708542485	0.9726
BTN2A1	chr6	8.708542485	0.9726
RLN2	chr9	8.708542485	0.9726
PCBD1	chr10	8.702241961	0.9706
NIPAL1	chr4	8.702241961	0.9706
STBD1	chr4	8.702241961	0.9706
CHAC2	chr2	8.699083086	0.9696
TXNDC9	chr2	8.699083086	0.9696
SPATA18	chr4	8.699083086	0.9696
ZWINT	chr10	8.692741024	0.9674
CENPJ	chr13	8.692741024	0.9674
C4orf14	chr4	8.692741024	0.9674
IFI35	chr17	8.689561254	0.9663
TIGD3	chr11	8.686370959	0.9651
RPS6KA5	chr14	8.686370959	0.9651
BEND3	chr6	8.679972643	0.9626
DOCK4	chr7	8.673549357	0.96
EXOC1	chr4	8.670323417	0.9587
GLUL	chr1	8.667093797	0.9573
TMEM99	chr17	8.667093797	0.9573
SHARPIN	chr8	8.667093797	0.9573
TSPAN15	chr10	8.663853373	0.9558
OTX1	chr2	8.663853373	0.9558
SLC35C2	chr20	8.663853373	0.9558
ALG11	chr13	8.66060922	0.9543
PSMC2	chr7	8.66060922	0.9543
FIP1L1	chr4	8.657357755	0.9528
PANK2	chr20	8.654095364	0.9512
HAX1	chr1	8.650829169	0.9496
ARHGAP18	chr6	8.650829169	0.9496
LMOD1	chr1	8.644270905	0.9462
FAM160A2	chr11	8.637682692	0.9427
PNMA1	chr14	8.637682692	0.9427
SS18L1	chr20	8.637682692	0.9427
TMEM14A	chr6	8.637682692	0.9427
GAL3ST4	chr7	8.637682692	0.9427

GANAB	chr11	8.634379084	0.9408
CTSC	chr11	8.634379084	0.9408
CSPP1	chr8	8.634379084	0.9408
POLD3	chr11	8.621081152	0.9328
PACRGL	chr4	8.621081152	0.9328
ZNF772	chr19	8.611028487	0.9263
CCDC66	chr3	8.611028487	0.9263
GPC2	chr7	8.611028487	0.9263
PC	chr11	8.607659507	0.924
CHCHD3	chr7	8.607659507	0.924
RBM25	chr14	8.604286348	0.9216
TMEM223	chr11	8.590706802	0.9116
PIBF1	chr13	8.590706802	0.9116
TMCO1	chr1	8.587293725	0.9089
SLC35C1	chr11	8.587293725	0.9089
LOC221710	chr6	8.587293725	0.9089
CNPY4	chr7	8.587293725	0.9089
ZSCAN23	chr6	8.576998221	0.9005
CCDC75	chr2	8.573552519	0.8976
COPS6	chr7	8.573552519	0.8976
NACAP1	chr8	8.570098568	0.8946
TMEM175	chr4	8.566632523	0.8915
RIBC1	chrx	8.566632523	0.8915
DDX18	chr2	8.563161945	0.8883
LPCAT3	chr12	8.55269215	0.8785
C14orf1	chr14	8.55269215	0.8785
TRIM5	chr11	8.542149689	0.868
STX5	chr11	8.538619639	0.8643
VCPIP1	chr8	8.535077041	0.8606
C13orf37	chr13	8.52796955	0.8529
SLC35B1	chr17	8.52796955	0.8529
THAP1	chr8	8.52796955	0.8529
NRSN2	chr20	8.517244193	0.8409
STK3	chr8	8.513652598	0.8367

Group 2: Intermediate grp genes with NLCS ≥8-8.5; FDR for noise background (BG) and histone mark separation ≤0.05

Gene	Chromome	Cancer_NLCS (Normalized locus-specific chromatin state)	Probability to be signal
ATP5S	chr14	8.510048081	0.825
MCM3	chr6	8.506438504	0.825
MTFR1	chr8	8.502815896	0.825
TUT1	chr11	8.499188155	0.823
LDHB	chr12	8.49555127	0.823
STX3	chr11	8.484577223	0.823
CREB3L2	chr7	8.48090327	0.823

DCAF13	chr8	8.466105019	0.822
TPTE2P1	chr13	8.462383662	0.822
CHMP2B	chr3	8.45864858	0.822
TRIM35	chr8	8.45864858	0.822
TRAPPC2	chrx	8.45864858	0.822
GNA13	chr17	8.454907915	0.822
ATF6B	chr6	8.454907915	0.821
NT5E	chr6	8.451157525	0.821
SIRT3	chr11	8.447393228	0.821
VPS35	chr16	8.447393228	0.821
C11orf31	chr11	8.443623226	0.821
MSL3	chrx	8.432245397	0.821
NCAPG2	chr7	8.428435551	0.821
PPP1R3B	chr8	8.424615617	0.821
MMP20	chr11	8.420781333	0.821
IVNS1ABP	chr1	8.416941051	0.821
MALAT1	chr11	8.416941051	0.821
FAM53A	chr4	8.416941051	0.82
ITFG2	chr12	8.409225441	0.82
POMP	chr13	8.409225441	0.82
ZNF254	chr19	8.409225441	0.82
CUX1	chr7	8.409225441	0.82
DDX11	chr12	8.405349979	0.82
TMEM80	chr11	8.401468345	0.82
RTTN	chr18	8.389756084	0.819
TPI1P2	chr7	8.389756084	0.819
ZNF277	chr7	8.381893308	0.819
C14orf138	chr14	8.37794796	0.819
LOC100133669	chr8	8.37794796	0.819
WFS1	chr4	8.373987445	0.819
PARP11	chr12	8.366038019	0.812
EN1	chr2	8.366038019	0.812
XRCC6	chr22	8.366038019	0.812
SSSCA1	chr11	8.362049012	0.812
DCAF16	chr4	8.362049012	0.812
PCNA	chr20	8.350010963	0.805
PCNAAS	chr20	8.350010963	0.805
CDC42BPG	chr11	8.333799387	0.805
RWDD2B	chr21	8.329715626	0.8
TUBAL3	chr10	8.325624769	0.8
ZNF321	chr19	8.304985535	0.8
CXorf42	chrx	8.296645533	0.79
CLN5	chr13	8.292459683	0.79
C11orf35	chr11	8.288257038	0.79
PPIB	chr15	8.288257038	0.774
RPS19BP1	chr22	8.284046744	0.774
TBC1D17	chr19	8.279819484	0.774

CST6	chr11	8.275584457	0.774
ZNF491	chr19	8.271336962	0.773
ZNF773	chr19	8.271336962	0.8773
NFATC4	chr14	8.267072241	0.773
CYP46A1	chr14	8.258509503	0.772
MOSPD3	chr7	8.258509503	0.772
GINS4	chr8	8.254211362	0.772
PCMT1	chr6	8.249895639	0.758
RBCK1	chr20	8.241234803	0.758
DIS3	chr13	8.236880028	0.758
COX5B	chr2	8.232516864	0.758
WBP2NL	chr22	8.232516864	0.746
ZRANB2	chr1	8.228135653	0.746
DNA2	chr10	8.210491148	0.746
MAN2B2	chr4	8.19262324	0.746
IL15RA	chr10	8.183605609	0.745
AP2A2	chr11	8.183605609	0.732
UBR7	chr14	8.179078057	0.732
COQ2	chr4	8.146965728	0.732
C21orf56	chr21	8.142316424	0.723
NCOA4	chr10	8.137657212	0.723
SLC25A15	chr13	8.137657212	0.723
OPN5	chr6	8.132977764	0.723

Group 3: Intermediate grp genes with NLCS ≥7.5-8.5; FDR for noise (BG) and histone mark separation ≤0.05

Gene	Chromome	Cancer_NLCS (Normalized locus specific chromatin state)	Probability to be signal
TNFAIP1	chr17	8.128288245	0.7317
MRPS18C	chr4	8.128288245	0.7317
FRMD8	chr11	8.114122321	0.7045
RASSF10	chr11	8.099810661	0.6759
POLR2B	chr4	8.099810661	0.6759
TMTC3	chr12	8.095011901	0.666
MCM4	chr8	8.095011901	0.666
C7orf59	chr7	8.085360917	0.646
DDX43	chr6	8.070764981	0.6149
SSBP1	chr7	8.065863079	0.6044
LY6E	chr8	8.060949867	0.5938
YWHAZ	chr8	8.041117013	0.5505
TAF4	chr20	8.036118652	0.5396
LOC388796	chr20	8.026064141	0.5176
PPDPF	chr20	8.010852553	0.4846
USP16	chr21	8.010852553	0.4846
NUDT15	chr13	8.000619775	0.4627
WRAP53	chr17	7.990313898	0.4409

SLC35F5	chr2	7.990313898	0.4409
PRDX5	chr11	7.985136067	0.4301
FER1L4	chr20	7.979933872	0.4194
CYTH1	chr17	7.974718585	0.4088
RFC3	chr13	7.958952845	0.3774
MYO6	chr6	7.958952845	0.3774
LYN	chr8	7.958952845	0.3774
GRHL2	chr8	7.958952845	0.3774
ZNF433	chr19	7.916040665	0.2991
ATP5O	chr21	7.916040665	0.2991
HPS5	chr11	7.899616736	0.2723
PRMT3	chr11	7.883003678	0.2469
MGC87042	chr7	7.883003678	0.2469
PSMA2	chr7	7.854880847	0.2083
CFL1	chr11	7.83197855	0.1806
NAPG	chr18	7.83197855	0.1806
SEC63	chr6	7.83197855	0.1806
CNOT4	chr7	7.83197855	0.1806
FAM160B1	chr10	7.814563241	0.1617
GCC2	chr2	7.796928645	0.1444
PARP14	chr3	7.796928645	0.1444
PHRF1	chr11	7.791006561	0.139
PTPN4	chr2	7.791006561	0.139
EVC	chr4	7.785053526	0.1337
SOCS4	chr14	7.779082394	0.1286
TRPM7	chr15	7.779082394	0.1286
NDUFV1	chr11	7.773086444	0.1237
ZNF415	chr19	7.767058848	0.1189

Group 4: Least confidence ChIP-seq identfied H2.Z regulated genes with NLCS cutoff ≤8; FDR for noise (BG) and histone mark separation ≥0.05

Gene	Chromome	Cancer_NLCS (Normalized locus-specific chromatin state)	Probability to be signal
DAK	chr11	7.761012614	<0.1
KIAA1310	chr2	7.761012614	<0.1
ZNF470	chr19	7.742706902	<0.1
TATDN1	chr8	7.72417275	<0.1
CD83	chr6	7.711680738	<0.1
LDLRAD3	chr11	7.70539739	<0.1
NF1	chr17	7.70539739	<0.1
CABYR	chr18	7.70539739	<0.1
TBX18	chr6	7.70539739	<0.1
CSTF1	chr20	7.692741024	<0.1
PSMA7	chr20	7.692741024	<0.1
CHPF2	chr7	7.692741024	<0.1
CTSL2	chr9	7.692741024	<0.1
DNAJC2	chr7	7.679972643	<0.1

C4orf19	chr4	7.673549357	<0.1
SNHG11	chr20	7.66060922	<0.1
CCDC71	chr3	7.66060922	<0.1
SART1	chr11	7.654098945	<0.1
PFKFB3	chr10	7.647551965	<0.1
PELI3	chr11	7.62774544	<0.1
MFHAS1	chr8	7.62774544	<0.1
C14orf118	chr14	7.621077488	<0.1
RBM5	chr3	7.607663205	<0.1
DLD	chr7	7.607663205	<0.1
MCPH1	chr8	7.607663205	<0.1
CDC42EP2	chr11	7.594115557	<0.1
ZKSCAN1	chr7	7.594115557	<0.1
NUDT18	chr8	7.594115557	<0.1
RRM1	chr11	7.580439482	<0.1
C3orf52	chr3	7.580439482	<0.1
RTN3	chr11	7.573548733	<0.1
AASDH	chr4	7.573548733	<0.1
PRPS2	chrx	7.573548733	<0.1
LHFP	chr13	7.566632523	<0.1
CHMP1B	chr18	7.566632523	<0.1
NRXN2	chr11	7.559682997	<0.1
ACTR3B	chr7	7.55269215	<0.1
MCAT	chr22	7.545674983	<0.1
MYBL2	chr20	7.538615759	<0.1
FAM40B	chr7	7.538615759	<0.1
SYAP1	chrx	7.538615759	<0.1
ZNF549	chr19	7.524408507	<0.1
NDUFAF4	chr6	7.524408507	<0.1
CDC42	chr1	7.517244193	<0.1
C11orf46	chr11	7.517244193	<0.1
LCP1	chr13	7.517244193	<0.1
TMEM184B	chr22	7.510052039	<0.1
FBXL5	chr4	7.510052039	<0.1
KIAA1191	chr5	7.510052039	<0.1
ARPC1A	chr7	7.502815896	<0.1
TMEM168	chr7	7.49555127	<0.1
SAPS3	chr11	7.488241843	<0.1
KBTBD7	chr13	7.473527177	<0.1
CDK5R1	chr17	7.473527177	<0.1
XPA	chr9	7.473527177	<0.1
MTX3	chr5	7.466105019	<0.1
PL-5283	chr7	7.466105019	<0.1
RNF141	chr11	7.458652681	<0.1
PDDC1	chr11	7.451153403	<0.1
ARF6	chr14	7.451153403	<0.1
ZNF33A	chr10	7.443623226	<0.1

METTL12	chr11	7.443623226	<0.1
WDR73	chr15	7.443623226	<0.1
ACAD9	chr3	7.443623226	<0.1
NCRNA00171	chr6	7.443623226	<0.1
PRKDC	chr8	7.443623226	<0.1
MAP2K5	chr15	7.436045209	<0.1
LAMP1	chr13	7.428435551	<0.1
CLN6	chr15	7.428435551	<0.1
DKK3	chr11	7.420785542	<0.1
EXOC5	chr14	7.420785542	<0.1
CLTC	chr17	7.420785542	<0.1
SNRPB	chr20	7.420785542	<0.1
DCAF5	chr14	7.413086288	<0.1
VWA3B	chr2	7.413086288	<0.1
PPP1R3D	chr20	7.413086288	<0.1
L3MBTL3	chr6	7.413086288	<0.1
CTNND1	chr11	7.405354234	<0.1
BRF2	chr8	7.405354234	<0.1
C1orf26	chr1	7.397571962	<0.1
NEK4	chr3	7.397571962	<0.1
SLC35B3	chr6	7.397571962	<0.1
FNBP1L	chr1	7.381888983	<0.1
SLC30A9	chr4	7.381888983	<0.1
PUS7	chr7	7.381888983	<0.1
HDHD1A	chrx	7.381888983	<0.1
TMEM33	chr4	7.373987445	<0.1
MOBKL1A	chr4	7.373987445	<0.1
DDB2	chr11	7.366042392	<0.1
RB1	chr13	7.358044548	<0.1
C22orf28	chr22	7.358044548	<0.1
COMMD8	chr4	7.358044548	<0.1
HBS1L	chr6	7.350010963	<0.1
GGCX	chr2	7.333799387	<0.1
LRRC59	chr17	7.325629266	<0.1
DUSP22	chr6	7.325629266	<0.1
COMMD9	chr11	7.317403569	<0.1
OAS2	chr12	7.317403569	<0.1
C7orf13	chr7	7.317403569	<0.1
SLC25A44	chr1	7.309139799	<0.1
DCUN1D5	chr11	7.300819273	<0.1
C14orf79	chr14	7.300819273	<0.1
CCDC77	chr12	7.292459683	<0.1
GNPDA2	chr4	7.284042115	<0.1
C11orf80	chr11	7.275584457	<0.1
LOC100132707	chr7	7.249900378	<0.1
ZNF398	chr7	7.232516864	<0.1
SLC25A32	chr8	7.232516864	<0.1

ACO1	chr9	7.223741097	<0.1
XPNPEP1	chr10	7.214921332	<0.1
ZNF445	chr3	7.214921332	<0.1
C18orf19	chr18	7.206047317	<0.1
UBE2V1	chr20	7.206047317	<0.1
USP6NL	chr10	7.197108548	<0.1
TP53	chr17	7.197108548	<0.1
OCIAD1	chr4	7.197108548	<0.1
FAM48A	chr13	7.188123944	<0.1
HIST1H1B	chr6	7.188123944	<0.1
PKIB	chr6	7.188123944	<0.1
LYPLA1	chr8	7.17907308	<0.1
SFXN4	chr10	7.169975094	<0.1
IQSEC2	chrx	7.169975094	<0.1
CNIH	chr14	7.160809288	<0.1
GPCPD1	chr20	7.132977764	<0.1
CNOT7	chr8	7.132977764	<0.1
USP5	chr12	7.114117114	<0.1
NRF1	chr7	7.114117114	<0.1
GTF2E2	chr8	7.114117114	<0.1
PEX16	chr11	7.104598754	<0.1
ZNF610	chr19	7.095006625	<0.1
CELF1	chr11	7.085360917	<0.1
WIPF1	chr2	7.085360917	<0.1
GKAP1	chr9	7.065863079	<0.1
KIAA1407	chr3	7.056019866	<0.1
NAA38	chr7	7.056019866	<0.1
GCC1	chr7	7.046098116	<0.1
GCA	chr2	7.036118652	<0.1
FLVCR2	chr14	7.026069676	<0.1
ADAM10	chr15	7.026069676	<0.1
PHOSPHO1	chr17	7.026069676	<0.1
KDM5C	chrx	7.026069676	<0.1
RSPH4A	chr6	7.015939067	<0.1
REEP4	chr8	7.015939067	<0.1
HOXB4	chr17	7.005748043	<0.1
TNPO3	chr7	7.005748043	<0.1
ZFPL1	chr11	6.97471285	<0.1
POLA2	chr11	6.97471285	<0.1
PDS5A	chr4	6.97471285	<0.1
RPL30	chr8	6.97471285	<0.1
C1orf203	chr1	6.964225332	<0.1
CAT	chr11	6.964225332	<0.1
RNF19A	chr8	6.964225332	<0.1
GAK	chr4	6.953661018	<0.1
SPINK2	chr4	6.953661018	<0.1
C4orf52	chr4	6.943007049	<0.1

GOLT1B	chr12	6.93228563	<0.1
USPL1	chr13	6.93228563	<0.1
ZNF709	chr19	6.93228563	<0.1
C7orf43	chr7	6.921472036	<0.1
AZIN1	chr8	6.921472036	<0.1
PPAPDC2	chr9	6.921472036	<0.1
CR1L	chr1	6.910588769	<0.1
BAD	chr11	6.899610694	<0.1
TEP1	chr14	6.899610694	<0.1
EHMT2	chr6	6.899610694	<0.1
SNX1	chr15	6.888560618	<0.1
EIF4EBP2	chr10	6.877425251	<0.1
MAF1	chr8	6.877425251	<0.1
FAU	chr11	6.866190902	<0.1
RPL23AP53	chr8	6.866190902	<0.1
PPP1CA	chr11	6.854880847	<0.1
MCM8	chr20	6.854880847	<0.1
INTS5	chr11	6.843468863	<0.1
TAB1	chr22	6.843468863	<0.1
CORO2A	chr9	6.83197855	<0.1
OFD1	chrx	6.820383223	<0.1
CPSF7	chr11	6.808706815	<0.1
TMEM65	chr8	6.808706815	<0.1
NUP98	chr11	6.796935132	<0.1
C14orf45	chr14	6.796935132	<0.1
MANEA	chr6	6.796935132	<0.1
DSE	chr6	6.796935132	<0.1
SLC25A29	chr14	6.785053526	<0.1
CDK2	chr12	6.761005964	<0.1
TMEM18	chr2	6.761005964	<0.1
ZNF525	chr19	6.748836886	<0.1
NRBF2	chr10	6.736564289	<0.1
CSK	chr15	6.72417275	<0.1
FUT10	chr8	6.72417275	<0.1
SLC11A2	chr12	6.71168762	<0.1
SEC61A2	chr10	6.699079615	<0.1
TMEM134	chr11	6.699079615	<0.1
ZNF625	chr19	6.699079615	<0.1
SH3BGR	chr21	6.699079615	<0.1
RBM4B	chr11	6.673542291	<0.1
NCOA3	chr20	6.66060922	<0.1
C6orf167	chr6	6.64755916	<0.1
ZNF563	chr19	6.634375454	<0.1
SLC16A10	chr6	6.607655808	<0.1
C13orf1	chr13	6.594115557	<0.1
POGK	chr1	6.566632523	<0.1
YTHDF1	chr20	6.566632523	<0.1

MRFAP1L1	chr4	6.566632523	<0.1
CAPZA2	chr7	6.552699834	<0.1
CYP2R1	chr11	6.510044124	<0.1
DHX35	chr20	6.49555127	<0.1
BOD1L	chr4	6.49555127	<0.1
METTL3	chr14	6.480895194	<0.1
INCENP	chr11	6.466105019	<0.1
FLJ40852	chr7	6.451161647	<0.1
EIF3E	chr8	6.451161647	<0.1
ZFP91	chr11	6.420785542	<0.1
ZFP91-CNTF	chr11	6.420785542	<0.1
C20orf11	chr20	6.420785542	<0.1
PRSS35	chr6	6.420785542	<0.1
EBAG9	chr8	6.420785542	<0.1
TMEM179B	chr11	6.405345724	<0.1
PPP1R13B	chr14	6.373996142	<0.1
ZFYVE26	chr14	6.325620272	<0.1
FBXL4	chr6	6.325620272	<0.1
CDCA3	chr12	6.29245048	<0.1
DLEU2	chr13	6.29245048	<0.1
ZCCHC4	chr4	6.29245048	<0.1
LMAN2L	chr2	6.275584457	<0.1
CDC37L1	chr9	6.275584457	<0.1
CEP290	chr12	6.258518925	<0.1
ZNF582	chr19	6.258518925	<0.1
OVOL2	chr20	6.258518925	<0.1
C10orf119	chr10	6.241230036	<0.1
EFCAB6	chr22	6.223750749	<0.1
ADRM1	chr20	6.206037546	<0.1
TP53INP2	chr20	6.188123944	<0.1
SNHG1	chr11	6.151595024	<0.1
PYGO1	chr15	6.151595024	<0.1
SNORA57	chr11	6.114117114	<0.1
RNF219	chr13	6.114117114	<0.1
FBXO30	chr6	6.114117114	<0.1
AKAP11	chr13	6.095017177	<0.1
TRMT112	chr11	6.036107659	<0.1
C15orf44	chr15	5.995484519	<0.1
FYN	chr6	5.995484519	<0.1
HELQ	chr4	5.97471285	<0.1
ING3	chr7	5.953661018	<0.1
WDR67	chr8	5.888572794	<0.1
C5orf43	chr5	5.866190902	<0.1
ZNF248	chr10	5.843481425	<0.1
TMEM177	chr2	5.820383223	<0.1
E2F1	chr20	5.820383223	<0.1
HEATR5B	chr2	5.796935132	<0.1

PPM1A	chr14	5.773073253	<0.1
KIAA0247	chr14	5.773073253	<0.1
RRP8	chr11	5.724186396	<0.1
TMEM66	chr8	5.699079615	<0.1
CTTN	chr11	5.673556424	<0.1
TSHR	chr14	5.673556424	<0.1
NCAPG	chr4	5.621084817	<0.1
NUDT2	chr9	5.621084817	<0.1
TCEANC	chrx	5.566632523	<0.1
MYLK	chr3	5.538631278	<0.1
NFXL1	chr4	5.510044124	<0.1
FAM60A	chr12	5.451145159	<0.1
YWHAB	chr20	5.420785542	<0.1
PRKAR2B	chr7	5.389738879	<0.1
NUP205	chr7	5.389738879	<0.1
POM121L2	chr6	5.358044548	<0.1
TM9SF2	chr13	5.32563826	<0.1
CHD7	chr8	5.258518925	<0.1
ING4	chr12	5.151615317	<0.1
MRFAP1	chr4	5.114117114	<0.1
COX18	chr4	5.075660979	<0.1
