# Supplementary material for: Gene dysregulation by histone variant H2A.Z in bladder cancer
Source: Epigenetics Chromatin. 2013 Oct 16;6:34. doi: 10.1186/1756-8935-6-34 (PMC3853418; doi:10.1186/1756-8935-6-34)
Supplement: Additional file 3: Table S2 — Differential gene expression in cancer vs. normal cells. [file 1756-8935-6-34-S3.pdf]

Supplementary Table S2. Differential gene expression in cancer vs. normal cells.

SYMBOL	Flod Change (LD611/UROtsa)	up/down
LCP1	42.36870184	UP
AXL	40.33120205	UP
FSTL1	40.10045317	UP
CYGB	37.50927835	UP
FLNC	24.77902885	UP
GTSF1	21.49654219	UP
SYTL3	19.76977951	UP
TMEM98	19.06712963	UP
UCA1	18.98542601	UP
KRT6C	17.13527851	UP
OLR1	17.12164675	UP
IL13RA2	16.25236364	UP
FBN2	15.81003584	UP
MFAP5	15.15339039	UP
FLJ22662	14.89548577	UP
CPVL	14.3004386	UP
ERP27	14.24414977	UP
BIRC3	13.93728981	UP
SUNC1	13.20614035	UP
MAGEA10	13.17290837	UP
BIRC2	12.49544304	UP
C20orf100	12.47708071	UP
SFTA1P	12.42626368	UP
CXCL1	12.23502994	UP
SLC15A3	11.98822574	UP
MAGEA6	11.86137281	UP
ACTA2	11.77139762	UP
SERPINE1	11.65530643	UP
LY96	11.4702381	UP
FLJ35282	11.02453988	UP
TIMM10	10.93895349	UP
FGD3	10.78041543	UP
CDA	10.60152284	UP
LOC651397	10.55803305	UP
EGFLAM	10.52043177	UP
FHL1	10.45179234	UP
CST6	10.35436537	UP
LOC644936	10.26868077	UP
ORAOV1	10.10729927	UP
TSPAN4	9.95797227	UP
YAP1	9.693527756	UP
STMN3	9.574749758	UP
CSAG1	9.423974763	UP
RNASE7	9.322473404	UP
VGLL1	9.260869565	UP
KRT7	9.047090399	UP
SH3KBP1	8.896718666	UP
IGFBP7	8.81701991	UP

SYMBOL	Flod Change (LD611/UROtsa)	up/down
MMP12	-90.82406015	Down
LGALS7B	-73.76834532	Down
ALDH3A1	-59.88194921	Down
MMP10	-43.91329114	Down
BST2	-41.34776903	Down
LGALS7	-39.69342561	Down
LOC100129681	-39.235	Down
LOC653499	-32.53761062	Down
CYBA	-32.25660122	Down
LOC728910	-32.15357887	Down
IFI44L	-30.34800621	Down
HSPBL2	-28.7303417	Down
UCHL1	-28.54587766	Down
GPX2	-28.31321928	Down
VIM	-25.27762557	Down
CKB	-22.81101512	Down
CKMT1A	-22.2328976	Down
FABP4	-20.7992126	Down
KRT13	-18.76683556	Down
SPRR3	-18.75474563	Down
ECGF1	-18.53341218	Down
LOXL4	-18.24902793	Down
CNTNAP2	-17.93122102	Down
REC8	-17.74788851	Down
SPRR1B	-16.46928571	Down
UGT1A6	-16.38572409	Down
CKMT1B	-16.20332654	Down
LOC649970	-16.1845423	Down
SPRR1A	-15.87526427	Down
THBS2	-15.46797753	Down
SOX2	-14.98017621	Down
GJB6	-14.92641261	Down
S100A8	-14.82703297	Down
MMP1	-13.63163539	Down
PTGS2	-13.53127753	Down
IRX4	-13.02896341	Down
MTAP	-12.00880411	Down
RGMA	-11.93868195	Down
CDKN2A	-11.71066368	Down
MMP13	-11.18786982	Down
TNFSF10	-11.11222249	Down
DSG3	-11.09147965	Down
TNC	-11.08681065	Down
NLRP2	-11.03016241	Down
AKR1C4	-10.60567319	Down
AKR1C2	-10.06441012	Down
C13orf15	-9.819799778	Down
RYR1	-9.618023256	Down

C4BPB	8.439198856	UP	IRX2	-9.413468013	Down
KRT14	8.169166264	UP	BMP7	-9.3017177	Down
LCN2	7.91575391	UP	LY6D	-9.281751825	Down
MAMDC2	7.801349325	UP	SERPINB13	-9.250531915	Down
MAGEA9B	7.799859551	UP	GJB2	-9.092222987	Down
HCLS1	7.72154617	UP	TPM2	-9.032381866	Down
ANKRD1	7.711756374	UP	KLHL35	-8.956830278	Down
G0S2	7.66082691	UP	HIST1H1C	-8.932292612	Down
TPCN2	7.483006912	UP	CALML3	-8.780947529	Down
NPPB	7.469503546	UP	IRF7	-8.475	Down
BEXL1	7.405585914	UP	ISG15	-8.297067737	Down
MAGEA12	7.31969697	UP	DPYSL3	-8.238992042	Down
GALNT12	7.216666667	UP	CRABP2	-8.154262224	Down
STARD10	7.147460595	UP	VSNL1	-8.089455488	Down
ID3	7.117591722	UP	PROS1	-8.023648649	Down
IGFL1	7.110386352	UP	SPOCK1	-8.020692568	Down
GNG11	6.977121771	UP	MGMT	-7.92296405	Down
TIMP2	6.95017236	UP	HIST2H2AA4	-7.849517031	Down
SLC44A3	6.924373202	UP	LYPD3	-7.797326203	Down
LTB	6.76194332	UP	ISG20	-7.667411712	Down
LOC100132535	6.707552223	UP	LAMC3	-7.63671275	Down
WDR66	6.652861662	UP	HSPB1	-7.606884481	Down
LOC100129602	6.621790423	UP	NOTCH3	-7.590332805	Down
41337	6.612021858	UP	AUTS2	-7.573219884	Down
BNIP3	6.452890699	UP	CRIP2	-7.535198279	Down
C12orf59	6.443442054	UP	EGR1	-7.52321297	Down
EPDR1	6.433622415	UP	ERAP2	-7.501397624	Down
PTTG1IP	6.395030148	UP	EMP3	-7.44255029	Down
C15orf52	6.383127476	UP	PTHLH	-7.414020059	Down
BEX4	6.362175525	UP	HCP5	-7.391121192	Down
GLIPR2	6.304225352	UP	CYP26B1	-7.382585752	Down
DPYSL2	6.301084011	UP	KIAA1199	-7.371302073	Down
SELM	6.295409182	UP	NKD2	-7.2	Down
SRPX	6.25485189	UP	LOC650517	-7.175386883	Down
MXRA5	6.09971366	UP	CXCL10	-7.153810409	Down
RAB3IL1	6.069954128	UP	IFI44	-7.109203576	Down
PTPN20A	5.985283297	UP	LOC100134134	-7.082132565	Down
C9orf169	5.961356179	UP	ODC1	-7.051240561	Down
RNY4	5.960946344	UP	RPP25	-7.022211939	Down
CSAG3B	5.949494949	UP	MLLT11	-6.836419056	Down
TIMP3	5.927272727	UP	HLA-A29.1	-6.783653201	Down
SPARC	5.853125	UP	KRTCAP3	-6.721123165	Down
C11orf70	5.749676943	UP	SLC4A11	-6.686983471	Down
TMEM123	5.731285704	UP	PTPRZ1	-6.656080558	Down
FCGRT	5.719632305	UP	HIST2H2AA3	-6.50223992	Down
HDHD1A	5.700576923	UP	RAB7B	-6.483870968	Down
CD99	5.665512324	UP	HIST1H2AC	-6.414618778	Down
MAGEA4	5.618685121	UP	NAB1	-6.396742671	Down
PTGES	5.592217135	UP	PLEKHG4	-6.371556217	Down
MCOLN2	5.529520295	UP	CX3CL1	-6.319444444	Down
IL7R	5.510262989	UP	CCNA1	-6.149654643	Down

ZNF256	5.491764706	UP	NPW	-6.086956522	Down
TGM2	5.458096591	UP	FZD1	-6.066666667	Down
APLN	5.45029554	UP	LOC284023	-5.971179039	Down
SRPX2	5.400993612	UP	IL20RB	-5.938413509	Down
ELOVL4	5.378778135	UP	CSPG4	-5.893666927	Down
NT5E	5.361380211	UP	OAS2	-5.840365828	Down
KLK5	5.356915233	UP	CYP1B1	-5.799001248	Down
STC1	5.340145985	UP	FBLN2	-5.691582003	Down
IL32	5.338992974	UP	MX1	-5.669712878	Down
NNMT	5.321966693	UP	IFIT1	-5.65681631	Down
KRT80	5.319012179	UP	OASL	-5.641247834	Down
ZNF22	5.254996157	UP	IL1F9	-5.574825986	Down
SLCO2A1	5.25498008	UP	GAPDHL6	-5.56010568	Down
SAA1	5.248837209	UP	ADAM23	-5.520467836	Down
COMMD7	5.214929727	UP	IL1RN	-5.517313746	Down
LIN28B	5.189542484	UP	SEMA3A	-5.48652064	Down
STS-1	5.187788018	UP	RTP4	-5.480102696	Down
PDGFRL	5.14974802	UP	MARCKSL1	-5.414915724	Down
LOC440030	5.131465517	UP	FIBCD1	-5.397952655	Down
SERPING1	5.110152621	UP	CLDN7	-5.375845547	Down
SH3GL2	5.105643994	UP	MFGE8	-5.375016611	Down
ITGB2	5.044694909	UP	GAL	-5.363028953	Down
FOSL1	5.044535073	UP	HLA-B	-5.361969112	Down
GLIPR1	5.041666667	UP	CCL5	-5.33534223	Down
GLDC	5.026809651	UP	PRR16	-5.327493261	Down
LIMCH1	5	UP	LOC100128288	-5.274177856	Down
SDPR	4.998588567	UP	ECM1	-5.23683119	Down
CSF2	4.979770735	UP	HSD17B1	-5.231354642	Down
MAGEA3	4.948153409	UP	LOC388494	-5.193083573	Down
IL1A	4.946689113	UP	PLCH2	-5.176889376	Down
FLJ10986	4.935860058	UP	DSC3	-5.150338103	Down
CMBL	4.874858597	UP	SLC9A3R1	-5.126210059	Down
PRSS8	4.859508547	UP	GNG4	-5.119949495	Down
PLIN2	4.833864796	UP	HLA-F	-5.048695488	Down
IMMP2L	4.819267823	UP	STAG3L3	-5.033996683	Down
LOC341315	4.81553719	UP	GBP6	-5.015418502	Down
FZD2	4.749109052	UP	KRT16	-4.976564733	Down
HOXD10	4.735189151	UP	TYMP	-4.966081163	Down
DSCR6	4.724613687	UP	IRS1	-4.962354826	Down
DNAJC15	4.724319579	UP	NINJ2	-4.955289673	Down
OGFRL1	4.721813251	UP	LOC100132585	-4.9375	Down
MSX1	4.712183637	UP	LHX6	-4.927665921	Down
FUCA1	4.709588093	UP	GPNMB	-4.923605634	Down
LHX1	4.705636743	UP	RBBP8	-4.910037417	Down
TP53I13	4.702187667	UP	CXCR7	-4.87469352	Down
RTTN	4.682654112	UP	HIST1H2BD	-4.836938436	Down
LOC644695	4.658798283	UP	CLDN11	-4.815209808	Down
RHOBTB3	4.647623862	UP	NUDT7	-4.794749403	Down
C11orf80	4.643037064	UP	LMOD3	-4.770230933	Down
CTTN	4.640232108	UP	LOC100129211	-4.763937282	Down
IL1B	4.625249917	UP	TNFSF9	-4.738553417	Down

ANTXR2	4.615611193	UP	CYP2S1	-4.733353329	Down
RRAS2	4.604786546	UP	CATSPER2	-4.723910483	Down
DRAM1	4.596741344	UP	CIDEB	-4.720128308	Down
DUSP5	4.590569932	UP	TUSC1	-4.717299578	Down
MMD	4.55017428	UP	OAS1	-4.697163769	Down
NFE2L3	4.545681063	UP	STAG3L2	-4.696341683	Down
TOX2	4.53256705	UP	ETFB	-4.691662297	Down
PPFIA1	4.528189911	UP	C5	-4.685728493	Down
RAB34	4.47632762	UP	LOC100132391	-4.677325581	Down
SFRP1	4.473214286	UP	IRX3	-4.676841796	Down
LPAR1	4.465763195	UP	KLF13	-4.671901204	Down
IGFBP4	4.462324877	UP	KRT15	-4.647368421	Down
FLJ40504	4.445570353	UP	PLEKHA4	-4.62260011	Down
LOC100128975	4.440965427	UP	PALMD	-4.604803493	Down
ZNF702P	4.41969697	UP	FLJ36131	-4.563879103	Down
MAP4K2	4.411340753	UP	TLE4	-4.559673832	Down
ID1	4.411179335	UP	HLA-H	-4.559233364	Down
FNBP1	4.3968509	UP	TMEM79	-4.552298608	Down
WWC1	4.392466765	UP	GPC1	-4.551683502	Down
FAM92A1	4.357714896	UP	ODZ3	-4.542731921	Down
ALG1L	4.351601423	UP	HIST2H2BE	-4.523364486	Down
MGC39900	4.319327731	UP	STOM	-4.519922879	Down
HOXD11	4.311613242	UP	LOC728903	-4.507655293	Down
CLDN23	4.308705613	UP	HIST2H2AC	-4.506287844	Down
HOXB7	4.29468599	UP	FST	-4.503097708	Down
SLC2A10	4.252427184	UP	GSTT1	-4.492733564	Down
ZIK1	4.248985115	UP	NRG4	-4.457304527	Down
MERTK	4.248056995	UP	S100A14	-4.447157083	Down
SEMA4D	4.206144129	UP	AHNAK2	-4.437673515	Down
KRT18P13	4.187410587	UP	APRT	-4.421072945	Down
KHDC1L	4.183582917	UP	ZDHHC11	-4.417537747	Down
EPN3	4.156639566	UP	SNCA	-4.413021967	Down
STX3	4.138461538	UP	MGST1	-4.409045226	Down
TMEM133	4.136297376	UP	HIST1H2BK	-4.405210933	Down
SLC16A2	4.135319454	UP	PTK6	-4.404494382	Down
CHPT1	4.112863071	UP	MN1	-4.394970986	Down
NMT2	4.107271997	UP	ANKRD29	-4.383316274	Down
DDAH1	4.101542112	UP	SOX21	-4.381054131	Down
ARHGDIB	4.089737311	UP	TSPAN7	-4.370643432	Down
MGC87042	4.085068935	UP	KRT17P3	-4.364854272	Down
TMED5	4.068857129	UP	RASIP1	-4.359447664	Down
MPP1	4.063953488	UP	SERPINI1	-4.324400564	Down
RIPK2	4.06213078	UP	TRIM4	-4.309056604	Down
LOC100129673	4.058455115	UP	DDX60	-4.298709281	Down
AZIN1	4.030336581	UP	IFIT3	-4.295454545	Down
DLEU2	4.02042332	UP	GPR68	-4.293413174	Down
NAMPT	3.986061993	UP	LOC344887	-4.267451299	Down
BNIP3L	3.978344465	UP	SLC16A9	-4.236658933	Down
CERCAM	3.975911458	UP	LOC728809	-4.227691366	Down
LOC254398	3.975778547	UP	LOC730313	-4.195321446	Down
RNY1	3.972794823	UP	RSAD2	-4.170168067	Down

NRG1	3.970352802	UP	KLK11	-4.152921674	Down
CAP2	3.961355286	UP	LOC648852	-4.13400335	Down
EHD1	3.908209751	UP	USP18	-4.129445235	Down
LOC646723	3.904495066	UP	C14orf85	-4.122843018	Down
CYBRD1	3.904072044	UP	LOC100129362	-4.111809951	Down
MSRB2	3.901337022	UP	MRPS11	-4.086807321	Down
IL8	3.897544578	UP	FAM175A	-4.055727235	Down
TNFAIP2	3.895896914	UP	GRIPAP1	-4.052352049	Down
EPHA1	3.890722291	UP	COL7A1	-4.051822789	Down
LOC647307	3.878733572	UP	PBX3	-4.048564593	Down
POLM	3.87254902	UP	IFI6	-4.03229381	Down
AMD1	3.871304259	UP	PKP1	-4.032085561	Down
LOC100132240	3.851194539	UP	LOC389765	-4.004570123	Down
ABHD8	3.849675325	UP	KIAA1751	-3.979142857	Down
LOC642477	3.818510484	UP	ISL1	-3.965339233	Down
KCNN4	3.816091954	UP	CHURC1	-3.952495262	Down
PCGF2	3.814637483	UP	IGFBP2	-3.942307692	Down
STK3	3.795478036	UP	PTMS	-3.930798219	Down
FADD	3.792809177	UP	CDKN2AIPNL	-3.929579089	Down
SMO	3.777777778	UP	PLA2G4A	-3.917560322	Down
UGCG	3.768044237	UP	TCTEX1D2	-3.91210465	Down
C10orf47	3.767154846	UP	DBC1	-3.906902087	Down
EMP1	3.758797282	UP	QRFPR	-3.889220768	Down
MTF2	3.746380925	UP	LCMT2	-3.883734416	Down
QPCT	3.729807005	UP	SLC2A5	-3.880424746	Down
DENND2A	3.727881041	UP	AQP3	-3.878141299	Down
ESM1	3.724047306	UP	TCAM1	-3.877495953	Down
LOC399965	3.703207471	UP	GTF2IP1	-3.872823618	Down
ENG	3.699273337	UP	EID2B	-3.858468012	Down
KRT8	3.692824052	UP	CBS	-3.854251012	Down
ADCK2	3.687799272	UP	CCBE1	-3.828716969	Down
PSG4	3.686850153	UP	TMEM17	-3.801854975	Down
SCNN1A	3.685468451	UP	TRIM22	-3.7879046	Down
CPNE1	3.676093254	UP	KRT6B	-3.772259942	Down
HRASLS3	3.663589152	UP	CD58	-3.769331586	Down
DCBLD2	3.663567771	UP	PIK3IP1	-3.758775205	Down
CPA4	3.659188956	UP	DYNC2H1	-3.746847415	Down
WDR72	3.653574234	UP	LOC100128098	-3.736901536	Down
WFDC2	3.650167224	UP	LOC90586	-3.730164082	Down
SERPINB2	3.63717961	UP	BMS1P5	-3.71918581	Down
LPXN	3.606338616	UP	ASCL2	-3.706792777	Down
PLAT	3.601814516	UP	COL4A5	-3.699459459	Down
C4orf49	3.594013814	UP	LTB4R	-3.698529412	Down
HNRPUL2	3.588235294	UP	STAT1	-3.697059157	Down
WISP3	3.567208272	UP	FLRT2	-3.696245734	Down
RHOT1	3.561294212	UP	LOC255167	-3.679453768	Down
ALDOC	3.554973822	UP	ARMCX2	-3.677133106	Down
MME	3.55	UP	CTXN1	-3.676568758	Down
TXNIP	3.539651294	UP	SHROOM4	-3.646061975	Down
ECHDC3	3.532770928	UP	HSPB8	-3.641287284	Down
MSRA	3.48664605	UP	DUSP19	-3.637171116	Down

DDX43	3.466197183	UP	FAM73A	-3.630300958	Down
B3GNT1	3.459118103	UP	EMILIN2	-3.621621622	Down
KIAA0100	3.447619048	UP	GLB1L2	-3.617244158	Down
MRPL21	3.434929331	UP	ZNF394	-3.599850331	Down
FAM195B	3.43112159	UP	PLSCR1	-3.598370429	Down
FAM65A	3.425582561	UP	EFEMP1	-3.598175118	Down
SOX9	3.422604423	UP	TCEA3	-3.596822244	Down
TC2N	3.415108452	UP	C8orf45	-3.588364301	Down
LYRM1	3.405152562	UP	FKBP14	-3.585479597	Down
ACO1	3.403944672	UP	SYK	-3.585325444	Down
OSAP	3.395381386	UP	IFI35	-3.580645161	Down
C17orf79	3.366404098	UP	KCNK6	-3.577743902	Down
INSIG2	3.358852662	UP	UCP2	-3.566534914	Down
FOXO1	3.324136479	UP	CHD9	-3.561884879	Down
PDZK1IP1	3.313620072	UP	NTRK2	-3.553505535	Down
EPB41L2	3.306240929	UP	C3orf34	-3.552013565	Down
FZD3	3.283692308	UP	IQGAP2	-3.547814208	Down
TMEM14B	3.26713917	UP	MT1E	-3.545698573	Down
AGAP3	3.264683153	UP	HES4	-3.542213884	Down
TSPAN33	3.26067559	UP	MAGED1	-3.537771346	Down
ERI1	3.25972036	UP	HSPB3	-3.52688172	Down
IL1RAPL1	3.258988422	UP	HSPC268	-3.52503872	Down
ALDH7A1	3.249727804	UP	RUNX3	-3.522008679	Down
GOLT1A	3.224896836	UP	JUP	-3.518704729	Down
GALM	3.222847949	UP	LOC439949	-3.517857143	Down
CCL20	3.220544554	UP	SERPINB4	-3.50974359	Down
KIAA1147	3.216972878	UP	C14orf78	-3.509155967	Down
DIRC2	3.209473889	UP	FAM119A	-3.504479456	Down
ANKRD46	3.208788282	UP	PODXL2	-3.482032854	Down
TRNP1	3.208390458	UP	HSPA2	-3.480877104	Down
CTSB	3.202242651	UP	GPX3	-3.479135618	Down
IGHMBP2	3.201254276	UP	ODZ4	-3.476020043	Down
LOC399959	3.19198895	UP	OAS3	-3.472853384	Down
ASPH	3.190230155	UP	ZNF549	-3.46952381	Down
PPP4R4	3.187564767	UP	SEMA3E	-3.46843464	Down
RBM17	3.181453921	UP	LXN	-3.46507177	Down
MET	3.178983834	UP	ZNF14	-3.458756795	Down
C13orf18	3.166146646	UP	INO80C	-3.456013746	Down
SH3GL3	3.165578635	UP	SYCP2	-3.449811321	Down
HOXB5	3.164782867	UP	KRT17	-3.448647731	Down
RRAGD	3.161558442	UP	C20orf127	-3.430504587	Down
LOC647954	3.156207978	UP	GAS8	-3.425531915	Down
ZNF812	3.149709302	UP	LOC100134648	-3.42527699	Down
DAG1	3.149021759	UP	TP53	-3.418642241	Down
HS3ST1	3.138648649	UP	XRCC2	-3.415895711	Down
B3GNT6	3.134525447	UP	NT5C3	-3.412056316	Down
TRAPPC6A	3.118849041	UP	DMRT2	-3.410367171	Down
C11orf41	3.101938263	UP	ECH1	-3.408935527	Down
GPRC5A	3.099760336	UP	HLA-G	-3.408170777	Down
GSDMD	3.095692578	UP	MIR205	-3.401494768	Down
PEA15	3.092297288	UP	NOTCH1	-3.398146432	Down

ZNF415	3.091636364	UP	LOC100128309	-3.388996764	Down
PHCA	3.084803558	UP	FOXQ1	-3.383217666	Down
TMEM118	3.0759832	UP	MAP2	-3.366644159	Down
LOC643790	3.069011281	UP	PRKCQ	-3.364019677	Down
CSRP2	3.067001435	UP	MXD1	-3.359137056	Down
LOC644743	3.054315137	UP	NCRNA00219	-3.358108108	Down
TMEM14D	3.052566824	UP	RASSF9	-3.355820106	Down
SLC45A3	3.047455969	UP	TIGA1	-3.342835895	Down
ST13	3.044405246	UP	ZNF280C	-3.342067989	Down
PBK	3.034491115	UP	CCL2	-3.341990035	Down
USMG5	3.034141514	UP	C15orf63	-3.335216756	Down
PGM1	3.031710984	UP	PLXDC2	-3.333333333	Down
FAM20C	3.028882378	UP	RNF212	-3.327523602	Down
RPS6KA4	3.028751788	UP	OSBP2	-3.326656394	Down
ACSF2	3.024625268	UP	SHC1	-3.321532392	Down
SLCO1B3	3.017062767	UP	CHCHD10	-3.298825962	Down
KCTD12	3.016736402	UP	KLF9	-3.283169245	Down
MUC20	3.009350935	UP	GOLGB1	-3.274719801	Down
MED19	3.006079027	UP	INA	-3.271446863	Down
HSPA1B	3.00451304	UP	C14orf153	-3.265489957	Down
AFAP1	3.002477701	UP	SDHAP2	-3.26527025	Down
CLIC3	2.994093325	UP	LOC100131718	-3.262994087	Down
NEDD4L	2.992878561	UP	LRRFIP1	-3.25729585	Down
OPTN	2.987655942	UP	DDX51	-3.255820557	Down
HSF2BP	2.987334802	UP	LOC388564	-3.250875964	Down
CTDSPL	2.969019483	UP	SERPINH1	-3.245152871	Down
PLEKHF1	2.959818902	UP	DUS3L	-3.244382993	Down
IKBKE	2.956379498	UP	WNT4	-3.239892183	Down
SUGT1	2.954795597	UP	ZNF69	-3.233753637	Down
SIPA1	2.954738878	UP	RHBDD2	-3.220083888	Down
THBD	2.95417124	UP	ARTN	-3.206399279	Down
ATP6V0E2	2.950098232	UP	CBLN2	-3.200601052	Down
RGS10	2.941780359	UP	CYB5D1	-3.19705181	Down
PARVA	2.940304738	UP	ABCC5	-3.195707071	Down
TM7SF2	2.932357395	UP	KRT5	-3.179016089	Down
ETV4	2.932035398	UP	MGC102966	-3.151605232	Down
C11orf83	2.931566588	UP	SH3BGRL	-3.150757077	Down
LARP6	2.929692215	UP	CFH	-3.148257531	Down
EXT1	2.926772516	UP	LOC100129269	-3.138832315	Down
DAB2	2.922857143	UP	HCG2P7	-3.13024682	Down
LOC100130009	2.911777488	UP	MAGT1	-3.12481244	Down
ADA	2.908515418	UP	CCDC125	-3.121227115	Down
HOPX	2.908487085	UP	SAMD9	-3.10715638	Down
NGF	2.903699354	UP	VTCN1	-3.101952278	Down
FGGY	2.89859665	UP	EAF2	-3.098254364	Down
SERPINB7	2.894374508	UP	HIST1H2BJ	-3.09695463	Down
CMTM8	2.893340552	UP	ALKBH2	-3.091354568	Down
PINK1	2.890129409	UP	SERPINB3	-3.058561179	Down
GPR137	2.88683183	UP	CDK6	-3.057168116	Down
STRADB	2.878011118	UP	CLDN5	-3.056827151	Down
CNTN5	2.868864469	UP	ABLIM1	-3.056495319	Down

TRIM17	2.864583333	UP	HSD17B4	-3.054518543	Down
C1S	2.860180412	UP	TSPAN10	-3.053613054	Down
PTPRM	2.857703081	UP	SNURF	-3.046223958	Down
APCDD1L	2.856664638	UP	ARL16	-3.0444933	Down
LOC100129759	2.849514563	UP	LRRC8A	-3.041773596	Down
ZNF185	2.839127686	UP	FYB	-3.038351693	Down
SNTA1	2.837939698	UP	NCOA7	-3.03537684	Down
RB1CC1	2.837321109	UP	TPST1	-3.034116445	Down
LOC149501	2.833883388	UP	LOC100132740	-3.030835425	Down
LOC100129550	2.831743869	UP	HSD17B7	-3.028954273	Down
RNASET2	2.831731552	UP	FLJ46309	-3.019904574	Down
MYO1D	2.828911432	UP	DNAJC12	-3.019534185	Down
RNY5	2.827985075	UP	C9orf167	-3.019019442	Down
LIPC	2.824354244	UP	GCLC	-3.010262989	Down
RN5S9	2.819327108	UP	MZF1	-3.006358627	Down
OTUD6B	2.819290068	UP	ALS2	-2.998919211	Down
SLIT2	2.818853974	UP	NLRP8	-2.989593417	Down
TMEM179B	2.817030706	UP	HIATL2	-2.987977337	Down
FADS3	2.81181486	UP	LRAP	-2.987827183	Down
GIT1	2.810857143	UP	PANX2	-2.985652063	Down
ZSCAN18	2.809187279	UP	PRIC285	-2.984634515	Down
MT1F	2.808265802	UP	USP49	-2.976870314	Down
EPCAM	2.806577042	UP	MMP9	-2.973965287	Down
TMUB1	2.806074326	UP	LOC100129502	-2.967463142	Down
PABPC1	2.796319121	UP	FGFBP1	-2.966322023	Down
FAP	2.795100223	UP	SDR42E1	-2.951363301	Down
TMEM56	2.794232268	UP	LOC100133516	-2.945861405	Down
CD47	2.792751027	UP	ELAVL2	-2.926291793	Down
ROR2	2.787685775	UP	FOSB	-2.926122931	Down
FLJ20920	2.78089172	UP	PI3	-2.920694953	Down
DHCR7	2.777877095	UP	BLZF1	-2.917829806	Down
LOC100131139	2.777700348	UP	LOC100128084	-2.917805864	Down
ZNF114	2.777310924	UP	ARL9	-2.915480018	Down
ADRB2	2.777093175	UP	SERPINB1	-2.88855658	Down
FIS1	2.776842612	UP	NUDT11	-2.888517745	Down
LOC100129323	2.773175542	UP	NSBP1	-2.880970432	Down
SLC25A37	2.772959184	UP	RHOU	-2.877423823	Down
BSCL2	2.770724992	UP	FUT4	-2.872457394	Down
LOC643431	2.765034965	UP	PCDH19	-2.872455902	Down
EFHD2	2.760520693	UP	PAPLN	-2.868935098	Down
POLR2J	2.759827214	UP	SNRPN	-2.862323294	Down
ITGB5	2.759591451	UP	ZNF682	-2.859976356	Down
DNAJB6	2.759317267	UP	KBTBD11	-2.856704981	Down
PTDSS2	2.757688492	UP	SPRR2D	-2.856540084	Down
TMEM45A	2.75647876	UP	H2AFJ	-2.854103343	Down
STIP1	2.753868781	UP	C17orf91	-2.850598647	Down
CYP4F11	2.752182163	UP	CTSD	-2.848780488	Down
TCEA1	2.751842219	UP	SCO2	-2.846560847	Down
ACPL2	2.749257279	UP	ZNF738	-2.845529412	Down
NGFR	2.745216516	UP	SLC25A23	-2.834173521	Down
RAD54B	2.742092457	UP	LOC401098	-2.831879241	Down

SLC46A3	2.741606715	UP	SNORD36A	-2.830530401	Down
C7orf47	2.741544352	UP	BCL11A	-2.830030488	Down
MRPS6	2.731483939	UP	LOC100133840	-2.829526369	Down
FAM50B	2.722602071	UP	LOC728620	-2.825449112	Down
HEBP1	2.716846637	UP	FOS	-2.822294023	Down
PRKAG2	2.715450469	UP	ZMAT3	-2.82052506	Down
AADAC	2.715101523	UP	P2RY5	-2.819918145	Down
LOC642590	2.713745246	UP	LOC400027	-2.818778726	Down
RFTN1	2.709574255	UP	GJB5	-2.813795337	Down
HMGA1	2.709194396	UP	LOC100190938	-2.811211585	Down
PPM2C	2.708400092	UP	MEIS2	-2.804358593	Down
NFIB	2.707155635	UP	RNF128	-2.804097311	Down
LOC730051	2.704405495	UP	OPN3	-2.803359684	Down
CGN	2.702812202	UP	PFKFB4	-2.803333333	Down
C6orf160	2.701793122	UP	MT1X	-2.780200071	Down
TRAF4	2.69968254	UP	MPPED2	-2.776714514	Down
BACE2	2.699099495	UP	SC65	-2.77535545	Down
SEMA4F	2.695075758	UP	PER3	-2.770967742	Down
ZNF395	2.689662888	UP	SLC25A28	-2.769572903	Down
PACS1	2.689243028	UP	RRM2	-2.765745441	Down
FTH1	2.688641665	UP	PRMT6	-2.764319915	Down
PPP2R2C	2.675613497	UP	TDRD1	-2.762951334	Down
ARL2	2.67538749	UP	FAM156B	-2.759605123	Down
TGFBR3	2.671325709	UP	SIPA1L2	-2.757724719	Down
UNC119	2.668918919	UP	SESN3	-2.745744097	Down
MRPL11	2.668682315	UP	FAM125A	-2.736174531	Down
VPS37C	2.661145194	UP	MAP3K6	-2.734833659	Down
LOC387820	2.658046479	UP	ZFAND2A	-2.730115891	Down
TMEM25	2.65473301	UP	ARL4A	-2.725944584	Down
LOC646347	2.651181941	UP	TP73L	-2.71652502	Down
RHOB	2.650734488	UP	LAMP3	-2.715429323	Down
DUS4L	2.646892655	UP	SPRR2F	-2.712177122	Down
XG	2.643338954	UP	CHMP1B	-2.710373444	Down
PCNXL3	2.633307869	UP	HIST3H2A	-2.708979903	Down
DIAPH3	2.632075472	UP	UNC5A	-2.706882591	Down
LHFP	2.628980078	UP	FLJ12684	-2.70652819	Down
GPX7	2.62836767	UP	ADAMTSL5	-2.706416809	Down
LOC440160	2.627680312	UP	SQRDL	-2.706098843	Down
DHRS13	2.625879805	UP	DMC1	-2.698365137	Down
KLHL29	2.618766999	UP	MFSD11	-2.687861272	Down
LOC645166	2.617225748	UP	GOLSYN	-2.68698102	Down
ANGPTL4	2.608878684	UP	NFIX	-2.685691426	Down
CYR61	2.603483129	UP	SRPK2	-2.684389911	Down
LOC729406	2.602830557	UP	C5orf13	-2.680191811	Down
ANO6	2.599588383	UP	FOXD1	-2.680182674	Down
CAPN1	2.594702206	UP	PLTP	-2.679984099	Down
IL1R2	2.593246753	UP	C9orf3	-2.678909091	Down
RTN3	2.58973117	UP	HIST1H3G	-2.672050562	Down
QPRT	2.588616715	UP	THOC5	-2.668293504	Down
PPM1M	2.586706647	UP	MBD4	-2.655213143	Down
C14orf2	2.582420361	UP	SFRS2B	-2.647386575	Down

FGD1	2.582271147	UP	LOC645553	-2.647381358	Down
MTSS1	2.581372549	UP	ATG16L1	-2.646644768	Down
NDRG3	2.579015544	UP	DEM1	-2.643334819	Down
ASAP1	2.578764247	UP	CDKN1A	-2.638809524	Down
FLNB	2.578399828	UP	LRRC8D	-2.635245001	Down
BANF1	2.578329198	UP	IGSF3	-2.62972947	Down
LRRC20	2.575236967	UP	IFITM1	-2.628149354	Down
KIAA1429	2.574066123	UP	PXDN	-2.620009857	Down
SCYL1	2.573726927	UP	HES2	-2.617486339	Down
SLC2A9	2.567317235	UP	HIST1H4K	-2.61404527	Down
SLC16A10	2.566593083	UP	C7orf40	-2.610342868	Down
NYNRIN	2.566433566	UP	ABCB6	-2.609402616	Down
SEL1L3	2.565530962	UP	CYB5A	-2.609341826	Down
DPF2	2.561096648	UP	LOC100131989	-2.607389304	Down
CCND1	2.558970436	UP	KEAP1	-2.602660172	Down
ZNF20	2.558754407	UP	LOC441087	-2.601675956	Down
KPNA3	2.557268457	UP	LOC729120	-2.600575172	Down
SNAP91	2.555290374	UP	PELI2	-2.595139607	Down
BEX2	2.554954955	UP	HPSE	-2.595011338	Down
CHES1	2.552858772	UP	SOX4	-2.592010478	Down
LOC401115	2.551702163	UP	P2RY1	-2.589900536	Down
BCAS4	2.550820616	UP	C1orf59	-2.585691889	Down
EXT2	2.549721037	UP	TIMM22	-2.583042973	Down
LMBR1	2.548737728	UP	CD2BP2	-2.580112828	Down
SHRM	2.545061283	UP	LEPREL1	-2.57799738	Down
C1orf19	2.544250282	UP	ZNF483	-2.575262544	Down
SLC44A2	2.543607889	UP	B9D2	-2.57366548	Down
LOC151162	2.54165592	UP	MCART1	-2.571477848	Down
ANGPT1	2.541273585	UP	SYNM	-2.561034275	Down
SYTL4	2.539931741	UP	STARD5	-2.559352518	Down
SUCLA2	2.539419087	UP	SLC6A10P	-2.559300874	Down
PEAR1	2.538787024	UP	HIST1H4H	-2.558202859	Down
TMEM171	2.538438528	UP	B3GALT6	-2.556751769	Down
GPX1	2.537588751	UP	NME3	-2.55496122	Down
OTUB2	2.535066505	UP	NPL	-2.5499114	Down
LOC100132992	2.53013213	UP	GBP2	-2.547689282	Down
TIAF1	2.529807439	UP	TMEM106A	-2.546037946	Down
MFSD3	2.526682395	UP	MCEE	-2.543227	Down
LTBP2	2.526568887	UP	KIAA0247	-2.542714957	Down
KCNS1	2.525801953	UP	CMPK2	-2.540832049	Down
PFKFB3	2.523507167	UP	LOC100130835	-2.538431449	Down
PACSIN3	2.522329767	UP	SLC36A1	-2.537397541	Down
OSGIN2	2.521441606	UP	EIF4E2	-2.534780923	Down
SSRP1	2.52126932	UP	DENND2C	-2.534237512	Down
LOC643949	2.517514377	UP	IP6K2	-2.532796781	Down
C4orf48	2.516650343	UP	FAM117B	-2.532719836	Down
CHST13	2.50977326	UP	TSHZ2	-2.525758645	Down
SORBS3	2.509256775	UP	LOC399900	-2.525586127	Down
WNT5B	2.507352941	UP	SREBF1	-2.523367379	Down
EHBP1L1	2.505394272	UP	LOC648526	-2.523065193	Down
C1QTNF1	2.504580691	UP	ARHGEF19	-2.518400727	Down

LOC644879	2.504174809	UP	ABCC3	-2.51780667	Down
HIPK2	2.500165071	UP	C21orf81	-2.516981132	Down
CRIPAK	2.497425335	UP	LOC643438	-2.51500664	Down
PATL1	2.49695439	UP	SPOCD1	-2.513546798	Down
EPAS1	2.495936061	UP	HLA-A	-2.509775566	Down
HSD17B11	2.495431894	UP	SETD6	-2.508524833	Down
LOC201651	2.494715984	UP	STK38	-2.5079122	Down
PPARG	2.494556765	UP	RP11-529I10.4	-2.50327291	Down
TSPAN6	2.492453389	UP	SPHK1	-2.502375297	Down
WTIP	2.490930369	UP	LHX2	-2.501531394	Down
CATSPER1	2.488464598	UP	DENND2D	-2.498722317	Down
LYPLA1	2.488249027	UP	DKFZp686O24166	-2.495622568	Down
CARHSP1	2.486741573	UP	GAMT	-2.494165694	Down
TSPAN9	2.485052803	UP	B4GALNT1	-2.492448759	Down
RNU1-5	2.483092876	UP	INPP1	-2.490467211	Down
ADAP2	2.480300188	UP	TMEM173	-2.488730867	Down
POPDC3	2.477009196	UP	SSFA2	-2.484727377	Down
ZNF358	2.474350649	UP	MANBA	-2.484487987	Down
FLJ32810	2.472261735	UP	FBXO4	-2.483475678	Down
UPK1B	2.471932115	UP	SERTAD1	-2.481487195	Down
KLF6	2.469614099	UP	AMMECR1	-2.480815348	Down
DRAP1	2.467051712	UP	IRF9	-2.480444214	Down
C8orf40	2.4638805	UP	SR140	-2.478819794	Down
GPR175	2.463011731	UP	PDIA3P	-2.477924277	Down
CENTG3	2.461511668	UP	LOC146909	-2.476718404	Down
TUB	2.460305899	UP	ALDH16A1	-2.476038339	Down
NELF	2.458347578	UP	DSC2	-2.469626168	Down
FUT8	2.456284957	UP	C1orf116	-2.468045693	Down
BAD	2.45563662	UP	FLJ44342	-2.466591166	Down
SDCBP	2.453761162	UP	TUBGCP5	-2.465646876	Down
DNER	2.453719008	UP	ELL2	-2.465047186	Down
EPHX2	2.451766685	UP	CBLB	-2.465001643	Down
RAB3IP	2.451223838	UP	IFIH1	-2.463843449	Down
PPP1R14B	2.451020798	UP	HNRNPU	-2.462156731	Down
ANG	2.449546183	UP	LOC100133583	-2.461945731	Down
TMEM9	2.446312178	UP	LOC100127975	-2.457703442	Down
MICB	2.445391304	UP	DUSP18	-2.457561728	Down
PSTPIP2	2.444230027	UP	P2RY11	-2.454356846	Down
XDH	2.441663131	UP	YWHAG	-2.448885943	Down
TAF1B	2.437886917	UP	NCK1	-2.446334509	Down
COG5	2.436563071	UP	STAT2	-2.44565695	Down
VEGFB	2.435026091	UP	CYP27C1	-2.442011834	Down
CHCHD6	2.434819714	UP	AGRN	-2.435938877	Down
PLEKHA6	2.426697531	UP	LOC100128510	-2.433841396	Down
LOC731777	2.426478642	UP	AKR1C3	-2.433623351	Down
FOXA1	2.423875887	UP	GTF2F1	-2.431738764	Down
LOC400879	2.423654568	UP	WDR75	-2.431429071	Down
LRCH2	2.423590504	UP	LOC645452	-2.430092391	Down
LOC643384	2.423343499	UP	ALKBH6	-2.428304904	Down
SMS	2.422753896	UP	KCNH6	-2.427350427	Down
MMP7	2.420414462	UP	OSCP1	-2.425972713	Down

TMEM14C	2.418768306	UP	MRPL54	-2.424521206	Down
VSTM1	2.417642467	UP	LOC100132394	-2.424121177	Down
EBPL	2.417384148	UP	LRRC37B2	-2.423261818	Down
FHL2	2.415430148	UP	SAMD9L	-2.422962015	Down
PHF3	2.415064103	UP	HPDL	-2.422249539	Down
DEFB103B	2.413092551	UP	LOC648921	-2.420837125	Down
MKLN1	2.411078359	UP	KIAA1522	-2.417529066	Down
ELFN2	2.407254362	UP	GCNT1	-2.413884473	Down
ETV6	2.407229596	UP	DNM1	-2.412611378	Down
C4orf14	2.402333459	UP	IFI30	-2.412551768	Down
ASB9	2.402016383	UP	COX19	-2.410899964	Down
OR56A3	2.3988006	UP	FLJ35390	-2.408161945	Down
FRMD4A	2.398460058	UP	RHBDD3	-2.407313541	Down
CMTM7	2.397887324	UP	CCDC3	-2.406388642	Down
AGPAT4	2.397449522	UP	CFD	-2.404456825	Down
MFSD10	2.397316857	UP	NUP188	-2.403753719	Down
TMEM55A	2.394969409	UP	SEMA3C	-2.401544402	Down
LOC729580	2.39428168	UP	C21orf24	-2.397696246	Down
NSFL1C	2.391406776	UP	SLCO3A1	-2.397528321	Down
C1R	2.388919978	UP	LOC88523	-2.395681354	Down
CDS1	2.388456091	UP	LOC730324	-2.393604422	Down
LOC730704	2.387659289	UP	SFXN1	-2.39033189	Down
SNHG5	2.387240497	UP	CHPF	-2.389688249	Down
NEO1	2.382451971	UP	TCTN1	-2.38875987	Down
TRAPPC2P1	2.380764563	UP	GJA3	-2.386262925	Down
VCAN	2.380442541	UP	CYP26A1	-2.385607604	Down
TXNRD1	2.378824433	UP	RHBDL2	-2.384201077	Down
NFKBIA	2.377898016	UP	ZNF18	-2.378767431	Down
CNN3	2.376730746	UP	LIG1	-2.377516779	Down
CD55	2.376581135	UP	GJA1	-2.375948532	Down
TMEM64	2.374604291	UP	GTF2I	-2.374615385	Down
EGLN3	2.373755126	UP	C16orf93	-2.37446403	Down
LOC645515	2.373726542	UP	LOC100134053	-2.371495327	Down
A4GALT	2.372406057	UP	CYCS	-2.370280736	Down
PCDHB2	2.37195122	UP	CBX2	-2.369230769	Down
ARHGEF5L	2.371801341	UP	SNX29	-2.36825886	Down
RPL23A	2.368048398	UP	DIAPH2	-2.366942149	Down
CA12	2.367637942	UP	BCL2L13	-2.366624146	Down
EIF2S2	2.366056096	UP	SGPP2	-2.36476256	Down
PRKCB	2.365853659	UP	SERPINB5	-2.362060259	Down
REPIN1	2.363781539	UP	OSGEP	-2.360957105	Down
RGS20	2.363702447	UP	NHLH2	-2.359208524	Down
ST3GAL6	2.357914812	UP	ABHD3	-2.358244201	Down
CDH13	2.3564753	UP	CRCP	-2.353901809	Down
SLC11A2	2.355969678	UP	SLC31A2	-2.351351351	Down
AGFG1	2.355909091	UP	TRMT1	-2.350564468	Down
TNPO3	2.354548484	UP	STAG3L1	-2.34869326	Down
DLD	2.351500704	UP	SPRR2A	-2.347720365	Down
CENPM	2.350784133	UP	HES5	-2.346079067	Down
CDKN3	2.348024151	UP	TMPRSS4	-2.344678218	Down
CHMP2B	2.347372045	UP	ZNF503	-2.341448931	Down

UBTD1	2.346659212	UP	MRRF	-2.335607597	Down
ZNF706	2.346500347	UP	TMEM154	-2.334667556	Down
MGC4677	2.345518157	UP	ZNF652	-2.334027055	Down
UNC93B1	2.342883267	UP	C1orf53	-2.327470493	Down
BMI1	2.341152754	UP	CCNH	-2.321834245	Down
LOC130773	2.339285714	UP	NUP62CL	-2.321034026	Down
C13orf37	2.335968044	UP	LOC647947	-2.320247934	Down
COPS6	2.334668609	UP	CFLAR	-2.316031812	Down
SRI	2.334464475	UP	DUXAP3	-2.315511727	Down
FAM62B	2.333270357	UP	TUFT1	-2.313880646	Down
RAB32	2.333269175	UP	JUND	-2.30447153	Down
WDR33	2.333110368	UP	EIF5A	-2.303941871	Down
RFNG	2.332723949	UP	PRKAB1	-2.30278129	Down
C1QTNF6	2.331360947	UP	SEC23B	-2.301012254	Down
SLC2A4RG	2.328908886	UP	MMACHC	-2.300131062	Down
AGBL5	2.326432022	UP	SCPEP1	-2.297436765	Down
GCAT	2.325570112	UP	SSR4	-2.295903829	Down
TESK1	2.323668639	UP	SULT1A1	-2.295518833	Down
LIFR	2.320430108	UP	TP63	-2.293746876	Down
TMEM97	2.319624901	UP	AMTN	-2.292152292	Down
C6orf129	2.316143121	UP	CLK1	-2.291309386	Down
KRT222	2.31501548	UP	C6orf141	-2.288584865	Down
HSPA1A	2.312598082	UP	EPHX1	-2.287408759	Down
FAM149B1	2.3125	UP	LOC729680	-2.287397541	Down
SCARA3	2.312425566	UP	PRDX2	-2.287339056	Down
CYB5R2	2.309906292	UP	LOC647389	-2.28706084	Down
FERMT2	2.308536585	UP	BTG2	-2.28622358	Down
EPHB4	2.307773109	UP	EVPL	-2.283615819	Down
DGKQ	2.306420852	UP	TNFRSF14	-2.283383686	Down
LAMB1	2.305790397	UP	HIST2H4A	-2.281708094	Down
FAM3C	2.304383366	UP	FXYD3	-2.27818448	Down
CXCL6	2.303981623	UP	CPEB1	-2.277127244	Down
DUSP1	2.303139824	UP	DPP7	-2.27643909	Down
SHROOM3	2.302250804	UP	NUPL2	-2.276263298	Down
POLA2	2.302244516	UP	LOC100130053	-2.274821109	Down
ELF3	2.300771605	UP	LOC284757	-2.274385408	Down
AQP11	2.3	UP	GLA	-2.273627315	Down
RWDD2A	2.297212653	UP	PCSK5	-2.270082114	Down
ZDHHC13	2.294947842	UP	IFI27L2	-2.269698671	Down
C9orf30	2.292950308	UP	KTELC1	-2.26889405	Down
WDSUB1	2.291907514	UP	SEC16A	-2.267822904	Down
ATP9A	2.290806476	UP	YBX2	-2.266814486	Down
LBR	2.29068941	UP	MX2	-2.265507941	Down
RAB11FIP5	2.288873371	UP	NGEF	-2.262536873	Down
FAM164A	2.286569718	UP	ZNF577	-2.260632072	Down
NICN1	2.286173633	UP	TAF1C	-2.258765432	Down
GLT8D2	2.28591649	UP	PSCD1	-2.256760282	Down
CTSZ	2.28402854	UP	CCNO	-2.25365761	Down
CCNI	2.282194095	UP	CCDC22	-2.251361868	Down
RCN1	2.281504065	UP	LEF1	-2.251124438	Down
LOC729231	2.279607163	UP	PDCD4	-2.249691739	Down

SERTAD4	2.277832903	UP	LOC100008588	-2.248120301	Down
UST	2.277628032	UP	ZNF558	-2.247398844	Down
ZFHX4	2.277145027	UP	MRPL46	-2.245793397	Down
IFI27L1	2.275850044	UP	PAX9	-2.245398773	Down
CDC42EP2	2.271322819	UP	PRSS12	-2.240930869	Down
JDP2	2.269411765	UP	COL8A2	-2.238897397	Down
ARHGAP22	2.267818959	UP	PGD	-2.233315276	Down
ZNF341	2.267422334	UP	FKBP15	-2.2331434	Down
WBP4	2.267075665	UP	PLIN5	-2.233064898	Down
CDC42EP5	2.266144814	UP	ALDH3A2	-2.232911104	Down
EMR1	2.264878458	UP	NPNT	-2.232498394	Down
UBASH3B	2.264014467	UP	NSUN4	-2.230610561	Down
RNU1-3	2.259160398	UP	GINS3	-2.229976876	Down
RNU1G2	2.256301406	UP	DKFZP564O0523	-2.229357798	Down
CCDC152	2.255796559	UP	FZD9	-2.229290921	Down
RNF24	2.255268199	UP	WDR55	-2.229239027	Down
RPL28	2.253946275	UP	TXLNA	-2.227996508	Down
VPS28	2.253671804	UP	PPA2	-2.227882772	Down
APOBEC3G	2.252368065	UP	DKFZP586I1420	-2.227158424	Down
FAM89B	2.250893921	UP	KLHL28	-2.22631935	Down
SMYD3	2.249885268	UP	SSTR2	-2.225547445	Down
LOC389599	2.249722531	UP	LOC644310	-2.224890414	Down
TMEM216	2.247912977	UP	ANKRD33	-2.223126089	Down
MAST4	2.246455098	UP	HIST1H2BG	-2.222470238	Down
ZNF318	2.243783304	UP	SFRS12	-2.222381123	Down
PCOLCE2	2.242988606	UP	PPP2R5A	-2.221916552	Down
MRPL49	2.241886919	UP	GALNS	-2.221392057	Down
HRAS	2.241714015	UP	DMTF1	-2.221023181	Down
PIGP	2.240862874	UP	SNORD36C	-2.220270765	Down
FAM189B	2.240711965	UP	HEY2	-2.217149907	Down
TACC3	2.239476357	UP	HIST1H3F	-2.216771249	Down
C12orf32	2.238624418	UP	TDP1	-2.213790772	Down
ADAM8	2.237798165	UP	MRPS30	-2.21320866	Down
NADSYN1	2.23623415	UP	CNNM1	-2.212717638	Down
ZDHHC5	2.235843281	UP	C9orf114	-2.211895911	Down
FAM111A	2.235677376	UP	ZNF486	-2.211345361	Down
SSPN	2.234375	UP	MDK	-2.210682493	Down
SSSCA1	2.233678902	UP	DTWD2	-2.210559006	Down
PGM2	2.233160622	UP	HERC6	-2.205149162	Down
NMU	2.232937473	UP	EXTL2	-2.204785077	Down
CBX1	2.231326392	UP	LTB4R2	-2.203067933	Down
SUV420H1	2.230444785	UP	ODF2L	-2.20142487	Down
SH3BGRL2	2.230430528	UP	KLF11	-2.201212121	Down
KCNG1	2.230082418	UP	FLJ41603	-2.199469496	Down
PABPC3	2.229793978	UP	CHRNA5	-2.197661898	Down
41528	2.229699912	UP	HOMER2	-2.197316496	Down
SASH1	2.228557345	UP	HERC2	-2.196531792	Down
AKTIP	2.227193745	UP	CENPV	-2.192782996	Down
CREG1	2.225219473	UP	IFNE	-2.192180617	Down
MTPN	2.224206845	UP	KLK6	-2.191268191	Down
NKX3-1	2.223274162	UP	SOCS2	-2.191152922	Down

SLC35D2	2.222068201	UP	DMRTA2	-2.1898017	Down
TWSG1	2.219672131	UP	HNRNPH2	-2.188486141	Down
ADRBK1	2.217837063	UP	NOL6	-2.187160857	Down
B4GALT5	2.217442919	UP	TCF25	-2.186798103	Down
SLC7A2	2.216256525	UP	LQK1	-2.185055866	Down
SPRY1	2.21442623	UP	RPS23	-2.185039714	Down
ARFGAP2	2.213912134	UP	UGCGL2	-2.184775087	Down
NEIL2	2.213307985	UP	GBP1	-2.184394741	Down
C11orf68	2.211760819	UP	CD68	-2.180535003	Down
TFAM	2.211584665	UP	TP53INP1	-2.179670085	Down
LOC643310	2.209221748	UP	PMS2L5	-2.178609062	Down
ABHD14A	2.207655502	UP	IQCK	-2.178402107	Down
CTNNAL1	2.206294086	UP	FLJ40722	-2.177785924	Down
C11orf84	2.205970149	UP	ACAT2	-2.177092139	Down
KLHL5	2.205956422	UP	MUM1L1	-2.176753783	Down
TMEM200A	2.204978038	UP	TUBB3	-2.176564399	Down
PHGDH	2.20292887	UP	NIPSNAP3A	-2.175623463	Down
WDR51A	2.202384531	UP	ZNF223	-2.175544233	Down
CDC25B	2.202081406	UP	LOC649639	-2.173774666	Down
CALD1	2.201780863	UP	LOC100128062	-2.170462197	Down
ZNF433	2.2	UP	XAF1	-2.170031881	Down
ENOPH1	2.199921555	UP	RIOK2	-2.168187001	Down
MAPK9	2.196002934	UP	RASEF	-2.168135095	Down
IFT20	2.193853974	UP	HISPPD2A	-2.165096678	Down
PNPLA2	2.193243734	UP	ERCC1	-2.164068037	Down
PI4K2B	2.19210084	UP	PIGW	-2.163678619	Down
VAT1	2.191926346	UP	CROT	-2.163140911	Down
RAPGEF3	2.191706731	UP	EHHADH	-2.162596401	Down
C1orf66	2.189897698	UP	H1FX	-2.159632124	Down
C7orf59	2.188658744	UP	KIAA0556	-2.15943945	Down
ACOX3	2.187410587	UP	CYTH1	-2.158547587	Down
APH1B	2.18715779	UP	FLJ44124	-2.158481691	Down
SLBP	2.186387199	UP	GSTO2	-2.156078148	Down
PXMP2	2.18490566	UP	HAS3	-2.155479712	Down
FAM10A7	2.183804987	UP	LOC644760	-2.154863655	Down
CCNC	2.181792319	UP	KLHL21	-2.154521175	Down
SLC12A9	2.181772575	UP	PPL	-2.154073709	Down
PCCA	2.181631455	UP	LOC440704	-2.153711201	Down
LOC729992	2.178544061	UP	RBM3	-2.151088842	Down
SIVA1	2.178465509	UP	PHTF1	-2.149106003	Down
ZNF583	2.178111588	UP	AK2	-2.146960371	Down
PCBD1	2.177754312	UP	ASAM	-2.146788991	Down
MT1M	2.177672956	UP	LRP8	-2.146783947	Down
MGLL	2.177272727	UP	PRO1853	-2.146596859	Down
FKBP1A	2.176531032	UP	UGT2B7	-2.146268657	Down
HMGB1L1	2.176438617	UP	MXD4	-2.145829283	Down
ENPP4	2.176208651	UP	CD109	-2.145056726	Down
PM20D2	2.170252824	UP	GSN	-2.144851658	Down
TSEN15	2.170192074	UP	TBC1D10A	-2.143555556	Down
CANT1	2.165660564	UP	BTN3A2	-2.143294238	Down
RAB1B	2.165012407	UP	FAM63A	-2.142737547	Down

BCL2L1	2.163058241	UP	PPP1R3F	-2.142679128	Down
KRT18P28	2.162636034	UP	RFX7	-2.141697522	Down
ALOX5AP	2.162316476	UP	LOC100132727	-2.141249207	Down
KIAA1324L	2.160968661	UP	CCDC88C	-2.141112047	Down
AKT1	2.15904936	UP	PPAP2C	-2.139261745	Down
PPAP2B	2.157320872	UP	FANCE	-2.139140063	Down
AP1S2	2.154988342	UP	KRT4	-2.13876652	Down
TRIB3	2.149921153	UP	LOC729090	-2.137207821	Down
TNIP1	2.149062192	UP	IFIT2	-2.137200737	Down
CBLL1	2.14849921	UP	PAM	-2.136819827	Down
SSH3	2.147658557	UP	LOC648024	-2.135743899	Down
GM2A	2.145547321	UP	COQ9	-2.135206024	Down
WASL	2.144735681	UP	ALPP	-2.133439886	Down
CHMP4C	2.144562334	UP	CEACAM6	-2.132895816	Down
ETS1	2.144491067	UP	LOC642956	-2.129475051	Down
TACSTD1	2.144380239	UP	LYSMD4	-2.129152542	Down
CD83	2.143021033	UP	SEMA4B	-2.12908396	Down
CPNE3	2.142635715	UP	TFDP2	-2.128937421	Down
ARFGAP3	2.141612813	UP	TYW1	-2.128289474	Down
LOC728758	2.141435306	UP	LOC646817	-2.125386518	Down
NT5C3L	2.140396773	UP	RICS	-2.125091263	Down
FAM10A4	2.134205529	UP	ZFP37	-2.122767857	Down
TMEM132A	2.133686758	UP	TNNT1	-2.120567376	Down
LYL1	2.130801688	UP	HINT3	-2.119456763	Down
SUSD3	2.129452055	UP	TAF15	-2.118365249	Down
TMEM165	2.128928571	UP	GIMAP8	-2.116245487	Down
SLC35A1	2.128345324	UP	HSCB	-2.115047352	Down
PRKCD	2.126531775	UP	FH	-2.11447718	Down
RPS19BP1	2.123197903	UP	LOC727768	-2.113924051	Down
NTAN1	2.122468295	UP	MYL5	-2.113553114	Down
APPL1	2.122184511	UP	DPYD	-2.113020833	Down
HSF2	2.121158242	UP	SURF6	-2.112949012	Down
SIVA	2.121072022	UP	C5orf44	-2.11284599	Down
CRY1	2.120345253	UP	CDKN2B	-2.11243851	Down
C11orf75	2.117963779	UP	ENSA	-2.112078845	Down
DTD1	2.117880795	UP	KCTD13	-2.111527648	Down
LOC728467	2.117845118	UP	NDUFAF1	-2.110917682	Down
CGGBP1	2.115700141	UP	LOC400578	-2.109614207	Down
POLR2L	2.115353038	UP	GPRIN1	-2.108628181	Down
DUSP4	2.114439324	UP	GPATCH2	-2.10796794	Down
SMARCE1	2.114221577	UP	POFUT1	-2.107610502	Down
TRIP10	2.113190992	UP	IL17RD	-2.107248834	Down
TPM1	2.113121476	UP	LOC647030	-2.10697938	Down
SLC30A9	2.112380557	UP	MAL	-2.1066961	Down
MTCH2	2.11223822	UP	FOXE1	-2.105653912	Down
MARCO	2.11159601	UP	SPINK5	-2.103719599	Down
TSPAN5	2.110364016	UP	ADCY7	-2.100287563	Down
CNTNAP1	2.109489051	UP	TSHZ3	-2.098393574	Down
RPL23AP7	2.109433962	UP	RABEP1	-2.096130774	Down
AOX1	2.105836576	UP	DHX58	-2.095581172	Down
ETNK2	2.105340519	UP	KIAA0513	-2.094919786	Down

ZNF419	2.104015027	UP	WDR13	-2.09375	Down
VIL2	2.103303141	UP	POLE3	-2.092977975	Down
PBX2	2.101409978	UP	KIAA1370	-2.091593065	Down
UBE2H	2.10128012	UP	ATP1B3	-2.091511023	Down
TNFAIP3	2.099450794	UP	TYW1B	-2.091344313	Down
PHF10	2.098796181	UP	FBXW7	-2.090909091	Down
PCDHB5	2.0982009	UP	KLF4	-2.090466531	Down
C1orf106	2.095314838	UP	MST1R	-2.090313685	Down
RAB17	2.094122167	UP	IGFBP6	-2.090246566	Down
RRAS	2.093649431	UP	RAPGEFL1	-2.089794667	Down
VCX	2.092307692	UP	PCSK7	-2.089738176	Down
C20orf27	2.092075642	UP	C3orf21	-2.089590276	Down
SAV1	2.09185632	UP	C14orf126	-2.089208152	Down
SYPL1	2.089767699	UP	WDR81	-2.087543554	Down
LOC441743	2.087972801	UP	MAPK13	-2.086681248	Down
RNF144B	2.087344029	UP	PRRX2	-2.08095952	Down
LOC392437	2.087152119	UP	PON2	-2.080551651	Down
EIF3F	2.086781313	UP	LOC100128353	-2.080344566	Down
PNMA2	2.085803432	UP	MRI1	-2.08026293	Down
GRSF1	2.085684861	UP	INTS7	-2.079182631	Down
INCENP	2.085604113	UP	LOC652184	-2.077490775	Down
C1orf115	2.085222121	UP	ST6GALNAC2	-2.075286609	Down
LOC653061	2.083816892	UP	HEY1	-2.074074074	Down
DPY19L1	2.083541927	UP	C9orf123	-2.073753815	Down
WARS2	2.083121827	UP	SLFN13	-2.073715562	Down
OVOL2	2.082546856	UP	CBX5	-2.073166304	Down
MAL2	2.079892734	UP	LEP	-2.071326676	Down
HMGN1	2.079744243	UP	LGALS8	-2.070048309	Down
C2CD2	2.07955657	UP	C19orf51	-2.069934641	Down
CDC2L6	2.079318605	UP	SLC6A8	-2.067532468	Down
STAU2	2.078244275	UP	TBCE	-2.067492604	Down
KLRAQ1	2.077368421	UP	MUM1	-2.06660105	Down
RHOG	2.07696878	UP	POR	-2.065668595	Down
LOC728060	2.075567162	UP	C1orf131	-2.065091732	Down
RHEB	2.075417216	UP	C2orf69	-2.063166169	Down
ADD1	2.070405795	UP	ANXA4	-2.062996294	Down
SIRPB2	2.069935112	UP	ABCA1	-2.062347188	Down
UBE2J1	2.069496089	UP	ZNF593	-2.062256109	Down
PISD	2.069064473	UP	FAM53B	-2.062186978	Down
ANKRD43	2.068990043	UP	TRAFD1	-2.061710746	Down
RYK	2.067895879	UP	GNB4	-2.058789625	Down
DLC1	2.067349138	UP	WBSCR22	-2.058586485	Down
RYBP	2.067079463	UP	LOC644852	-2.058441558	Down
C14orf149	2.066743955	UP	CEP27	-2.058267472	Down
SNX5	2.066358121	UP	PLXNB1	-2.058222222	Down
KCTD14	2.064470918	UP	BCAT2	-2.057910197	Down
LOC642489	2.064053796	UP	ATP6V1G1	-2.057816119	Down
CDK2AP2	2.062981298	UP	AHR	-2.054398664	Down
GPRASP2	2.061640588	UP	LOC100130276	-2.054054054	Down
CLDN1	2.061196033	UP	ESPN	-2.05390463	Down
GTPBP6	2.060575557	UP	ASCC2	-2.052690153	Down

S100P	2.05945122	UP	C14orf132	-2.049932524	Down
IBTK	2.058612975	UP	LOC650111	-2.049596774	Down
LOC653879	2.057736721	UP	TDG	-2.047974928	Down
SF3B2	2.057241353	UP	SLC25A46	-2.045140678	Down
HSD17B14	2.056122449	UP	STK36	-2.043613707	Down
TBC1D16	2.055955679	UP	TNRC15	-2.043434343	Down
CPSF1	2.055018972	UP	NUBPL	-2.042261731	Down
RAB11FIP1	2.054197662	UP	C3orf72	-2.041126761	Down
CRLF3	2.053881125	UP	DIP2B	-2.03876218	Down
ZNF343	2.053475936	UP	RARRES3	-2.03831563	Down
RAB28	2.0523918	UP	PPP1R10	-2.038216561	Down
FLOT2	2.051129804	UP	LUZP1	-2.037712736	Down
LRP5	2.050944669	UP	TTF1	-2.033535498	Down
PLEKHA2	2.048969943	UP	TMPRSS11D	-2.033215548	Down
WDYHV1	2.048749547	UP	DNAJC25	-2.032640286	Down
MAP3K11	2.044389642	UP	RBM34	-2.030534351	Down
SLC7A4	2.041948579	UP	MRPS2	-2.03038294	Down
LOC441455	2.041783708	UP	FABP5	-2.030380061	Down
ATF2	2.041741205	UP	ADAT1	-2.02964053	Down
TMEM33	2.041242363	UP	RANBP10	-2.022368904	Down
RPIA	2.040142591	UP	INPP5E	-2.022006714	Down
ZNF252	2.039070327	UP	PAQR4	-2.021255967	Down
ARMC10	2.037416089	UP	LOC440345	-2.019403691	Down
FRAG1	2.036386285	UP	GNAI1	-2.016571429	Down
LOC374395	2.035822752	UP	N4BP2	-2.015278025	Down
TRMT112	2.03567013	UP	LOC650803	-2.014279533	Down
SCCPDH	2.034175334	UP	POLR2A	-2.013204063	Down
TRIM6	2.033296643	UP	ZFPM1	-2.011206897	Down
GDPD5	2.032796318	UP	AIRE	-2.011014637	Down
CCDC136	2.032511211	UP	OAF	-2.009247312	Down
STEAP1	2.030059045	UP	PIP5K2B	-2.009119818	Down
LOC645251	2.027456027	UP	LOC652685	-2.00748183	Down
MYO18A	2.027231467	UP	NAGK	-2.006550934	Down
SNX3	2.025997761	UP	LOC100128505	-2.005098238	Down
FBXO32	2.025712025	UP	PALB2	-2.004024145	Down
SLC47A1	2.025586354	UP	FBXO31	-2.003966402	Down
BLMH	2.022887917	UP	LAMB3	-2.00392773	Down
PSIP1	2.021870118	UP	HIP1	-2.003452741	Down
CABLES2	2.021681416	UP	FLJ37078	-2.002909091	Down
CDC14B	2.021572387	UP	SNORA67	-2.00265903	Down
ZNF816A	2.020758123	UP	IL10	-2.002281566	Down
NCRNA00162	2.020689655	UP	ACAP2	-2.002111932	Down
CD14	2.018769552	UP	AKR1B10	-2.001746725	Down
CIB2	2.014848289	UP	LOC100130168	-1.997490694	Down
CCDC24	2.014568421	UP	CCDC146	-1.996953542	Down
SEC61A2	2.014111922	UP	RFX5	-1.995717005	Down
LOC284988	2.014008134	UP	S1PR5	-1.99552396	Down
TMCO3	2.013954131	UP	LOC647042	-1.99469496	Down
UNC84B	2.013771395	UP	CHMP4A	-1.993737455	Down
HOXC13	2.013143872	UP	C5orf28	-1.9932802	Down
MXRA7	2.009372747	UP	ZNF589	-1.993079585	Down

MED28	2.008926358	UP	CREB1	-1.992303026	Down
TRIOBP	2.008891213	UP	FGF11	-1.990425532	Down
LOC652672	2.008767952	UP	NAGPA	-1.984796855	Down
C17orf63	2.006673882	UP	GTF2B	-1.984040056	Down
ELOVL1	2.004896627	UP	SPSB1	-1.984033887	Down
ARMET	2.0022534	UP	C1orf63	-1.98314951	Down
SLC20A1	2.00091751	UP	LOC255326	-1.983080513	Down
GPR161	2.00071736	UP	LOC646123	-1.98069241	Down
CLCF1	2	UP	NRP1	-1.97837658	Down
CAPZA2	1.998100407	UP	MRPL34	-1.978365557	Down
MRPL15	1.997556008	UP	PARP9	-1.97793272	Down
TFAP2A	1.996667284	UP	LOC644250	-1.977931489	Down
LOC441481	1.996441281	UP	PES1	-1.977434235	Down
LOC729217	1.995048688	UP	WDR47	-1.976736345	Down
PRDX4	1.994964324	UP	LOC440957	-1.976555455	Down
NUAK1	1.994681764	UP	HOXD1	-1.975909306	Down
RDM1	1.993373827	UP	HLA-C	-1.975580937	Down
ASAH1	1.993330369	UP	MAP1LC3B	-1.975413311	Down
LOC392787	1.992748368	UP	IRF3	-1.975293	Down
CCDC86	1.992226314	UP	DAPP1	-1.971706999	Down
SLC2A12	1.991203958	UP	RTKN	-1.96984193	Down
ZFP82	1.99119171	UP	TBL2	-1.968703055	Down
PLAC1	1.990861619	UP	DOLPP1	-1.967540203	Down
LRP3	1.990585774	UP	DUOXA1	-1.966453674	Down
LOC646463	1.990544736	UP	LYPLAL1	-1.96582042	Down
MKRN1	1.989411137	UP	WDR19	-1.965562337	Down
AP4E1	1.987720837	UP	ACE2	-1.965206186	Down
SLCO4A1	1.987688098	UP	RNASEL	-1.963644731	Down
SYDE1	1.987124464	UP	STXBP3	-1.963266828	Down
COBL	1.987100737	UP	MC1R	-1.961930659	Down
CHRNB1	1.986634987	UP	CASK	-1.960433071	Down
MIR886	1.985533454	UP	FUK	-1.95972051	Down
LOC399942	1.985211927	UP	PMVK	-1.958798129	Down
FOXM1	1.984894773	UP	LOC202781	-1.958709813	Down
ESRRA	1.98401421	UP	S100A2	-1.956051568	Down
LEPROTL1	1.982360923	UP	REEP1	-1.954740538	Down
NUP54	1.98217089	UP	C9orf80	-1.954707783	Down
LRRC38	1.97948099	UP	CYP2J2	-1.954364361	Down
PPP1R3C	1.978915663	UP	MRPL20	-1.952422447	Down
LOC645381	1.978599222	UP	WDR12	-1.952021006	Down
VCX-C	1.978275181	UP	LOC728006	-1.950339971	Down
HSD17B2	1.977725674	UP	SPHK2	-1.948443922	Down
GNB2	1.977320675	UP	KIAA0495	-1.947239264	Down
GADD45B	1.977311209	UP	CHM	-1.947206166	Down
CNOT7	1.9766104	UP	ZNF271	-1.945850914	Down
CMIP	1.976343175	UP	IL17D	-1.945707071	Down
RELA	1.975718093	UP	HERC5	-1.944708451	Down
MICALL1	1.975280899	UP	LOC100134159	-1.944152184	Down
TGFBI	1.974819067	UP	MCL1	-1.942408807	Down
TMEM177	1.974615898	UP	HSPA4L	-1.941818182	Down
PIGU	1.97407274	UP	SFRS5	-1.94092505	Down

PDDC1	1.974016601	UP	COPZ2	-1.940768964	Down
ZMYND11	1.973629201	UP	N4BP1	-1.940319549	Down
ACTR1B	1.972701656	UP	MCM8	-1.939587828	Down
SNX15	1.968379447	UP	CNDP2	-1.938505999	Down
ITCH	1.967503218	UP	FEM1A	-1.938387194	Down
LOC100130561	1.967372742	UP	ARHGEF4	-1.937980241	Down
SPAG5	1.965401102	UP	FLJ42627	-1.937425507	Down
UPK2	1.964723926	UP	FLJ10916	-1.936932392	Down
NBPF20	1.964439192	UP	MPZL2	-1.936790449	Down
OSBPL5	1.96416185	UP	TRAPPC2L	-1.934824121	Down
MCFD2	1.9638135	UP	C17orf90	-1.934700407	Down
DEFB4	1.963549921	UP	WDR90	-1.934504792	Down
EDEM2	1.963528738	UP	ATG12	-1.934337998	Down
RAI14	1.963443834	UP	PSMC4	-1.93374613	Down
RHOQ	1.962074074	UP	PCSK9	-1.932874355	Down
NSMAF	1.96164309	UP	TXNDC17	-1.929452571	Down
NLK	1.961350511	UP	MEIS1	-1.928828469	Down
C9orf90	1.961055598	UP	CLSTN1	-1.927435041	Down
CTHRC1	1.960344183	UP	PHF14	-1.923920736	Down
PPP2CB	1.960327046	UP	LOC730990	-1.923876332	Down
ZDHHC24	1.958916084	UP	FAM174A	-1.923154362	Down
PPP1CA	1.957971105	UP	IFNE1	-1.92303339	Down
NELL2	1.95390625	UP	CCNE1	-1.922578445	Down
CRISPLD2	1.953513514	UP	HJURP	-1.922351462	Down
ZNF614	1.953410982	UP	HP1BP3	-1.921863799	Down
EIF5A2	1.953238789	UP	GALNT5	-1.921712538	Down
VEGFC	1.949617634	UP	BAK1	-1.92154195	Down
ANKRD6	1.949282787	UP	DDX19A	-1.920817669	Down
GRAMD1A	1.948698461	UP	GOLGA8B	-1.920297893	Down
MBNL2	1.947333772	UP	MOBKL2C	-1.920278393	Down
LOC728484	1.946637931	UP	PLDN	-1.919574104	Down
ITFG2	1.944837758	UP	LOC100128274	-1.919404517	Down
LOC642567	1.942696342	UP	CTSH	-1.91800771	Down
ADARB1	1.942485079	UP	LOC732075	-1.917900404	Down
LOC728931	1.941260745	UP	AP4B1	-1.917731111	Down
MAPK3	1.940659341	UP	TTC37	-1.916952857	Down
CCDC128	1.940365883	UP	SERPINE2	-1.916938189	Down
ZNF615	1.938271605	UP	SPRR2E	-1.915834522	Down
LOC100132863	1.937292208	UP	LMCD1	-1.915826912	Down
SBF1	1.937055477	UP	SNORD96A	-1.914923954	Down
FASTK	1.936893204	UP	MRPS21	-1.913228243	Down
KIAA0114	1.936740529	UP	ANKRD32	-1.911975435	Down
RIC8A	1.935730385	UP	GALNT14	-1.911743772	Down
OSTM1	1.934482759	UP	C10orf58	-1.91152	Down
RIMKLB	1.934289128	UP	YRDC	-1.911327918	Down
FAIM3	1.933993399	UP	CYLN2	-1.910768072	Down
MIF4GD	1.93364088	UP	THAP11	-1.909646739	Down
SAAL1	1.933495539	UP	MED27	-1.909379248	Down
RHOA	1.933122719	UP	SRP19	-1.909357653	Down
CEP135	1.932522334	UP	LOC654191	-1.909140969	Down
ACSS2	1.932076384	UP	HLA-E	-1.908670124	Down

CORO6	1.930177202	UP	FCAR	-1.908229048	Down
SUZ12	1.929825402	UP	LOC653489	-1.90739461	Down
SLC9A8	1.928758741	UP	ZNF430	-1.907192626	Down
PHLDA1	1.92808373	UP	ATP6V0A1	-1.906200583	Down
SAPS3	1.927748691	UP	TSR1	-1.906096504	Down
ARF5	1.926711084	UP	LOC653103	-1.905976301	Down
PCID2	1.926525748	UP	C9orf6	-1.904519569	Down
TMEM147	1.926515287	UP	LOC441089	-1.904257378	Down
BEX5	1.926489227	UP	SERBP1	-1.903650973	Down
RPL22L1	1.926353791	UP	TRIM29	-1.903225806	Down
AHNAK	1.926043292	UP	STK11IP	-1.902403846	Down
RANGAP1	1.925645439	UP	PSMD3	-1.901865831	Down
PLK2	1.92491839	UP	COTL1	-1.901194514	Down
ASXL1	1.923833891	UP	CRYAB	-1.900156006	Down
LOC651198	1.923799848	UP	FAM171A1	-1.899937067	Down
CTSL1	1.9232072	UP	NOMO2	-1.899838821	Down
PHC1	1.92195122	UP	PTGR2	-1.899668717	Down
DENND5A	1.921602045	UP	GPR177	-1.899664182	Down
SERGEF	1.921101899	UP	NT5DC2	-1.899264405	Down
IFRD2	1.920966152	UP	ATP1A1	-1.898924278	Down
AATF	1.920743127	UP	FAM172A	-1.897970085	Down
LOC643031	1.919141107	UP	HEATR3	-1.896296296	Down
TMEM184B	1.918989444	UP	C20orf177	-1.895605293	Down
QDPR	1.918878042	UP	DHRS4	-1.895365677	Down
PLOD2	1.918562874	UP	DCTN5	-1.894198935	Down
PACSIN2	1.918335296	UP	FAM156A	-1.894179894	Down
NAP1L1	1.917267669	UP	CD9	-1.891799737	Down
LOC389137	1.916899441	UP	MRP63	-1.889830508	Down
TXK	1.916859122	UP	ACADVL	-1.888750312	Down
CDCA5	1.915212618	UP	LOC100129195	-1.888497653	Down
CPOX	1.914968914	UP	GALNT3	-1.887651332	Down
EIF1AX	1.914625273	UP	ORC1L	-1.88717563	Down
ZFP64	1.914008882	UP	CYP27B1	-1.887125672	Down
MTMR12	1.913327882	UP	SPEN	-1.887092926	Down
ARSD	1.91254417	UP	FUT2	-1.886840432	Down
MBP	1.911640415	UP	CYCSL1	-1.886429295	Down
LOC649946	1.91138474	UP	LAMA3	-1.885215606	Down
FTHL3	1.910948905	UP	IFI16	-1.883761771	Down
PSMD11	1.909509375	UP	MFNG	-1.883016209	Down
FAM184A	1.909363296	UP	PPDPF	-1.880868956	Down
LOC646609	1.908790482	UP	MYOM2	-1.878998609	Down
CYP4V2	1.9084876	UP	MPHOSPH8	-1.878694158	Down
LYN	1.908219301	UP	ANKRD30B	-1.878050479	Down
PPIA	1.907540273	UP	ABCC1	-1.877277599	Down
LOC649169	1.907514451	UP	LOC728565	-1.876269036	Down
PGBD5	1.907335907	UP	MED31	-1.875079131	Down
ZNF626	1.907151819	UP	KRCC1	-1.874440895	Down
ST3GAL4	1.906410256	UP	C5orf21	-1.8744174	Down
SLC29A1	1.905468026	UP	JAG1	-1.873878628	Down
SNRNP200	1.905238095	UP	PLA2G4B	-1.873770265	Down
PPAT	1.904418487	UP	C8orf37	-1.873280157	Down

PECI	1.903082437	UP	NDEL1	-1.872494378	Down
LOC652846	1.90297069	UP	GPR37	-1.871312309	Down
C1orf21	1.901851852	UP	LYPD1	-1.87006052	Down
BHLHB2	1.90155351	UP	IQCG	-1.86959099	Down
RAB22A	1.901535852	UP	ARID5B	-1.869335501	Down
C21orf56	1.901515152	UP	NEFL	-1.868547832	Down
ARNT2	1.901315789	UP	TOP3B	-1.868214717	Down
FLJ43681	1.900862902	UP	LOC653994	-1.868155485	Down
C9orf127	1.900290416	UP	SNAPC5	-1.867864271	Down
PTPN20	1.900221729	UP	SRM	-1.86706547	Down
LRTOMT	1.899645809	UP	STOX2	-1.866117719	Down
SERINC2	1.89874507	UP	TMEM60	-1.866089423	Down
POLDIP2	1.898645258	UP	FASTKD1	-1.865822785	Down
KRT18P17	1.896630497	UP	LOC100134189	-1.865616798	Down
41339	1.896577708	UP	HSH2D	-1.86537077	Down
CCDC25	1.895760234	UP	LOC731542	-1.864935419	Down
TMEM199	1.895711144	UP	BTN3A3	-1.864864865	Down
LOC731915	1.894906166	UP	CCRK	-1.864647421	Down
HIST1H4C	1.893470666	UP	TNFRSF12A	-1.864455095	Down
DAPK1	1.892035398	UP	CCDC130	-1.863623061	Down
SBF2	1.891378471	UP	C10orf54	-1.863444912	Down
ASS1	1.891311318	UP	BCKDHA	-1.863333333	Down
SDF2	1.891261763	UP	EIF4H	-1.863264652	Down
BRD9	1.891088386	UP	LOC647281	-1.863073562	Down
TMEM166	1.890692954	UP	FURIN	-1.862814292	Down
LOC100216001	1.890424987	UP	EFHC1	-1.86281407	Down
TRIM8	1.89025028	UP	C1orf51	-1.86259542	Down
CKAP2L	1.889010645	UP	MTERF	-1.861975117	Down
KRT8P9	1.888716356	UP	CCDC109A	-1.860482944	Down
PTPN11	1.888114156	UP	THOP1	-1.86023859	Down
IPO7	1.88748019	UP	BBS4	-1.859581882	Down
PIN4	1.887384949	UP	AHSA2	-1.859455655	Down
TSPAN17	1.885924728	UP	NDUFA7	-1.858989153	Down
OTUB1	1.885819521	UP	SFN	-1.858496214	Down
NUDT9	1.884927066	UP	ALDH4A1	-1.857972545	Down
NBR2	1.884595024	UP	MIR1978	-1.857323601	Down
FRS3	1.882895343	UP	FLAD1	-1.856812552	Down
CLP1	1.882711672	UP	FLJ25363	-1.856781027	Down
HNRNPUL2	1.882186082	UP	LZTS2	-1.855457227	Down
TRIP13	1.881285187	UP	TNFRSF25	-1.85521431	Down
AKAP12	1.880984043	UP	SNORD12C	-1.855018587	Down
STAU1	1.880227218	UP	PPWD1	-1.854695892	Down
LOC730012	1.880126183	UP	KLK8	-1.8543379	Down
IER3	1.880123733	UP	TM4SF1	-1.854313725	Down
SDC4	1.878993662	UP	TTC4	-1.854061857	Down
MTERFD1	1.878597845	UP	TICAM2	-1.851987024	Down
ANTXR1	1.877530864	UP	ACYP2	-1.851595745	Down
RASSF7	1.876459321	UP	KRT19	-1.851450446	Down
ADAM10	1.87631935	UP	LOC646996	-1.849992017	Down
ATXN10	1.876190476	UP	LOC100130445	-1.849906606	Down
NEK6	1.876064096	UP	MID1IP1	-1.84990592	Down

SPTLC3	1.875191424	UP	TUG1	-1.849108218	Down
MCOLN3	1.873992977	UP	MRPS10	-1.848817898	Down
VPS37A	1.873841768	UP	SPG3A	-1.848707309	Down
CXXC5	1.873143316	UP	SNORD49A	-1.848303393	Down
DNAL4	1.87300922	UP	RAB2B	-1.848194224	Down
LOC727865	1.872922097	UP	RPA2	-1.84767228	Down
FVT1	1.872746554	UP	DDX58	-1.845989305	Down
KCTD6	1.872505918	UP	DUSP23	-1.845942552	Down
GBAS	1.872466216	UP	NP	-1.84581703	Down
PIGS	1.872400542	UP	AGA	-1.845149731	Down
CREB3L2	1.871705544	UP	EFHA1	-1.84439155	Down
ERAL1	1.871333712	UP	LOC286208	-1.843897328	Down
C20orf20	1.87115648	UP	C9orf69	-1.843763273	Down
SOD1	1.870620084	UP	PDSS1	-1.843383585	Down
WWC3	1.870198343	UP	CXCL14	-1.842105263	Down
SLC25A22	1.870185449	UP	MRPL50	-1.841746023	Down
ARID3B	1.870135028	UP	AES	-1.841483516	Down
RAB5C	1.869906118	UP	ERMP1	-1.840742037	Down
VAMP4	1.869424883	UP	LRP1B	-1.838639653	Down
C10orf33	1.869085174	UP	INO80D	-1.838514443	Down
LOC653297	1.86815562	UP	RAPGEF5	-1.837959374	Down
CYB5R4	1.868096647	UP	POLR3E	-1.837416481	Down
ANXA8L1	1.867704671	UP	LMLN	-1.83701832	Down
HRIHFB2122	1.867647059	UP	DHX38	-1.836990596	Down
ATG5	1.867524339	UP	SLC12A2	-1.836808451	Down
ZNF347	1.867517957	UP	C6orf85	-1.836722368	Down
ARMCX1	1.867102397	UP	WDR36	-1.835616438	Down
MPP5	1.867007277	UP	NQO2	-1.834872979	Down
LOC653778	1.866856871	UP	H1F0	-1.834720323	Down
TAGLN2	1.864241108	UP	ZBTB33	-1.833631978	Down
SNORD13	1.864122137	UP	TRIM2	-1.832544379	Down
TCHH	1.862967157	UP	IL18R1	-1.8325	Down
TINAGL1	1.862120405	UP	C21orf55	-1.831462823	Down
ALG9	1.861985889	UP	PSMB2	-1.831056309	Down
VTI1B	1.861303745	UP	SNORD16	-1.830849104	Down
GDF15	1.860962567	UP	TMEM208	-1.830471715	Down
PTP4A1	1.860525462	UP	FAM81A	-1.829831933	Down
OOEP	1.860402685	UP	ZFYVE26	-1.82962963	Down
VAMP5	1.859216075	UP	HEATR5A	-1.828764045	Down
RCE1	1.858243451	UP	RNF7	-1.828736877	Down
ACAA2	1.858138581	UP	RIPK1	-1.828222882	Down
DYNLRB1	1.857891629	UP	PRKAB2	-1.827806864	Down
LOC730455	1.857821191	UP	LOC653583	-1.827212523	Down
SNCG	1.857142857	UP	LOC146517	-1.827193256	Down
GNAI2	1.856595295	UP	LOC100008589	-1.826526131	Down
HOXA5	1.856175973	UP	GOLPH3L	-1.825303077	Down
TMEM115	1.855767548	UP	EIF4G3	-1.825202788	Down
LOC653752	1.855106888	UP	SLC7A11	-1.824900512	Down
ABCB10	1.854225752	UP	POLR3G	-1.82471008	Down
ZFPL1	1.853875476	UP	C16orf68	-1.821640904	Down
FER1L4	1.853708791	UP	SIL1	-1.821080602	Down

CHMP4B	1.853662106	UP	MNT	-1.819136523	Down
MPRIP	1.853447239	UP	HSPC111	-1.816359491	Down
KIAA0040	1.853381518	UP	PNKP	-1.815414964	Down
PMPCB	1.853285112	UP	MPHOSPH10	-1.815411255	Down
KYNU	1.852943201	UP	KDM5B	-1.813867385	Down
ZMIZ2	1.852140078	UP	PCDHB9	-1.813781073	Down
ATG2A	1.851927496	UP	LOC347376	-1.813547954	Down
NLGN2	1.851851852	UP	LOC647886	-1.812537493	Down
ERO1L	1.851136005	UP	C9orf116	-1.812444047	Down
MRPS28	1.850797623	UP	LRRC8C	-1.812207944	Down
TEX264	1.850187266	UP	ZFP36	-1.812183137	Down
C7orf10	1.849857007	UP	DCTPP1	-1.812046469	Down
LOC644058	1.849819495	UP	MRPL32	-1.811876289	Down
CALHM2	1.84965035	UP	EHD4	-1.810778947	Down
ABTB1	1.84824518	UP	ANKRD57	-1.810562414	Down
SLC35B4	1.848	UP	MED26	-1.808170515	Down
LOC283392	1.84746922	UP	ENDOD1	-1.808059701	Down
UNC50	1.847350133	UP	XPNPEP3	-1.807671891	Down
SLC35F5	1.847119224	UP	COMMD4	-1.80733945	Down
B3GAT3	1.844423618	UP	LOC653377	-1.807222324	Down
CTNNBIP1	1.843537415	UP	LOC728499	-1.806683323	Down
ANXA8	1.8434238	UP	SDCCAG3	-1.806555004	Down
RBM47	1.842956853	UP	ARMC5	-1.806109726	Down
ASAP3	1.842885375	UP	CEP164	-1.805809789	Down
LOC728975	1.840096911	UP	DDX56	-1.805617909	Down
DDB1	1.838951311	UP	TP53BP1	-1.805372884	Down
DOK7	1.838859802	UP	DNAJC17	-1.804832714	Down
BCAR3	1.8384867	UP	AGPAT2	-1.804457601	Down
PLAGL2	1.838265836	UP	TRPC1	-1.804390564	Down
FLI1	1.835972851	UP	CASP1	-1.804171729	Down
TRAM1	1.835322332	UP	ZZZ3	-1.803940089	Down
CYB5R3	1.834631969	UP	LILRB1	-1.803480715	Down
CLPTM1	1.832945837	UP	ATP2B4	-1.803428959	Down
RP1L1	1.832782551	UP	NOMO1	-1.803358522	Down
LETM1	1.83248731	UP	LOC728888	-1.803122807	Down
MKI67	1.832095945	UP	POU3F1	-1.802735781	Down
GABBR2	1.831232493	UP	FAHD1	-1.8026338	Down
IL13RA1	1.830902587	UP	RAXL1	-1.801987968	Down
HSBP1	1.83032657	UP	S100A3	-1.801109661	Down
TM4SF19	1.830249769	UP	ST6GAL1	-1.799906279	Down
PC	1.829751131	UP	C12orf26	-1.799729973	Down
ATP7B	1.829407566	UP	DOLK	-1.798807543	Down
MAEA	1.82915679	UP	GPER	-1.796244784	Down
TNFRSF1A	1.82912825	UP	VAV2	-1.795898903	Down
PAX6	1.828103683	UP	SNAPC4	-1.795819477	Down
PPP2R5C	1.827215756	UP	LOC100131541	-1.794871795	Down
SNCAIP	1.826979472	UP	POM121C	-1.792510942	Down
C2orf65	1.826371826	UP	LOC387934	-1.792367788	Down
LOC100130633	1.826309293	UP	LOC347292	-1.792252511	Down
TXNDC14	1.825904328	UP	RABGAP1L	-1.791737649	Down
JAG2	1.825648855	UP	ISG20L2	-1.791554642	Down

dJ341D10.1	1.82527008	UP	PCF11	-1.790636813	Down
LOC390466	1.82441202	UP	LOC346887	-1.790601244	Down
FAM107B	1.823282812	UP	LOC649722	-1.7896735	Down
IMPAD1	1.822707081	UP	ASNSD1	-1.7891029	Down
CUEDC1	1.822151011	UP	MED29	-1.789015405	Down
LOC730525	1.822125813	UP	STXBP2	-1.788448845	Down
SNRPB	1.821184425	UP	SMARCA1	-1.787241379	Down
GPR126	1.821052632	UP	GRN	-1.786883288	Down
CHAF1B	1.82101463	UP	ENTPD7	-1.786199095	Down
SLC46A1	1.820477816	UP	IDS	-1.786098874	Down
CGRRF1	1.820321255	UP	FAM36A	-1.785978771	Down
FAM181B	1.820139329	UP	MFSD6	-1.785757032	Down
SRP72	1.820101966	UP	TSPAN1	-1.784515749	Down
ZNF777	1.818181818	UP	TRMT12	-1.784452297	Down
SFMBT1	1.817851959	UP	C1orf109	-1.784288474	Down
PRDX3	1.817598145	UP	U2AF2	-1.784240244	Down
EIF3L	1.817099372	UP	LOC644404	-1.784005038	Down
FAM105A	1.815816154	UP	SLC6A15	-1.783408255	Down
LMBRD1	1.815785055	UP	SNORA24	-1.783390411	Down
DKK3	1.814913105	UP	C7orf42	-1.78305904	Down
CTPS2	1.814792723	UP	PDE4C	-1.782251774	Down
GPR81	1.814339623	UP	ARPM1	-1.781747948	Down
MPDZ	1.81365254	UP	DHRS7B	-1.781384435	Down
TYRO3	1.81356383	UP	UCRC	-1.781062356	Down
CAMK2N1	1.812690075	UP	BSG	-1.780306699	Down
PAAF1	1.812094294	UP	SHCBP1	-1.77995544	Down
ZNF337	1.811531704	UP	F12	-1.779663518	Down
ILK	1.810519167	UP	LOC440353	-1.77875047	Down
B3GALTL	1.809954751	UP	TNFSF14	-1.778619756	Down
ABHD7	1.809936018	UP	PPIG	-1.777815239	Down
ZYG11B	1.808095586	UP	COX7B	-1.777135146	Down
TMEM125	1.807210816	UP	SPTBN1	-1.776363636	Down
UBQLN4	1.807017544	UP	BAZ1B	-1.776240209	Down
MAN1A1	1.806650246	UP	LRRC28	-1.776192264	Down
RNU1A3	1.806437486	UP	LOC153561	-1.775269872	Down
CNPY3	1.806131816	UP	TOR1B	-1.775178026	Down
DIS3L	1.805807114	UP	KLHL24	-1.775136206	Down
LOC283267	1.805593555	UP	EFNB2	-1.774897481	Down
EIF3D	1.804516892	UP	CSNK1A1	-1.774665703	Down
FARP1	1.804416404	UP	NUDC	-1.774142574	Down
TSG101	1.803791252	UP	ERI2	-1.773938796	Down
RNFT2	1.803239352	UP	CCDC21	-1.77391525	Down
TFPI2	1.802276812	UP	ELMO3	-1.773824981	Down
PKP4	1.802071154	UP	TNFAIP8	-1.773218143	Down
LRPAP1	1.80174162	UP	CDK5RAP3	-1.773005369	Down
GRPEL1	1.799903607	UP	SLC16A3	-1.772023204	Down
DCUN1D4	1.799001248	UP	C16orf5	-1.771577381	Down
ERLIN1	1.795949214	UP	TMEM49	-1.771270953	Down
LOC340598	1.794484912	UP	ANKRD11	-1.771232685	Down
RNF39	1.793150685	UP	LOC644914	-1.770595835	Down
WFS1	1.791302448	UP	FABP5L2	-1.769763694	Down

CSAG3	1.790635452	UP	CHMP1A	-1.76812606	Down
USP6NL	1.790584416	UP	FLJ38717	-1.767304189	Down
PSMG4	1.790259559	UP	CDT1	-1.767253045	Down
MRPS33	1.78809994	UP	CCDC14	-1.766489635	Down
RBM12B	1.787804878	UP	FAM73B	-1.76609724	Down
PTPMT1	1.787301972	UP	MFSD1	-1.765747126	Down
BRWD1	1.787080103	UP	RNASEH2A	-1.765368749	Down
EEF1G	1.786851418	UP	ZMIZ1	-1.763978002	Down
PPP2R2A	1.786044743	UP	TCN2	-1.763809524	Down
KBTBD9	1.784234753	UP	LOC100130598	-1.76285461	Down
LIMS2	1.782547502	UP	CEP110	-1.762849162	Down
MGC3032	1.781917808	UP	KIAA1012	-1.762528776	Down
C11orf2	1.78165075	UP	EWSR1	-1.762275775	Down
CD276	1.781600781	UP	ATM	-1.761339846	Down
LPCAT3	1.780753518	UP	BAIAP2	-1.76029654	Down
LOC100129144	1.780658725	UP	CELSR2	-1.759200363	Down
LY6E	1.780517401	UP	PNPT1	-1.758787388	Down
FNTB	1.780104712	UP	TAP1	-1.758496815	Down
ZNF329	1.779932287	UP	CD70	-1.758313539	Down
LOC729009	1.779516594	UP	COL27A1	-1.758166969	Down
ZNF533	1.779464931	UP	METTL11A	-1.757355988	Down
RPUSD3	1.779403489	UP	CCNL1	-1.757131432	Down
TRIM44	1.778397376	UP	FBXL16	-1.756939235	Down
GANAB	1.777744041	UP	IPO13	-1.756455816	Down
C7orf49	1.777222096	UP	C14orf79	-1.756132243	Down
CAV2	1.776787965	UP	LOC100133923	-1.755867114	Down
TBPL1	1.776725399	UP	STAT6	-1.755660696	Down
TMEM8	1.775979863	UP	LOC653158	-1.755227552	Down
ZNF559	1.775832475	UP	LOC649841	-1.753569212	Down
KIF13B	1.775492716	UP	LOC732360	-1.753393012	Down
SRGAP1	1.775069423	UP	THG1L	-1.753183154	Down
COL5A1	1.775035692	UP	MAOB	-1.752775722	Down
CHN1	1.77460556	UP	FETUB	-1.752695902	Down
BST1	1.773940345	UP	SUOX	-1.751135626	Down
HLA-DMA	1.77388724	UP	PSMB8	-1.751072961	Down
SMAP1	1.773562883	UP	HSPC171	-1.75089222	Down
GYG2	1.773224044	UP	COL4A6	-1.750868056	Down
GSDMC	1.773166927	UP	ARPC5L	-1.749604346	Down
BRI3	1.772951594	UP	AMACR	-1.749075975	Down
EIF4ENIF1	1.772773797	UP	CLCA4	-1.749022674	Down
PNPLA4	1.772352941	UP	NPAL3	-1.748719262	Down
MIR614	1.770524899	UP	PITX1	-1.748589209	Down
DUSP14	1.770103233	UP	GLMN	-1.748145095	Down
SPAG1	1.769721116	UP	ANKH	-1.747368421	Down
CKAP5	1.76954498	UP	NCBP2	-1.747334675	Down
SNX14	1.769524294	UP	TUBA4A	-1.746464964	Down
LOC730382	1.769351056	UP	ESYT1	-1.746398803	Down
LOC387703	1.768939864	UP	GUSBL1	-1.745771144	Down
LOC728362	1.768711656	UP	CES2	-1.745058626	Down
PQLC1	1.76795017	UP	TANC2	-1.745028603	Down
ANKRD54	1.767372502	UP	SDCBP2	-1.744722955	Down

FYN	1.765832106	UP	TTLL12	-1.744472681	Down
USP46	1.765765766	UP	CCDC112	-1.743733794	Down
KLC1	1.764863957	UP	ZSWIM7	-1.74278648	Down
GSDM1	1.762723521	UP	GUSB	-1.742172725	Down
C11orf10	1.762246127	UP	C19orf23	-1.74011713	Down
ZNF788	1.761549925	UP	C9orf119	-1.738801085	Down
UEVLD	1.761286682	UP	PRICKLE1	-1.738675958	Down
CDCA7L	1.760801211	UP	HACL1	-1.738304771	Down
LYAR	1.760690416	UP	GABRE	-1.737736765	Down
MPST	1.760522496	UP	KRT24	-1.737704918	Down
H2AFV	1.7599182	UP	MAFB	-1.737288136	Down
RWDD1	1.759604758	UP	B3GALNT2	-1.737116765	Down
TNIP2	1.758093205	UP	CCDC16	-1.736236306	Down
YES1	1.757859966	UP	CSF2RA	-1.73542687	Down
C15orf48	1.757181572	UP	LOC100134073	-1.735273973	Down
IFT52	1.756962628	UP	C3orf16	-1.733855186	Down
MRPL48	1.75539318	UP	C16orf33	-1.733285509	Down
MARCKS	1.754964336	UP	LIMK2	-1.733091787	Down
TBC1D14	1.754913671	UP	C18orf56	-1.732666667	Down
GFI1	1.754649499	UP	GGPS1	-1.732559869	Down
TMEM106C	1.754525762	UP	RALGDS	-1.732003291	Down
WWP1	1.754518887	UP	RGS2	-1.731543624	Down
CLCN4	1.754050074	UP	SLC7A8	-1.731182796	Down
PVR	1.75327291	UP	C1orf212	-1.730929265	Down
PLSCR4	1.752347038	UP	LOC652755	-1.729002625	Down
ST7	1.752104771	UP	DBT	-1.728996639	Down
AKR1B1	1.751893673	UP	LOC728059	-1.728884678	Down
IFRG15	1.751798561	UP	LOC400890	-1.727832842	Down
FAM45A	1.751713167	UP	KIAA0368	-1.727774241	Down
ANXA11	1.751441638	UP	C10orf125	-1.726784344	Down
RAB8B	1.750923873	UP	LOC642299	-1.726781493	Down
NFKBIE	1.74969574	UP	LOC652657	-1.726435536	Down
GATA2	1.749450549	UP	TCFL5	-1.726030369	Down
DNAJC4	1.749446658	UP	LOC100190986	-1.725952227	Down
PRPS2	1.749352911	UP	RAB36	-1.725761773	Down
C20orf111	1.74884992	UP	FRAT2	-1.725059466	Down
HMGB2	1.748836006	UP	TMEM87A	-1.724953445	Down
PDIA5	1.748732008	UP	LRMP	-1.723418573	Down
RP9	1.747601396	UP	MARK1	-1.723329049	Down
MAP7	1.746732197	UP	LOC100134364	-1.723242022	Down
RGS17	1.74650913	UP	SPRR2C	-1.722983257	Down
FIBP	1.746362147	UP	STAC	-1.722417428	Down
GLO1	1.745565352	UP	ALPL	-1.722254503	Down
FLII	1.745202694	UP	IFI27	-1.721302566	Down
MTHFD1L	1.745001538	UP	LOC100127982	-1.720565632	Down
LOC651524	1.744892325	UP	FLJ35934	-1.720422535	Down
PTOV1	1.74488568	UP	LOC642333	-1.720229555	Down
ZNF671	1.743478261	UP	DYNC2LI1	-1.720215857	Down
LOC647436	1.743238236	UP	TYK2	-1.719981978	Down
FBXO7	1.7431215	UP	PUS3	-1.719662752	Down
CXCL2	1.742807825	UP	C1orf55	-1.719560645	Down

PLCB4	1.741534209	UP	KLK10	-1.719536757	Down
PDIA4	1.741333333	UP	PRSS23	-1.719286075	Down
ITGB4BP	1.740939375	UP	SH3BGRL3	-1.718846988	Down
CYBASC3	1.739121676	UP	LRRCC1	-1.718778487	Down
EZR	1.738996636	UP	KRT6A	-1.718477324	Down
ZNF134	1.73819978	UP	PID1	-1.717376134	Down
ZNF746	1.737955347	UP	PRPF4	-1.715996152	Down
ARIH2	1.737265068	UP	LOC400446	-1.715164616	Down
LOC401397	1.736935373	UP	LOC729082	-1.714624238	Down
RNASEH2B	1.73671875	UP	ZNF860	-1.714364947	Down
PPM1H	1.736370426	UP	C9orf23	-1.714070446	Down
GOLM1	1.736234458	UP	EDAR	-1.714050494	Down
PRMT2	1.736189402	UP	RAB30	-1.714048673	Down
SLC25A15	1.736013614	UP	SLC5A8	-1.71336314	Down
ZNF285A	1.735858848	UP	WDR40A	-1.713352389	Down
PODXL	1.73526616	UP	RSL1D1	-1.713270227	Down
TSC22D4	1.735169492	UP	SFRS7	-1.713203068	Down
GAK	1.734854059	UP	WBSCR16	-1.713031423	Down
CD81	1.734413632	UP	SLC16A12	-1.71250025	Down
RAMP1	1.734317343	UP	MRPL40	-1.712393888	Down
FAM70A	1.73318872	UP	CPE	-1.712239583	Down
DHRS7	1.733032039	UP	LAGE3	-1.711884205	Down
WDR74	1.73253406	UP	LOC440348	-1.7118074	Down
RNH1	1.731787727	UP	EPM2AIP1	-1.711580801	Down
MYL9	1.731034483	UP	MPV17	-1.711431227	Down
ICMT	1.730670927	UP	DUSP5P	-1.711009174	Down
TGFBR2	1.73066619	UP	TFCP2L1	-1.710830705	Down
LOX	1.730369515	UP	LOC650128	-1.710372535	Down
SLC41A2	1.729941292	UP	FAM18B	-1.710337553	Down
PIK3CB	1.7291884	UP	SP100	-1.709531014	Down
FAH	1.728457499	UP	IGFBP3	-1.709234066	Down
SH2D4A	1.727156713	UP	TYW3	-1.709017497	Down
IL6	1.72677761	UP	ZBTB48	-1.708878183	Down
NAP1L5	1.726086957	UP	PPP1R14C	-1.708622269	Down
TMEM40	1.725607328	UP	LOC100128126	-1.708098988	Down
SLC35E3	1.725420348	UP	LOC392713	-1.707573633	Down
LOC440145	1.724572004	UP	ZFP3	-1.70726339	Down
CFL1	1.724173816	UP	STYXL1	-1.70716448	Down
LOC729273	1.723913043	UP	LOC389517	-1.706511001	Down
JPH2	1.723910841	UP	TMTC3	-1.706507304	Down
PLCXD1	1.723868596	UP	HARS	-1.705868613	Down
WDR1	1.722759574	UP	TATDN3	-1.705295736	Down
AP1S1	1.721967814	UP	C18orf55	-1.704818804	Down
PLCG1	1.721835075	UP	GLS	-1.704754884	Down
FLYWCH2	1.721715328	UP	LOC730740	-1.704191703	Down
MGC16121	1.721693469	UP	LOC653086	-1.704142923	Down
CUL1	1.721328312	UP	ZNF770	-1.704060385	Down
THEX1	1.720504732	UP	LOC728698	-1.703794279	Down
RAB2A	1.720347072	UP	ANKRD41	-1.703597122	Down
SKP2	1.720268841	UP	LOC653829	-1.703506908	Down
TAF4	1.719794344	UP	C19orf24	-1.703321879	Down

CRLS1	1.719736375	UP	BSDC1	-1.702552389	Down
PAIP2	1.717674806	UP	TNFSF15	-1.702215353	Down
SLC48A1	1.717670286	UP	ZRANB2	-1.701997634	Down
ZNHIT1	1.717397356	UP	C15orf57	-1.701953125	Down
CSAG3A	1.717192982	UP	NAPA	-1.701602959	Down
SND1	1.716929134	UP	TMEM191A	-1.701570681	Down
COMMD8	1.715222141	UP	S100A13	-1.701444223	Down
ZNF439	1.715	UP	KITLG	-1.700332628	Down
F3	1.714826624	UP	ZNF142	-1.698729583	Down
IER5	1.714623882	UP	CCDC101	-1.698704104	Down
COPS7B	1.714046075	UP	PIR	-1.698687664	Down
LOC647859	1.713502399	UP	UTP11L	-1.698102557	Down
PXMP3	1.712969525	UP	C21orf58	-1.697616507	Down
NIP30	1.711142654	UP	MGEA5	-1.697579326	Down
PTPRA	1.70997191	UP	ZNF791	-1.697384428	Down
ZNRD1	1.70974412	UP	CXorf57	-1.69718471	Down
USP4	1.709143969	UP	GSTA1	-1.696969697	Down
PPP1R15A	1.708100639	UP	KIAA0460	-1.696938246	Down
ARFGEF1	1.70760015	UP	C16orf91	-1.696529459	Down
TRIML2	1.70729927	UP	FBXO6	-1.696141479	Down
SH3BP1	1.707145989	UP	FAM89A	-1.696091421	Down
ANKRD39	1.706717452	UP	DNAJB4	-1.695899772	Down
REPS2	1.706521739	UP	ERMAP	-1.695100865	Down
ZYX	1.706375484	UP	LOC728431	-1.694925029	Down
CNGB1	1.70625889	UP	FBXO28	-1.693896178	Down
LY6K	1.70624152	UP	TRIP11	-1.693204937	Down
SIX4	1.705709295	UP	GNB1L	-1.692461451	Down
ATRN	1.705670272	UP	LOC85390	-1.692307692	Down
KIAA1688	1.705194452	UP	KRIT1	-1.692167577	Down
LOC100134407	1.704918033	UP	ATP6V0D1	-1.692161158	Down
GRHL2	1.704712612	UP	ATXN7L2	-1.691618682	Down
PWWP2B	1.70460251	UP	C7orf23	-1.691185349	Down
TMEM30A	1.704545455	UP	SLC43A2	-1.690781797	Down
MTA1	1.704521471	UP	SLC4A5	-1.690444145	Down
ANAPC1	1.70431328	UP	TIMELESS	-1.689964563	Down
TAOK1	1.704271123	UP	SOCS1	-1.689798032	Down
DNMT3B	1.704159344	UP	LOC730316	-1.689626685	Down
ZBTB24	1.704098555	UP	MBTD1	-1.6896	Down
FAM70B	1.704009434	UP	SNORA70	-1.689400164	Down
DUSP10	1.703264095	UP	RAP1GDS1	-1.689372851	Down
UBR5	1.703240554	UP	MOSC1	-1.689186073	Down
SLC25A39	1.702505481	UP	CHST7	-1.688605356	Down
NBN	1.70228582	UP	RPL7L1	-1.688245491	Down
MAF1	1.700944669	UP	CEBPA	-1.687827912	Down
LSS	1.700899032	UP	LYRM7	-1.687557043	Down
NAV2	1.700723831	UP	REEP6	-1.687455705	Down
LOC100129034	1.700404858	UP	GALC	-1.687356801	Down
PPP2R3B	1.699882075	UP	PTPLAD2	-1.686615447	Down
C9orf21	1.699827883	UP	TMTC1	-1.684931507	Down
LOC653119	1.699650175	UP	PRKAR1B	-1.684842584	Down
TNFRSF10B	1.699423631	UP	KIAA0664	-1.684585081	Down

TRIP6	1.699168239	UP	MEF2A	-1.684139785	Down
LYPD5	1.699055331	UP	GBA	-1.683366733	Down
PRKRIP1	1.698735739	UP	SF3B3	-1.682673267	Down
PHF5A	1.697557383	UP	SYT7	-1.682457054	Down
TACC1	1.697396387	UP	CIB1	-1.682113597	Down
AEBP2	1.696396627	UP	IMPA2	-1.681534721	Down
FKBP2	1.696335079	UP	LOC100132247	-1.681057517	Down
LOC100130556	1.694968553	UP	KCTD15	-1.68099711	Down
USP12	1.694503622	UP	HNRNPA2B1	-1.680749262	Down
TWF2	1.69310987	UP	WARS	-1.68048929	Down
LOC645630	1.693092622	UP	SNHG8	-1.680234261	Down
ZAK	1.691884457	UP	ATP5S	-1.679843614	Down
PDE6D	1.691232092	UP	S100A16	-1.679395427	Down
SYS1	1.691147379	UP	TPM4	-1.679008152	Down
UBE2F	1.690780283	UP	GINS4	-1.678837052	Down
LOC729372	1.690211346	UP	FLJ12949	-1.677817473	Down
D2HGDH	1.689771198	UP	FAF2	-1.677806341	Down
SFT2D1	1.68963082	UP	RXRA	-1.676765108	Down
LOC728755	1.689239497	UP	TSPYL5	-1.676436782	Down
PRRG1	1.689164538	UP	SBDSP	-1.676098849	Down
CDCA7	1.688916623	UP	SYCE2	-1.675511751	Down
HK1	1.688132165	UP	SEPW1	-1.675126307	Down
TTK	1.686642717	UP	VAV3	-1.674850697	Down
C6orf148	1.686621551	UP	S100A11	-1.674678721	Down
IMPDH1	1.686491935	UP	DST	-1.674607472	Down
KIAA1644	1.686287625	UP	HIST1H3H	-1.674271229	Down
PHACTR3	1.685802948	UP	CCM2	-1.673997535	Down
MDFIC	1.685754851	UP	LOC147727	-1.673560841	Down
CTBP1	1.685191134	UP	MTX3	-1.673200744	Down
RAB11FIP4	1.685013624	UP	CALM1	-1.673143564	Down
RRM2B	1.68496994	UP	SLC44A1	-1.673087426	Down
FAM83D	1.684709964	UP	FTSJ3	-1.672316384	Down
RSU1	1.684557129	UP	NR2C2AP	-1.671940588	Down
C8orf41	1.684402554	UP	NLN	-1.671826625	Down
C17orf39	1.684020825	UP	DHDDS	-1.671528752	Down
RNF4	1.683596554	UP	LOC653610	-1.67031364	Down
C6orf168	1.683571051	UP	FLJ31306	-1.670037254	Down
LOC730167	1.68248308	UP	SNHG9	-1.669252078	Down
RNF10	1.682382134	UP	C10orf116	-1.669215221	Down
GMDS	1.682074091	UP	MAP7D2	-1.669178598	Down
DCLRE1A	1.680876258	UP	C12orf48	-1.668522385	Down
LOC654103	1.680451589	UP	SLC44A4	-1.668375917	Down
MKNK2	1.679979606	UP	PSMB10	-1.667834113	Down
MED14	1.679651321	UP	ZNF786	-1.667560045	Down
BCL3	1.679270111	UP	DHX33	-1.667408851	Down
USP9X	1.679245283	UP	SEZ6L2	-1.667299178	Down
KLHDC9	1.678989526	UP	C22orf29	-1.667136812	Down
CDC34	1.678972713	UP	CBR3	-1.666991474	Down
ICAM2	1.678680982	UP	PLP2	-1.66625	Down
KIAA0363	1.678217822	UP	CCPG1	-1.666195857	Down
PCMT1	1.677965258	UP	PPIL3	-1.666169402	Down

UBE2L3	1.677533279	UP	LOC732425	-1.666069509	Down
PCTP	1.676716235	UP	FGFR3	-1.665947587	Down
ENO2	1.676500509	UP	PEG10	-1.665823852	Down
PAN3	1.67645951	UP	LOC100170939	-1.665229885	Down
HSPH1	1.676037114	UP	EXOSC6	-1.664898939	Down
BTBD11	1.674626866	UP	LTBP4	-1.664889566	Down
HMBOX1	1.67432784	UP	UBIAD1	-1.664788144	Down
SCML2	1.674050633	UP	TRIM5	-1.664383562	Down
CLIC4	1.673855467	UP	EEF1B2	-1.66417121	Down
TMEM61	1.673511294	UP	LOC100133607	-1.664057565	Down
PAXIP1	1.673095656	UP	PRPF3	-1.6635183	Down
KIAA1549	1.671957672	UP	FAM98A	-1.663160355	Down
NIPAL4	1.671883432	UP	QTRT1	-1.662404092	Down
AHCY	1.671405445	UP	PLAA	-1.662254483	Down
KIAA0427	1.670126874	UP	TERF2IP	-1.661317285	Down
ZSCAN12L1	1.669800885	UP	ZDHHC7	-1.661206255	Down
PHKA1	1.668888889	UP	C19orf66	-1.660830861	Down
ARHGAP10	1.668625147	UP	PRPSAP2	-1.660578994	Down
TM9SF3	1.668528723	UP	S100A6	-1.660264768	Down
STX10	1.668483866	UP	HIRIP3	-1.660108765	Down
RRP8	1.668381241	UP	BZW2	-1.66000113	Down
TEAD4	1.668282176	UP	NUDT1	-1.659846154	Down
LOC391825	1.668104879	UP	STRADA	-1.659405941	Down
PCNP	1.667760893	UP	SCARNA16	-1.658572122	Down
RBPMS2	1.667621777	UP	LOC729978	-1.658358715	Down
PIGF	1.667499108	UP	RAD21	-1.658302583	Down
PAICS	1.666757003	UP	RPP40	-1.658279724	Down
POP7	1.666712957	UP	DBNDD2	-1.65764477	Down
LPIN1	1.665264929	UP	SRRD	-1.656685499	Down
AGPAT5	1.664003409	UP	SMA4	-1.656262042	Down
CDCA3	1.663651226	UP	SLC1A4	-1.655534351	Down
LOC441019	1.663424482	UP	FASTKD2	-1.655523719	Down
KIAA0514	1.663327769	UP	LOC653375	-1.655119323	Down
LOC652324	1.663157895	UP	LOC100133298	-1.654859611	Down
PREP	1.662918477	UP	C5orf37	-1.654443255	Down
ADSL	1.662705121	UP	TMEM203	-1.653764793	Down
SGSH	1.662388685	UP	EXOC7	-1.653208363	Down
MTMR6	1.662192394	UP	GCLM	-1.652964752	Down
LOC100134301	1.661997788	UP	SH3GLB1	-1.652649745	Down
FAU	1.660561995	UP	KIAA0430	-1.652372164	Down
GPSM3	1.660350877	UP	BAZ1A	-1.652229462	Down
CD1D	1.659859155	UP	LOC253039	-1.651560178	Down
PLEKHJ1	1.659455128	UP	ATL1	-1.651497006	Down
CALM3	1.658051755	UP	MRPL22	-1.651283619	Down
IFFO1	1.65801105	UP	SULT1A4	-1.65	Down
KHDC1	1.657574641	UP	LOC149448	-1.649734982	Down
MKL1	1.655996178	UP	LOC400721	-1.649505361	Down
XYLT2	1.655981417	UP	NRIP1	-1.649454063	Down
CARS	1.655751831	UP	CLIP4	-1.648653285	Down
PHB	1.655751225	UP	MORF4L2	-1.648136522	Down
GRK5	1.655512322	UP	PTGS1	-1.647675103	Down

VASN	1.655462185	UP	RING1	-1.647130564	Down
IPO5	1.654341164	UP	TTC14	-1.646920668	Down
NMNAT2	1.654320988	UP	SAFB2	-1.646412826	Down
PRKRA	1.653773838	UP	NUP214	-1.646107646	Down
FAM84B	1.653455868	UP	PPP3CC	-1.645993031	Down
HOXC4	1.652941176	UP	ENDOG	-1.64595138	Down
LOC387882	1.652917567	UP	SLC35E1	-1.64591955	Down
LOC651436	1.652546736	UP	MLKL	-1.645317094	Down
EIF2S3	1.652212343	UP	SMCR5	-1.645198062	Down
BRI3P1	1.65195369	UP	PIAS2	-1.644965278	Down
MTDH	1.651819083	UP	SBDS	-1.644794675	Down
LOC729255	1.651790093	UP	AGTRAP	-1.644526445	Down
MAGEE1	1.650068213	UP	RAB31	-1.644310902	Down
ATP6V1H	1.650026205	UP	MKNK1	-1.643710191	Down
PRCP	1.649434389	UP	LOC654096	-1.643564356	Down
SALL4	1.649230769	UP	NID1	-1.643406268	Down
KIAA0146	1.649113522	UP	SERPINB8	-1.643349988	Down
EGLN1	1.649054983	UP	PTN	-1.643114636	Down
SLC25A10	1.648815482	UP	LOC100129539	-1.642602949	Down
ZNF416	1.648498845	UP	DOCK6	-1.642424242	Down
CENTG2	1.647617239	UP	PSPH	-1.642261595	Down
NUTF2	1.647406384	UP	DDX28	-1.641529857	Down
SFRS15	1.646480535	UP	LOC730083	-1.641509434	Down
FNBP1L	1.646115906	UP	LOC100133772	-1.641059067	Down
SOX7	1.645960503	UP	FOXL2	-1.640652301	Down
ZDHHC16	1.645286826	UP	FKTN	-1.640546436	Down
C11orf51	1.645247657	UP	LHPP	-1.639123442	Down
MRFAP1	1.644665612	UP	FBXW9	-1.638941044	Down
PABPC1L	1.644146229	UP	C1orf216	-1.638297872	Down
RARA	1.644129407	UP	SURF1	-1.638211382	Down
TMEM67	1.643976898	UP	ECE2	-1.637663886	Down
C8orf47	1.643855776	UP	EPHA4	-1.637621024	Down
SUMO3	1.643012663	UP	RAB3GAP2	-1.637516689	Down
DHTKD1	1.642149929	UP	CUTC	-1.63679485	Down
B4GALT3	1.641832918	UP	ALDH1A2	-1.636103152	Down
CDC26	1.6415371	UP	SNORA61	-1.636013376	Down
LOC729964	1.641491342	UP	THUMPD1	-1.635342892	Down
ZFP30	1.641025641	UP	LOC100131594	-1.635125448	Down
FEN1	1.640768309	UP	UBN1	-1.634786544	Down
SIGIRR	1.639953542	UP	LOC100130919	-1.634684034	Down
ESRRAP2	1.639897812	UP	FMO4	-1.63427257	Down
AURKA	1.639594314	UP	TRUB2	-1.633807267	Down
EIF4EBP1	1.639415482	UP	GPATCH4	-1.633561124	Down
PAQR3	1.639344262	UP	SMEK2	-1.633309061	Down
PACS2	1.638981636	UP	ZNF524	-1.633304573	Down
GPD1L	1.638954519	UP	C19orf48	-1.633079848	Down
CRYZL1	1.63881504	UP	VTRNA1-2	-1.632746748	Down
ISM1	1.638579387	UP	ARMCX5	-1.632183908	Down
DLGAP4	1.63812601	UP	PION	-1.632055749	Down
UTP6	1.637812047	UP	XPR1	-1.63195377	Down
UCKL1	1.637431739	UP	LILRB3	-1.631564366	Down

SLC9A7	1.637214137	UP	KLHL13	-1.631202503	Down
ORAI1	1.637042569	UP	C1orf93	-1.631104034	Down
PRPSAP1	1.636784512	UP	LOC100133565	-1.630952381	Down
LOC100128775	1.63619648	UP	CCBL1	-1.630780092	Down
MFF	1.63565365	UP	SCAMP5	-1.628712871	Down
KCTD9	1.63482366	UP	SOX15	-1.628463706	Down
LOC441294	1.634803922	UP	ZNF79	-1.627671541	Down
MAPKAPK3	1.634362956	UP	HCG4	-1.627478055	Down
ZNF277	1.634223919	UP	BARD1	-1.626759309	Down
FAM69A	1.634200616	UP	ALDH1A3	-1.626015802	Down
NRBP2	1.6338245	UP	NOL3	-1.625954198	Down
RNF149	1.633526705	UP	LOC729603	-1.625266124	Down
PHYH	1.6332741	UP	CSAD	-1.624865447	Down
TMEM41B	1.633176312	UP	LOC642947	-1.624752294	Down
ZNF773	1.632745878	UP	SLC25A14	-1.624364123	Down
STK40	1.631849756	UP	LOC440341	-1.624	Down
GTSE1	1.631710017	UP	PSMC3IP	-1.623682076	Down
UHRF1BP1	1.631663974	UP	FER	-1.623523622	Down
RNF38	1.631199279	UP	IQCA1	-1.623361823	Down
LRP4	1.631130064	UP	CYB5B	-1.62335762	Down
LOC646786	1.630143541	UP	SEPX1	-1.623029683	Down
HTRA1	1.628833615	UP	EXOSC10	-1.622972614	Down
FLJ25006	1.628535512	UP	MTMR15	-1.622749591	Down
NUFIP2	1.628154488	UP	EIF3K	-1.622671494	Down
NLRP3	1.62767475	UP	IFIT5	-1.622616984	Down
LOC389404	1.627611093	UP	TOE1	-1.621019108	Down
ADIPOR1	1.62741232	UP	SCARNA18	-1.620349279	Down
ZC3HC1	1.627408436	UP	SDHALP1	-1.619842165	Down
AK3L1	1.626039829	UP	ADD3	-1.619779165	Down
ADCY6	1.625584112	UP	NGRN	-1.619626237	Down
GBP4	1.62475182	UP	GMPS	-1.619245524	Down
CTGF	1.624486264	UP	LOC100133019	-1.618693574	Down
NPEPPS	1.624417354	UP	NLRC5	-1.618156618	Down
PRR11	1.624396584	UP	TMBIM4	-1.618035969	Down
ELP3	1.623687344	UP	FAT2	-1.617957396	Down
ACSL1	1.623207301	UP	ZNF750	-1.617586912	Down
TMEM51	1.623191298	UP	ATP7A	-1.617056856	Down
C3orf26	1.623028254	UP	MCC	-1.617021277	Down
ZFAND1	1.622993811	UP	TRIM41	-1.617002237	Down
MCM4	1.622630866	UP	ZNF30	-1.616649538	Down
LOC441550	1.622063758	UP	ATPIF1	-1.616606688	Down
TMEM145	1.622047244	UP	LOC643911	-1.616212339	Down
AKAP7	1.62204192	UP	CMTM3	-1.615797172	Down
TIGD2	1.62197873	UP	ANKLE2	-1.615384615	Down
CUTL1	1.621665175	UP	CLEC11A	-1.615017065	Down
TOP1P2	1.621609799	UP	TRIM21	-1.61488875	Down
PRPF19	1.621099185	UP	LOC100133697	-1.61465721	Down
CSRP2BP	1.620714543	UP	C6orf170	-1.614413681	Down
MAP6D1	1.620559335	UP	LOC728178	-1.613533835	Down
RNF19A	1.620197212	UP	PSMB7	-1.613301937	Down
LSM2	1.620020655	UP	PPARGC1B	-1.613176778	Down

NAT10	1.619733131	UP	PLA2G2D	-1.613031915	Down
LOC651575	1.618720481	UP	KIAA1407	-1.612817089	Down
SAR1A	1.618299882	UP	NMRAL1	-1.612789402	Down
SAP30L	1.617657429	UP	LRP5L	-1.612743363	Down
B3GALNT1	1.617647059	UP	HAT1	-1.612387517	Down
DDX50	1.617632191	UP	LOC645233	-1.611052072	Down
PIP4K2A	1.617302053	UP	CLCN7	-1.61079299	Down
CD59	1.617102258	UP	LRIG3	-1.610446137	Down
C21orf7	1.616760347	UP	LOC652826	-1.61043095	Down
SAC3D1	1.615577889	UP	LOC644511	-1.61036075	Down
FAM178B	1.615	UP	MGA	-1.609960159	Down
LOC649095	1.614777618	UP	MGC72104	-1.6095791	Down
MCM7	1.61466757	UP	LOC643336	-1.60940775	Down
RCOR2	1.6145686	UP	LOC25845	-1.609310261	Down
HOXB2	1.614448669	UP	SLC6A2	-1.609230769	Down
TRPC4AP	1.613055746	UP	ELF1	-1.609171278	Down
TEX261	1.612180499	UP	C12orf51	-1.609137056	Down
C6orf52	1.612113402	UP	NOL8	-1.608237313	Down
EXTL3	1.612060467	UP	AKR1D1	-1.608208739	Down
SPIN4	1.611613876	UP	HDAC1	-1.60807399	Down
BOP1	1.611485975	UP	BMP2	-1.607342572	Down
MRS2	1.611481976	UP	WASF3	-1.607304217	Down
SLC37A3	1.611244429	UP	LIN52	-1.606754117	Down
MOSPD3	1.611111111	UP	YDJC	-1.606519681	Down
TMBIM6	1.611056323	UP	TMEM126A	-1.606339163	Down
SNX17	1.61076719	UP	SEMA5A	-1.606324111	Down
LOC730744	1.610494378	UP	BMPR2	-1.606185567	Down
C12orf24	1.61026647	UP	SPTLC1	-1.604805399	Down
PHF12	1.608556577	UP	EIF3J	-1.604549951	Down
ZNF418	1.608365019	UP	LOC100132439	-1.603872318	Down
C17orf75	1.608297743	UP	FKBP10	-1.603305785	Down
C9orf46	1.607670164	UP	NTN1	-1.602143758	Down
FLJ45248	1.607619048	UP	ZBED5	-1.602123321	Down
VPS37D	1.607489598	UP	C5orf15	-1.601459416	Down
FTL	1.607344711	UP	ZCCHC14	-1.601381876	Down
PUS7	1.606954606	UP	NOP16	-1.601241135	Down
MED30	1.606043473	UP	ORC2L	-1.600907821	Down
C17orf61	1.605843897	UP	DUOX1	-1.600630347	Down
SYVN1	1.605386594	UP	BLVRB	-1.600317069	Down
RTN4IP1	1.605333986	UP	LOC100134868	-1.600098945	Down
ANKRD2	1.605323194	UP	NOLC1	-1.599803236	Down
SUB1	1.605122951	UP	IER5L	-1.599635036	Down
HEATR6	1.604516263	UP	LOC652773	-1.5994	Down
SMAGP	1.604220779	UP	B2M	-1.599209234	Down
NCOA4	1.603724524	UP	APOBEC3B	-1.597800613	Down
STC2	1.60363181	UP	SNORD35B	-1.59762309	Down
C11orf74	1.603042728	UP	FEM1C	-1.597484277	Down
FBXL20	1.602937102	UP	MYC	-1.597333533	Down
TUT1	1.602711157	UP	LOC153684	-1.59729064	Down
NTN4	1.602512156	UP	DYNC1H1	-1.597166234	Down
PPP1CC	1.602464844	UP	CSTF2T	-1.596949891	Down

GRINA	1.602244571	UP	C11orf85	-1.5969163	Down
GMEB2	1.60173913	UP	YEATS2	-1.595440044	Down
PRSS22	1.601461495	UP	CD97	-1.594629156	Down
CD151	1.601433964	UP	FBXO46	-1.59455819	Down
TRAPPC2	1.601421189	UP	C19orf62	-1.594265415	Down
GNG10	1.601324556	UP	MIS12	-1.593585392	Down
GSTZ1	1.60100091	UP	PITX2	-1.593501805	Down
MIA	1.600798403	UP	TMC6	-1.593473827	Down
SCGB1A1	1.600593912	UP	GYLTL1B	-1.593468468	Down
FAM60A	1.600555507	UP	OXSM	-1.592746978	Down
C8orf59	1.600465375	UP	FZD8	-1.592643997	Down
TBC1D19	1.600229533	UP	KIAA0907	-1.592568682	Down
LOC255783	1.600160149	UP	TUBB4Q	-1.591947241	Down
BUB1	1.599692202	UP	UBA7	-1.591854673	Down
CXADR	1.599561029	UP	LOC100132805	-1.591463415	Down
C7orf44	1.599549977	UP	CWF19L2	-1.591124127	Down
NUFIP1	1.599531616	UP	LOC644590	-1.590400746	Down
CTDSP2	1.599337533	UP	RPUSD1	-1.59	Down
SEC62	1.598837209	UP	LOC285908	-1.589716122	Down
PKD2	1.598622417	UP	PSMD12	-1.589195584	Down
DSE	1.598430524	UP	BAX	-1.588556851	Down
NUDT22	1.598397526	UP	ITGA6	-1.588522589	Down
ATOX1	1.598355375	UP	ZNF311	-1.588474576	Down
UGT8	1.598320504	UP	ADAR	-1.588054566	Down
MEGF6	1.597683975	UP	BOLA2	-1.587325694	Down
LOC728825	1.597553699	UP	GORASP2	-1.587302833	Down
ATP6V1E2	1.596819457	UP	ITGAV	-1.587226457	Down
CCT4	1.596213292	UP	LRIG2	-1.587096774	Down
TIMM9	1.596197818	UP	LOC100133477	-1.58692914	Down
PRDX6	1.596103116	UP	NUP62	-1.58611001	Down
CSNK2B	1.595919184	UP	TIA1	-1.585623258	Down
LEMD2	1.595775941	UP	DKFZp434K191	-1.585185185	Down
BRAF	1.595730028	UP	C2orf89	-1.584580838	Down
RNF138	1.595551521	UP	SNORA73A	-1.583809524	Down
HEG1	1.595281656	UP	NCRNA00153	-1.583653846	Down
ZNF217	1.595230722	UP	WIPI1	-1.583431953	Down
ADORA2B	1.594749735	UP	SARS2	-1.583370746	Down
ITGA2	1.594592513	UP	UBE4B	-1.582494476	Down
TFG	1.594172932	UP	KIAA0194	-1.582440504	Down
PFKP	1.593796515	UP	PYCR1	-1.581574884	Down
ZFAND3	1.593480704	UP	RSBN1L	-1.580994077	Down
ANKRD13A	1.593226998	UP	ABHD6	-1.580809434	Down
SLC41A3	1.592584655	UP	PARP14	-1.58013289	Down
NIPA1	1.592122704	UP	MOCOS	-1.580117278	Down
FTHL11	1.592095614	UP	KARS	-1.580067931	Down
EXOC4	1.591836735	UP	ADAL	-1.580023364	Down
KIAA1279	1.59178744	UP	COX10	-1.580020812	Down
FAM58A	1.591714105	UP	LOC729252	-1.579751131	Down
DYNLL2	1.590257171	UP	ZC3H4	-1.579738679	Down
KIAA1530	1.590174531	UP	DBR1	-1.579423265	Down
MST4	1.589635114	UP	ELAC2	-1.578958646	Down

LASP1	1.589412374	UP	ABCG1	-1.578866769	Down
API5	1.589269708	UP	C11orf1	-1.578558875	Down
YIF1A	1.588521263	UP	SNAPC3	-1.577715356	Down
SUCLG2	1.58827265	UP	KLF5	-1.577708006	Down
CCDC53	1.587838462	UP	KIF21A	-1.577617685	Down
CDCA1	1.587753865	UP	TFB1M	-1.577133389	Down
COQ2	1.587386356	UP	EVI1	-1.576767092	Down
ZNF215	1.587053571	UP	CHCHD9	-1.576411873	Down
LOC100129960	1.586968228	UP	RFWD3	-1.576164769	Down
TSC22D2	1.586938776	UP	ITGB8	-1.57578125	Down
STAT4	1.586797066	UP	BAG2	-1.575637643	Down
CCS	1.586246638	UP	KLHL12	-1.575061816	Down
DPH3	1.585903084	UP	THADA	-1.574759945	Down
SMAD3	1.585290579	UP	AMDHD2	-1.5746859	Down
TPD52	1.585	UP	PLA2G4C	-1.57442866	Down
LOC100132648	1.584770115	UP	ISG20L1	-1.57442165	Down
AHCYL2	1.584738243	UP	VPS18	-1.574087079	Down
FEZ1	1.584613237	UP	MAP3K5	-1.573883162	Down
SAA4	1.583994232	UP	RHBDD1	-1.573770492	Down
CTNNBL1	1.583864318	UP	POF1B	-1.573391179	Down
CCDC102A	1.58384809	UP	TRABD	-1.573306509	Down
ANO10	1.583669355	UP	CENPBD1	-1.573116691	Down
TRAM2	1.583576998	UP	SPG7	-1.572651357	Down
WAC	1.583521809	UP	FAM153B	-1.572352064	Down
GNAQ	1.583500502	UP	PRKX	-1.572318647	Down
MRPL23	1.582697608	UP	KIAA0090	-1.571655856	Down
DHX16	1.582402881	UP	LIPG	-1.570957096	Down
GNL1	1.58237356	UP	UQCRB	-1.570759813	Down
LOC100134794	1.582119205	UP	SRXN1	-1.570667081	Down
MOXD1	1.581967213	UP	NIF3L1	-1.57026687	Down
TIMP4	1.581805838	UP	PIK3R2	-1.570005101	Down
CSDA	1.581625809	UP	SPCS3	-1.569859088	Down
LOC723972	1.580997218	UP	C12orf5	-1.569815989	Down
GGCX	1.580823814	UP	LOC554203	-1.569751844	Down
WSB1	1.580760856	UP	TMEM106B	-1.569552038	Down
NUCKS1	1.580633016	UP	XPNPEP1	-1.569267159	Down
ZNF618	1.58059142	UP	LOC642946	-1.569239501	Down
DONSON	1.580564625	UP	CSNK1G1	-1.569217541	Down
FJX1	1.580494077	UP	LOC613037	-1.56909327	Down
GLCE	1.580160955	UP	HAS2AS	-1.569032979	Down
EVL	1.580057739	UP	GAS1	-1.568986948	Down
LOC340274	1.579087049	UP	PPM1K	-1.568878974	Down
PAPSS2	1.578918624	UP	CDC42EP4	-1.568692343	Down
PIK3C2A	1.57868937	UP	ZBTB43	-1.568303231	Down
PDE12	1.578264969	UP	EDN1	-1.56793049	Down
LOC729645	1.577808793	UP	CCDC132	-1.566132265	Down
WDR22	1.577770974	UP	TMEM37	-1.565980168	Down
SP140	1.576519916	UP	SLK	-1.565963061	Down
INTS2	1.576286353	UP	PLAUR	-1.565773594	Down
ERGIC3	1.576277393	UP	TMEM55B	-1.56554473	Down
RBBP4	1.575622543	UP	MIRLET7D	-1.565365025	Down

PTK2	1.57471308	UP	HNRNPH1	-1.56533141	Down
APLP2	1.574087863	UP	FBXW10	-1.565	Down
ARMC1	1.574039404	UP	KIDINS220	-1.564976515	Down
NBPF10	1.573975045	UP	TTC23	-1.564906303	Down
ERLIN2	1.573972244	UP	LOC653450	-1.564193168	Down
RPN1	1.573620243	UP	MAP3K1	-1.563976378	Down
HIP2	1.573430294	UP	LOC728324	-1.563915388	Down
MYEOV	1.572893773	UP	TTF2	-1.563727034	Down
ST3GAL1	1.572785884	UP	RARB	-1.563662375	Down
PGBD1	1.572405471	UP	CAMSAP1L1	-1.563606824	Down
UBE2C	1.572326891	UP	C19orf54	-1.563478261	Down
AS3MT	1.57209033	UP	PRDM4	-1.563203565	Down
COL13A1	1.571774682	UP	MRPS15	-1.563092212	Down
CKAP2	1.571531941	UP	TIMM44	-1.562840114	Down
PDZD2	1.571428571	UP	SMTN	-1.5625966	Down
LOC643300	1.571236559	UP	TPMT	-1.562366611	Down
FANCG	1.570459855	UP	NUCB1	-1.562240334	Down
RBBP7	1.569936108	UP	LANCL2	-1.562200957	Down
F11R	1.5694315	UP	MTMR10	-1.562035891	Down
LGTN	1.569379174	UP	DNAJC30	-1.561704483	Down
CHORDC1	1.56918189	UP	LRRC8B	-1.560906516	Down
RALB	1.569164996	UP	TOMM40L	-1.560867966	Down
ACSL5	1.568181818	UP	SIAH2	-1.560771968	Down
ABCB9	1.56778404	UP	DVL3	-1.560036307	Down
LETM2	1.567215364	UP	DNAJA3	-1.559754783	Down
DIDO1	1.566948731	UP	LOC127295	-1.559709258	Down
RALY	1.566913814	UP	LOC649903	-1.559392265	Down
THOC7	1.565724892	UP	LBA1	-1.559386973	Down
GPR176	1.565697091	UP	IKBIP	-1.559135709	Down
LOC731950	1.565561302	UP	PYCR2	-1.558872214	Down
FUS	1.565431459	UP	VEZT	-1.558421326	Down
POLR2J3	1.565393925	UP	C16orf53	-1.558342314	Down
NPEPL1	1.564898082	UP	LOC728635	-1.55815437	Down
CEBPB	1.564727026	UP	SNRNP25	-1.557991232	Down
CDS2	1.563769396	UP	KIAA1826	-1.557603687	Down
MEN1	1.563243243	UP	UBXN7	-1.557504873	Down
KIF3C	1.563174603	UP	CBWD5	-1.557329608	Down
LOC92249	1.562111801	UP	AFG3L1	-1.557135046	Down
PTPRF	1.561621815	UP	HNRPH1	-1.556836903	Down
TPX2	1.561426155	UP	DDRGK1	-1.555862069	Down
MGC57346	1.561299053	UP	PFKM	-1.555299539	Down
SELK	1.56090052	UP	CCDC58	-1.554933084	Down
SETD3	1.560604959	UP	POP5	-1.554925436	Down
MYST3	1.559608648	UP	ZSCAN5A	-1.554575987	Down
PRPF6	1.559591373	UP	AKR1A1	-1.55401398	Down
AFAP1L1	1.559129902	UP	ZNF692	-1.55346651	Down
LOC644617	1.559116501	UP	IPO4	-1.552888889	Down
CTAGE6	1.558804832	UP	PIK3AP1	-1.552504039	Down
LOC730102	1.558635394	UP	LOC642236	-1.552496626	Down
TSGA14	1.558592761	UP	NAPRT1	-1.552405762	Down
ARHGAP29	1.558431953	UP	EPRS	-1.55225248	Down

IL27RA	1.558222222	UP	LOC645135	-1.552238806	Down
RBM35A	1.557793616	UP	ATP5F1	-1.551963272	Down
LOC100132485	1.557012543	UP	TMEM186	-1.551602986	Down
ZNF597	1.55697446	UP	GPR87	-1.550917777	Down
TTC38	1.556962025	UP	NGB	-1.550879878	Down
DDIT4	1.556891322	UP	ALKBH7	-1.55049303	Down
LOC643319	1.556860517	UP	RNMTL1	-1.550479512	Down
COL18A1	1.556844548	UP	LOC147645	-1.550465839	Down
EXOSC8	1.556788056	UP	GPR114	-1.55026455	Down
WDSOF1	1.556630711	UP	FUBP1	-1.549470899	Down
FKBP1B	1.555835962	UP	PSMB5	-1.548949314	Down
ANAPC11	1.555583313	UP	INPP5F	-1.548837209	Down
G6PC3	1.55548926	UP	C21orf63	-1.548696115	Down
ZNF121	1.555423123	UP	FRAP1	-1.548669128	Down
ACOX2	1.555374593	UP	LOC441763	-1.548405467	Down
ARSI	1.555316401	UP	CRKL	-1.547963899	Down
SNX10	1.555288462	UP	HTR7	-1.547945205	Down
C8orf83	1.555158553	UP	MTHFR	-1.547919294	Down
LOC647037	1.554650799	UP	CRAMP1L	-1.54790304	Down
PDXK	1.553948743	UP	CENTA1	-1.547894206	Down
ZNF655	1.553181607	UP	FARSA	-1.547824491	Down
C6orf156	1.553042122	UP	SELS	-1.547553093	Down
LOC728454	1.552785924	UP	AMY1A	-1.547065868	Down
VLDLR	1.552785924	UP	OGDHL	-1.546885695	Down
LOC100132289	1.551412429	UP	TRIM13	-1.546436916	Down
HTRA2	1.551284967	UP	GTF3C1	-1.546365331	Down
ANKRD52	1.551153679	UP	LOC648189	-1.546218487	Down
NAP1L4	1.55094702	UP	C15orf28	-1.545971074	Down
SIRPA	1.550685895	UP	ADH7	-1.545582048	Down
MESP1	1.550570342	UP	TNPO2	-1.545575302	Down
FAM20B	1.550454978	UP	BCR	-1.544839255	Down
NCCRP1	1.55039884	UP	MTE	-1.544592593	Down
TRPV6	1.549577248	UP	C1orf71	-1.54389117	Down
LRRC34	1.549536707	UP	CYB561D1	-1.543501805	Down
NCOA3	1.548643908	UP	FAM179B	-1.543278689	Down
PDHB	1.548520522	UP	RRAGB	-1.543257612	Down
HSPC157	1.548215642	UP	CCDC18	-1.542517007	Down
DDX17	1.548090198	UP	DECR2	-1.542238267	Down
RAB9A	1.548060087	UP	BAG1	-1.542016807	Down
VWA1	1.547962382	UP	UGT1A1	-1.541547278	Down
TMEM127	1.547951583	UP	CXorf45	-1.5405	Down
PPP2R5E	1.547853373	UP	ATP2A1	-1.540111199	Down
PGM3	1.547804981	UP	CTPS	-1.53977824	Down
NFS1	1.5472167	UP	FBXO2	-1.53945292	Down
LOC644390	1.546899841	UP	MGST2	-1.539403957	Down
LIME1	1.546841674	UP	MYO10	-1.539015877	Down
VRK1	1.546819035	UP	FAM100A	-1.539011704	Down
TCEA2	1.54651781	UP	TBC1D2B	-1.538947974	Down
SGPP1	1.54610951	UP	OS9	-1.538374718	Down
SEC23A	1.545772187	UP	YOD1	-1.53798948	Down
ZBTB42	1.545682889	UP	MTMR3	-1.537802717	Down

GDI2	1.545425961	UP	GEMIN6	-1.537611676	Down
NUDT6	1.544600939	UP	THBS1	-1.536966891	Down
USPL1	1.544476327	UP	LOC441131	-1.536422737	Down
HMMR	1.544338175	UP	PIGM	-1.536385421	Down
KLHDC2	1.543928768	UP	MKI67IP	-1.535963798	Down
C18orf19	1.54374412	UP	F2RL1	-1.535404624	Down
TMEM9B	1.543250572	UP	TNFRSF8	-1.534932957	Down
WASPIP	1.543019481	UP	ECHDC1	-1.534893439	Down
LOC440359	1.542956152	UP	LOC440926	-1.534867213	Down
GPT2	1.542838457	UP	LOC648370	-1.534586971	Down
C20orf54	1.542721519	UP	P15RS	-1.534523923	Down
SPATA20	1.542640187	UP	MGC26356	-1.534227141	Down
ZNF281	1.541780273	UP	CYP1A1	-1.534108135	Down
LOC387867	1.541487627	UP	SYF2	-1.533718026	Down
MTMR4	1.541326938	UP	NCRNA00094	-1.533699634	Down
FTHL8	1.541270177	UP	FLJ12078	-1.533691115	Down
LOC285296	1.541087963	UP	CHD1	-1.533027251	Down
C8orf58	1.540942928	UP	EXOSC2	-1.532901501	Down
PGRMC1	1.540894917	UP	BMP1	-1.532569192	Down
RELL1	1.540244524	UP	CDAN1	-1.532038127	Down
EPS15	1.539843524	UP	DECR1	-1.531780153	Down
CARS2	1.539346812	UP	SETX	-1.531735314	Down
FBXW11	1.539004815	UP	TMEM5	-1.531713997	Down
LSM14A	1.538921564	UP	ETFDH	-1.531125422	Down
NMI	1.538538941	UP	MYB	-1.530232558	Down
CXorf12	1.537817638	UP	ZFAND5	-1.5301293	Down
ZNF609	1.537251124	UP	CUGBP1	-1.529844859	Down
FZD6	1.537134026	UP	ARSJ	-1.529545455	Down
LSM8	1.536796537	UP	MAPKBP1	-1.529317697	Down
RIMS3	1.536653609	UP	SORD	-1.529163145	Down
KLHL3	1.536473291	UP	MTHFSD	-1.529141104	Down
HSPA1L	1.536075949	UP	PLEKHH3	-1.529091898	Down
LOC729739	1.535714286	UP	SLC7A6	-1.528913109	Down
RBX1	1.535691117	UP	ISOC2	-1.528152719	Down
AP2B1	1.535568275	UP	LMNA	-1.528015675	Down
COX17	1.535220188	UP	LOC440349	-1.527807487	Down
TRA1P2	1.534969649	UP	FAM38A	-1.527779992	Down
UTP14C	1.534844384	UP	CLNS1A	-1.527348786	Down
MID2	1.534778226	UP	LSR	-1.527213353	Down
EPHB6	1.534638554	UP	APITD1	-1.526830135	Down
CCDC85B	1.534477567	UP	LRRC56	-1.526404494	Down
PHEX	1.53431085	UP	PCMTD2	-1.526355234	Down
PRKRIR	1.534053045	UP	C9orf78	-1.526305443	Down
IFRD1	1.533726261	UP	MFAP3	-1.526157547	Down
CCNG1	1.533599302	UP	FAM105B	-1.525874392	Down
POLR2B	1.532949202	UP	BRD2	-1.524986727	Down
HSPD1	1.532664327	UP	UBR4	-1.524627721	Down
CABYR	1.532586558	UP	CDRT1	-1.524407252	Down
CCNY	1.532529659	UP	C9orf45	-1.524267179	Down
PON3	1.532266961	UP	GOLGA6B	-1.524221453	Down
PGBD3	1.531857207	UP	IL11	-1.524159021	Down

ABHD10	1.531765973	UP	NDUFA8	-1.524113456	Down
REXO2	1.531698439	UP	RTF1	-1.523322684	Down
SNHG1	1.531465848	UP	SLFN11	-1.523315618	Down
DENND3	1.531219512	UP	LOC100009676	-1.523	Down
TTC9C	1.531186441	UP	DYRK3	-1.522915516	Down
MYBL1	1.531079065	UP	LOC729559	-1.522475795	Down
STK33	1.530918176	UP	ATP6V1E1	-1.521657177	Down
TMOD2	1.530612245	UP	FLJ10803	-1.521587744	Down
SNORD25	1.530459231	UP	CEACAM1	-1.521555368	Down
GTF2E2	1.53040416	UP	USE1	-1.52150245	Down
PMM1	1.530154676	UP	SIGMAR1	-1.52148273	Down
LAPTM4B	1.530116374	UP	GNRHR	-1.521276596	Down
RRP7A	1.530087606	UP	LOC645600	-1.521174478	Down
TMEM189-UBE2V1	1.529678884	UP	WDR59	-1.521069608	Down
PRIM2	1.529481132	UP	DDX39	-1.521060723	Down
LOC339804	1.529295217	UP	APEX1	-1.520598108	Down
C7orf68	1.529256595	UP	C10orf118	-1.520412371	Down
SLC22A5	1.529024878	UP	ZNF519	-1.519924099	Down
INVS	1.528709233	UP	KIAA0408	-1.519736842	Down
FBXO18	1.528660279	UP	TAX1BP1	-1.519384971	Down
LOC100133058	1.52853792	UP	BDKRB1	-1.519187359	Down
CHCHD3	1.528143539	UP	RHOT2	-1.518723994	Down
ZNF772	1.528106509	UP	NACC2	-1.518663194	Down
HSZFP36	1.528024911	UP	MAP7D1	-1.51785576	Down
SGCE	1.527971301	UP	KIAA0913	-1.517235221	Down
MGAT4A	1.527208481	UP	UFC1	-1.517107943	Down
ARL6IP4	1.526944758	UP	IL12RB2	-1.517041801	Down
DBNDD1	1.526940517	UP	CLIP2	-1.517034068	Down
FKBP11	1.526765843	UP	TCF4	-1.516923077	Down
ARHGEF10	1.526603467	UP	LOC730173	-1.516828294	Down
EIF1B	1.526497765	UP	LOC100131810	-1.516376663	Down
CARD10	1.526465028	UP	C19orf25	-1.516313933	Down
WRB	1.526280474	UP	LOC100129441	-1.515938607	Down
MAN2B2	1.526217228	UP	RUNDC2C	-1.515410147	Down
XRN2	1.525976861	UP	UQCRC2	-1.515097908	Down
PRKCDBP	1.525976327	UP	SLC12A6	-1.515054452	Down
QSOX1	1.525731127	UP	KIAA1598	-1.515036183	Down
CKS1B	1.525488961	UP	NOP58	-1.514837819	Down
FAM160B2	1.524986271	UP	RABGGTB	-1.514542866	Down
LOC100134261	1.524946921	UP	TTC32	-1.513910597	Down
C13orf23	1.524464389	UP	GMIP	-1.513705584	Down
PSG7	1.524137931	UP	RFC2	-1.513647643	Down
LRP11	1.522361766	UP	CD24	-1.513116371	Down
LOC100132139	1.522037218	UP	USP10	-1.512784454	Down
LOC654174	1.521480502	UP	C19orf40	-1.512639029	Down
AGFG2	1.521468575	UP	PTK7	-1.512117852	Down
LOC729339	1.52119883	UP	C5orf24	-1.511697362	Down
MPZL1	1.52111885	UP	SCNM1	-1.511427803	Down
C7orf43	1.521072797	UP	SPRYD4	-1.511411411	Down
ATP5J	1.520894374	UP	SURF2	-1.511263841	Down
POLR2K	1.520498184	UP	MYO3B	-1.509915014	Down

LOC652968	1.520487265	UP
EIF6	1.520078257	UP
AK3	1.519969685	UP
LOC728473	1.519374575	UP
PIGG	1.519088163	UP
KIAA1600	1.519059066	UP
NFU1	1.518827579	UP
MCM3AP	1.518825437	UP
MBTPS2	1.518703242	UP
RAC3	1.518634759	UP
AASDH	1.518557059	UP
PFDN4	1.518384257	UP
GPR172A	1.51834119	UP
LIN7B	1.517884131	UP
ZNF354A	1.517810026	UP
TCF7L1	1.517445299	UP
ARHGEF17	1.517391304	UP
UNG	1.515220633	UP
MIR1282	1.515217391	UP
C13orf34	1.515130079	UP
PTDSS1	1.514921496	UP
LOC441505	1.514705882	UP
BTG3	1.513935542	UP
LCAT	1.513856813	UP
CCDC74A	1.513790386	UP
C14orf145	1.512840169	UP
PKP2	1.512762423	UP
FTHL12	1.512583723	UP
PDIA6	1.510966827	UP
LOC651816	1.510962755	UP
BVES	1.510897436	UP
TOMM34	1.510524361	UP
LOC641788	1.510428101	UP
TOMM22	1.509963698	UP
BRMS1	1.509703813	UP
OSBP	1.509552463	UP
PIM1	1.509478673	UP
SLC37A4	1.509453703	UP
LOC388588	1.509280742	UP
NDRG1	1.509188924	UP
C20orf45	1.509103202	UP
TMEM54	1.508794567	UP
RDBP	1.508379102	UP
CHKA	1.50828434	UP
C13orf1	1.507977355	UP
LOC100132106	1.507387083	UP
C6orf72	1.507236842	UP
UBE2V2	1.507076995	UP
LRRC69	1.507002801	UP
BMS1	1.506786779	UP
COMMD10	1.506585469	UP

LRRK1	-1.509745127	Down
NSUN5	-1.509659614	Down
SYNE2	-1.50927398	Down
BCS1L	-1.509148141	Down
HMGCR	-1.509070437	Down
SNORD34	-1.509066488	Down
SUPT5H	-1.509010121	Down
C5orf30	-1.508833922	Down
LOC389328	-1.508588499	Down
RAB8A	-1.508387549	Down
BTF3L4	-1.507696086	Down
SDHAF1	-1.507186291	Down
CCDC49	-1.507038775	Down
LOC729841	-1.506819052	Down
CRYGS	-1.506493506	Down
PSME2	-1.506007523	Down
ZNF532	-1.504188644	Down
HPGD	-1.503937008	Down
MTR	-1.503834534	Down
CREG2	-1.503324468	Down
NRD1	-1.502772969	Down
URB2	-1.502242152	Down
TMEM69	-1.502229071	Down
KIF24	-1.502053388	Down
NAB2	-1.501889385	Down
OGFOD1	-1.501614942	Down
LOC54103	-1.501449275	Down
ZNF24	-1.501242751	Down
TRA2A	-1.500906032	Down
HIST2H4B	-1.500533049	Down
ZC3H18	-1.500434028	Down
GNPTG	-1.5	Down

LOC647285	1.505777454	UP
LOC727901	1.50566343	UP
LRWD1	1.505612829	UP
HIATL1	1.505439815	UP
HTT	1.50523865	UP
EIF2B2	1.505236343	UP
LOC643224	1.505166475	UP
MELK	1.504997441	UP
MGC3196	1.504975124	UP
CALCOCO2	1.504945831	UP
DDHD2	1.504909038	UP
FTHL2	1.504620684	UP
LOC100129543	1.504441624	UP
IGF2BP3	1.504392971	UP
DEDD2	1.503987499	UP
XRCC6	1.503737709	UP
ITPK1	1.50371471	UP
LOC651064	1.502831492	UP
GIYD1	1.502786624	UP
AGPAT9	1.50239521	UP
RBPJ	1.501798561	UP
IRAK1BP1	1.501211827	UP
RNU1F1	1.501138952	UP
RAGE	1.500718342	UP
PITRM1	1.500609137	UP
LOC345041	1.500236496	UP
DEK	1.50004658	UP
