# Supplementary material for: Gene dysregulation by histone variant H2A.Z in bladder cancer
Source: Epigenetics Chromatin. 2013 Oct 16;6:34. doi: 10.1186/1756-8935-6-34 (PMC3853418; doi:10.1186/1756-8935-6-34)
Supplement: Additional file 4: Table S3 — Genes overlapping with ChIP-seq and gene expression array. [file 1756-8935-6-34-S4.pdf]

Supplementary Table S3. Genes overlapping with ChIP-seq and gene expression array.

SYMBOL	Fold change (LD611/UROtsa)	up/down
KCNS1	2.525801953	UP
SSRP1	2.52126932	UP
KIAA1429	2.574066123	UP
CCND1	2.558970436	UP
KPNA3	2.557268457	UP
ADAP2	2.480300188	UP
MMP7	2.420414462	UP
C4orf14	2.402333459	UP
CHMP2B	2.347372045	UP
C13orf37	2.335968044	UP
COPS6	2.334668609	UP
TMEM97	2.319624901	UP
BIRC3	13.93728981	UP
BIRC2	12.49544304	UP
TIMM10	10.93895349	UP
CST6	10.35436537	UP
ORAOV1	10.10729927	UP
YAP1	9.693527756	UP
TMEM123	5.731285704	UP
ELOVL4	5.378778135	UP
NT5E	5.361380211	UP
ZNF22	5.254996157	UP
RTTN	4.682654112	UP
PPFIA1	4.528189911	UP
STX3	4.138461538	UP
MGC87042	4.085068935	UP
STK3	3.795478036	UP
FADD	3.792809177	UP
DDX43	3.466197183	UP
KIAA0100	3.447619048	UP
C17orf79	3.366404098	UP
ZNF415	3.091636364	UP
MED19	3.006079027	UP
GPR137	2.88683183	UP
DHCR7	2.777877095	UP
BSCL2	2.770724992	UP
SLC46A3	2.741606715	UP
UNC119	2.668918919	UP
MRPL11	2.668682315	UP
DHRS13	2.625879805	UP
MRPL49	2.241886919	UP
SSPN	2.234375	UP
SSSCA1	2.233678902	UP
SH3BGRL2	2.230430528	UP
ZNF433	2.2	UP
IFT20	2.193853974	UP
PNPLA2	2.193243734	UP

SYMBOL	Fold change (LD611/UROtsa)	up/down
KIF24	-1.502053388	Down
TRIM13	-1.546436916	Down
TOMM40L	-1.560867966	Down
TFB1M	-1.577133389	Down
PARP14	-1.58013289	Down
TRIM21	-1.61488875	Down
TRIM5	-1.664383562	Down
GINS4	-1.678837052	Down
ATP5S	-1.679843614	Down
ZRANB2	-1.701997634	Down
TMTC3	-1.706507304	Down
RGS2	-1.731543624	Down
PCDHB9	-1.813781073	Down
PPDPF	-1.880868956	Down
C20orf177	-1.895605293	Down
WDR19	-1.965562337	Down
CYTH1	-2.158547587	Down
ZNF682	-2.859976356	Down
IFI35	-3.580645161	Down
CHURC1	-3.952495262	Down
HLA-F	-5.048695488	Down
HCP5	-7.391121192	Down
PTGS2	-13.53127753	Down

C7orf59	2.188658744	UP
PCBD1	2.177754312	UP
RAB1B	2.165012407	UP
CBLL1	2.14849921	UP
CPNE3	2.142635715	UP
RPS19BP1	2.123197903	UP
HSF2	2.121158242	UP
MTCH2	2.11223822	UP
PCDHB5	2.0982009	UP
SF3B2	2.057241353	UP
ATF2	2.041741205	UP
MED28	2.008926358	UP
PIGU	1.97407274	UP
ZDHHC24	1.958916084	UP
ITFG2	1.944837758	UP
ZNF615	1.938271605	UP
CDCA5	1.915212618	UP
LYN	1.908219301	UP
C21orf56	1.901515152	UP
POLDIP2	1.898645258	UP
TMEM199	1.895711144	UP
OTUB1	1.885819521	UP
CLP1	1.882711672	UP
ANTXR1	1.877530864	UP
VPS37A	1.873841768	UP
CREB3L2	1.871705544	UP
RCE1	1.858243451	UP
FER1L4	1.853708791	UP
PMPCB	1.853285112	UP
SLC35B4	1.848	UP
SLC35F5	1.847119224	UP
DDB1	1.838951311	UP
PC	1.829751131	UP
PPP2R5C	1.827215756	UP
IMPAD1	1.822707081	UP
ILK	1.810519167	UP
WFS1	1.791302448	UP
RBM12B	1.787804878	UP
LPCAT3	1.780753518	UP
LY6E	1.780517401	UP
FNTB	1.780104712	UP
GANAB	1.777744041	UP
WWP1	1.754518887	UP
SLC25A15	1.736013614	UP
WDR74	1.73253406	UP
CFL1	1.724173816	UP
TAF4	1.719794344	UP
ZNRD1	1.70974412	UP
GRHL2	1.704712612	UP
NBN	1.70228582	UP
RRM2B	1.68496994	UP

RSU1	1.684557129	UP
PCMT1	1.677965258	UP
KIAA0146	1.649113522	UP
TMEM67	1.643976898	UP
AURKA	1.639594314	UP
UTP6	1.637812047	UP
ZNF277	1.634223919	UP
FAM69A	1.634200616	UP
ZNF773	1.632745878	UP
ZFAND1	1.622993811	UP
MCM4	1.622630866	UP
PIP4K2A	1.617302053	UP
MOSPD3	1.611111111	UP
C9orf46	1.607670164	UP
SYVN1	1.605386594	UP
NCOA4	1.603724524	UP
TUT1	1.602711157	UP
TRAPPC2	1.601421189	UP
NUDT22	1.598397526	UP
YIF1A	1.588521263	UP
COQ2	1.587386356	UP
TPD52	1.585	UP
AHCYL2	1.584738243	UP
ERLIN2	1.573972244	UP
DIDO1	1.566948731	UP
MEN1	1.563243243	UP
EXOSC8	1.556788056	UP
IFRD1	1.533726261	UP
POLR2B	1.532949202	UP
TTC9C	1.531186441	UP
CHCHD3	1.528143539	UP
ZNF772	1.528106509	UP
MAN2B2	1.526217228	UP
BRMS1	1.509703813	UP
XRCC6	1.503737709	UP
