# Supplementary material for: Gene dysregulation by histone variant H2A.Z in bladder cancer
Source: Epigenetics Chromatin. 2013 Oct 16;6:34. doi: 10.1186/1756-8935-6-34 (PMC3853418; doi:10.1186/1756-8935-6-34)
Supplement: Additional file 5: Table S4 — H2A and H2A.Z nucleosomes interacting proteins. [file 1756-8935-6-34-S5.pdf]

Supplementary Table S4. H2A and H2A.Z nucleosomes interacting proteins.

H2A nucleosome	H2A.Z nucleosome
ACTB	ACTB
ACTC1	ACTC1
ATBL2	ACTL6A
BAZ1B	ACTR6
CBX1	ANP32E
CHAF1B	ATBL2
CHD3	BAZ1B
CHD4	BPTF
CTDSPL2	BRD2
DDB1	CBX1
DNMT3A	CBX5
EED	CHD3
EEF1A1	CHD4
GATAD2A	CUL4B
H1F0	DDB1
H2AFV	DIDO1
H2AFX	DMAP1
H2AFY	DNMT3A
H2AFY2	DNMT3B
H2AFZ	EED
HDAC1	EEF1A1
HIST1H1A	EZH1
HIST1H1C	GATAD2A
HIST1H2AA	GATAD2B
HIST1H2AH	H1F0
HIST1H2BA	H2AFV
HIST1H2BC	H2AFY
HIST3H2BB	H2AFY2
HIST3H3	H2AFZ
HIST4H4	HDAC1
HMGA1	HDAC2
HMGA2	HIST1H1A
HMGN1	HIST1H1C
HMGN4	HIST1H1E
HMGN5	HIST1H2AA
INO80B	HIST1H2AH
MECP2	HIST1H2BA
MTA1	HIST1H2BC
MTA2	HIST3H2BB
MTF2	HIST3H3
PARP1	HIST4H4
RAD23A	HMG20A
RAD23B	HMGA1
RAN	HMGA2
RBBP4	HMGN1
RBBP7	HMGN3
RCC1	HMGN4
RPLP0	HNRNPD
RPLP1	INO80B
RPLP2	MECP2
RSF1	MEN1
RUVBL1	MTA1
RUVBL2	MTA2
SCML2	MTF2
SMARCA1	NAP1L1
SMARCA4	PARP1
SMARCA5	PHF14
SSRP1	PWWP2A
STP16	RAD23B
SUZ12	RAI1
TCF20	RAN
TOP1	RBBP4
USP7	RBBP7
XPC	RCC1
XRCC6	RPLP0
	RPLP1
	RPLP2
	RSF1
	RUVBL1
	RUVBL2
	SMARCA1
	SMARCA4
	SMARCA5
	SMARCB1
	SRCAP
	SSRP1
	STP16
	SUZ12
	TCF20
	TOP1
	VPS72
	WDR5
	XPC
	XRCC6
	YEATS4

H2A nuc specific protein

H2A.Z nuc specific protein
