# Supplementary material for: Gene dysregulation by histone variant H2A.Z in bladder cancer
Source: Epigenetics Chromatin. 2013 Oct 16;6:34. doi: 10.1186/1756-8935-6-34 (PMC3853418; doi:10.1186/1756-8935-6-34)
Supplement: Additional file 6: Table S5 — Primer sequences for RT-PCR and ChIP. [file 1756-8935-6-34-S6.pdf]

Supplementary Table S5. Primer sequences for RT-PCR and ChIP.

RT-PCR

**Genes**	**Forward**	**Reverse**
AXL	ACACCCCAGAGGTGCTAATG	ACGAGAAGGCAGGAGTTGAA
BIRC2	CTGGTAGTATGCCAGGAATG	TGTACGAACAGTACCCTTGAT
BIRC3	GCCAGTTACCCTCATCTAC	ATTGCATCTTCTGAATGGTCTTCT
CASP8	CCAGAGACTCCAGGAAAAGAGA	GATAGAGCATGACCCTGTAGGC
CCND1	TCAATGAAGCCAGCTCACAGTGC	TTTGGTTCGGCAGCTTGCTAGG
CDKN2A	ATGGTGCGCAGGTTCTTGGT	CTGGCTCCTCAGTAGCATCA
CKS2	CATTACATGATTCATGAGCCAG	GTTTCAGTAAACTCAAACAGGCAC
CRIP2	CCCACCTGCCAGTGTTATTT	TTGACAGCACAAGGCTCAAC
CYGB	CAGTTCAAGCACATGGAGGA	GTGGGAAGTCACTGGCAAAT
DBC1	GCCTCGACACCTTCTTTG	GCGGTTCTTGTTGCTCTT
DDX43	CCCAGATAGGCAGACAGTTA	ATTGTGCGAGGCGATGAA
FADD	ACGCTTCGGAGGTAGATG	CCTGGTACAAGAGGTTCA
FGF3	CACGAAGTACCACCTCCA	TCCACTGCCGTTATCTCC
FLNC	ATTGAGATCCTGTCGGATGC	GACCTTGACGCCACTGGTAT
FSTL1	CCTGTGTGTGGCAGTAATGG	CCAGCCATCTGGAATGATCT
JAK1	GGATGGACTATTTGGGTTCTC	CACTTGGTGTTCACTCTCA
LAMC3	TTGCAGACACGAGAAAGAAG	GGCACTGGAGGAAAGAGG
LCP1	GTGGCCAGAAGGTCAATGAT	TTTTTCGGGCCATAGAGATG
MEN1	GTGGCCGACCTGTCTATCAT	CCAACCTGTGATGAAGCTGA
NKD2	CCCAGGAGCCAGATACACAT	CGGCAGGTAGTAGCTGAAGG
NT5E	CGCAACAATGGCACAATTAC	CTCGACACTTGGTGCAAAGA
POLDIP2	TGAGGGTGGTGTCTTCTC	TGGCTCCCACTTTGATGT
PPFIA1	TGAAAGTCGAGTTGGCAGTG	CGGGGAGGTTTCACACTTTA
PROS1	CCTAGTGCTTCCCGTCTCAG	TTTCCGGGTCATTTTCAAAG
SRCAP	CCCAAAGGTCACTGGGACTA	AATTGCACCACCTTCTCCAC
STK3	CATGAGGAACAGCAACGAGA	TATCACCATGGTCCCCAAGT
TERT	CGTGGTTTCTGTGTGGTGTC	CCTTGTCGCCTGAGGAGTAG
YAP1	GCATCTTCGACAGTCTTCTTT	TTTGCCATCTCCCAACCT

ChIP

**Genes**		**Forward**	**Reverse**
BIRC2		CAAACCGCAAGGTGAAGA	ATGAACCTCCGGGAAAGAC
BIRC3		CACGAGCAATGAAGCAAA	CTCTTAACCGTGGCATATCT
CCND1		AAGATGCAGTCGCTGAGATT	ATGGGCGCATTTCCAAGA
DDX43		GTCCTGAGGGATATAGTGTC	AGTGGCTCTTCAAAGCAAA
FADD		GCCACGACCTTCTCTTCT	CGTGCCCGGAGTTTACAT
MEN1		AACGAGTGCTGCACACAGAG	GGTCGGCAAGTTATGAGGAA
NT5E		TAGCGCAACCACAAACCATA	TGCAGGAAGAGTGGAGAGGT
POLDIP2		CCCTCAGCCTTTGGATTT	CACCCAAGAGTCTCGTTTC
PPFIA1		GACCAGCTCGACCAACAT	ACGCCACCAAGTCCAGATAA
STK3		GGTCCGAGTTCCCAGAGTTT	CTCCCCGATTCGTTACCTCT
STX1A		AGTCCGGGGTCCTGGATA	CACGGGAGGTCAGAGATGAG
TMEM123		GAACCCGTGGAGAAGATGAA	GTGAGAGGGAGGAGTTGCAG
TRA2B		TTTCGATCTCCTGACCTCGT	CCCCCAATCTCAATTCCTTT
TRPC6		ACCGCCTCCTGAAAGTTGTGGCTA	TTCATCCCCCGCACTCTCCGTC
USP10		AGCACTTTACGCGTGCTTTT	CGGGAGTGTTCGCATTAGAT
YAP1		CACAAGCCTTTGATAATACAC	TCATCGCTTCCCAAACAT
BIRC3			
	UR	TTGACCGCTGAACAACAC	CTGGGCTCTCCTTTGACT
	TSS	CACGAGCAATGAAGCAAA	CTCTTAACCGTGGCATATCT
	CR	AGGTTCCTGCTCAATGATGTC	ATGCCCTATGCACCTCTTC
CCND1			
	UR	TTGCTCCGAGCTTTCCAG	CGTGACCATTCCACCTTC
	TSS	AAGATGCAGTCGCTGAGATT	ATGGGCGCATTTCCAAGA
	CR	ATATCGGCTTGGAGGACCTT	GAGCCCCAGGAATTCAAACT
CASP8			
	UR	TGAGTCTCGTGCCTTGAGTG	GAATTATGGCCAGGCACAGT
	TSS	GGGTCTAGGGCTCAGAGCTT	GTGTTCTGGCTCAGCCCTAC
	CR	CTACATTCCGCAAAGGAAGC	TTCCCTTTCCATCTCCTCCT
